# Hyperspectral full-field quick-EXAFS imaging at the ROCK beamline for monitoring micrometre-sized heterogeneity of functional materials under process conditions

**DOI:** 10.1107/S1600577524006581

**Published:** 2024-08-23

**Authors:** Valérie Briois, Jean Paul Itié, Alain Polian, Andrew King, Aliou Sadia Traore, Eric Marceau, Ovidiu Ersen, Camille La Fontaine, Laurent Barthe, Anthony Beauvois, Olga Roudenko, Stéphanie Belin

**Affiliations:** a Synchrotron SOLEIL, L’Orme des Merisiers, Départementale 128, 91190Saint-Aubin, France; b Centre National de la Recherche Scientifique UR1 France; c IMPMC, Sorbonne Université, CNRS-UMR 7590 4 Place Jussieu 75005Paris France; d IPCMS Strasbourg France; e UCCS Lille, France; University of Turin, Italy

**Keywords:** quick-EXAFS, hyperspectral imaging, MCR-ALS, catalysis, electrode materials

## Abstract

Full-field transmission X-ray microscopy has been recently implemented at the hard X-ray ROCK–SOLEIL quick-EXAFS beamline, adding micrometre spatial resolution to the second time resolution characterizing the beamline. Benefiting from a beam size versatility due to the beamline focusing optics, full-field hyperspectral XANES imaging has been successfully used at the Fe *K*-edge for monitoring the pressure-induced spin transition of a 150 µm × 150 µm Fe(o-phen)_2_(NCS)_2_ single crystal and the charge of millimetre-sized LiFePO_4_ battery electrodes. Hyperspectral imaging over 2000 eV has been reported for the simultaneous monitoring of Fe and Cu speciation changes during activation of a FeCu bimetallic catalyst along a millimetre-sized catalyst bed. Strategies of data acquisition and post-data analysis using *Jupyter* notebooks and multivariate data analysis are presented, and the gain obtained using full-field hyperspectral quick-EXAFS imaging for studies of functional materials under process conditions in comparison with macroscopic information obtained by non-spatially resolved quick-EXAFS techniques is discussed.

## Abstract 2

Full-field hyperspectral X-ray absorption spectroscopy imaging implemented at a quick-EXAFS beamline offers the capability to add micrometre-scale information to second time resolution for *operando* monitoring of functional materials under process conditions.

## 1. Introduction

The high time resolution provided by quick-EXAFS (quick-extended X-ray absorption fine structure) beamlines operating on third-generation synchrotron radiation facilities makes the technique powerful for monitoring phase transformations or structural changes occurring during studies of catalysts or batteries at work (Briois *et al.*, 2016 ▸; La Fontaine *et al.*, 2020 ▸; Müller *et al.*, 2016 ▸; Caliebe *et al.*, 2019 ▸). The macroscopic properties monitored during *operando* quick-EXAFS characterization, such as the catalytic activity measured by mass spectrometry or electrode charge capacity, reflect the global modification of the entire device, whether it is composed of a few-millimetres-long catalyst beds or a few tens of millimetres-squared electrode surface. On the other hand, the beam size provided at quick-EXAFS facilities, usually in the range of hundreds of micrometres, allows study of a small fraction of the device, sometimes leading to a misunderstanding of the structure–activity relationship obtained by quick-EXAFS due to heterogeneity along the device (Grunwaldt *et al.*, 2009 ▸; Kalz *et al.*, 2017 ▸; Nakamura *et al.*, 2014 ▸).

Adding space resolution for the characterization of catalytic reactions or electrochemical performances of batteries has received particular attention over the last decade at both the micrometre (Grunwaldt *et al.*, 2009 ▸; Tanida *et al.*, 2011 ▸; Wang *et al.*, 2014 ▸; Nowack *et al.*, 2016 ▸; Becher, Weber *et al.*, 2021 ▸; Becher, Ferreira Sanchez *et al.*, 2021 ▸; Alizadehfanaloo *et al.*, 2021 ▸) and nanometre scales (Meirer *et al.*, 2011 ▸; Wei *et al.*, 2020 ▸), and is of prime importance to put into perspective the overall response of the device, and the existence of heterogeneities in space and/or time within the device. Spatially resolved acquisition strategies are based either on full-field (FF) transmission X-ray microscopy, which utilizes pixel array cameras for adding spatial resolution across the illuminated sample area, or on scanning X-ray transmission microscopy, which takes advantage of the small beam size of the beamline to provide spatial resolution (Grunwaldt & Schroer, 2010 ▸). In the former approach, the field of view (FoV) is limited by the sensor and beam sizes, whereas for scanning X-ray microscopy there is no inherent FoV limitation; however, when targeting a larger FoV with a smaller beam, longer acquisition times are expected which could be detrimental for dynamic monitoring of reaction processes. Irrespective of the strategies employed, a stack of 2D images depicting the X-ray intensity transmitted by the sample at different energies along the X-ray absorption spectrum (XAS) of the element of interest is acquired. This stack of 2D images, which is hereafter referred to as a hyperspectral cube or simply a cube, is then processed to generate chemical speciation maps.

Acquisition of hyperspectral cubes with second time resolution is mandatory for dynamically addressing the chemical transformations undergone by catalytic materials or battery electrodes. To achieve such a time resolution, two strategies can be proposed. Applying sparsity in the energy acquisition of the stack of images is sometimes proposed as a strategy to efficiently limit the time required for exploring dynamically the heterogeneity of a sample. In that case, the selection of a few energies, for which a great variance of absorption is observed among the different phases allowing unambiguous differentiation between them, is preferred over recording the full stack of energies. Such a strategy has been demonstrated as being efficient for both scanning (Zhang *et al.*, 2023 ▸) and full-field X-ray transmission microscopy (Nowack *et al.*, 2016 ▸). The second approach allows the reconstruction of well resolved energy XAS spectra, providing more complete XANES (X-ray absorption near-edge structure) and eventually EXAFS fingerprints. For FF imaging, the implementation of a microscope at a quick-EXAFS beamline with full optimization for triggering of absorption images in the fly scan scheme of acquisition (Renaud *et al.*, 2009 ▸) is a strategy that can be well adapted for shedding light on the dynamics of transformations at the micrometre scale of many chemical systems exhibiting spatial heterogeneity (Alizadehfanaloo *et al.*, 2021 ▸).

In this paper, we describe the capability of hyperspectral FF quick-EXAFS 2D imaging at the micrometre scale with second time resolution implemented at the quick-EXAFS ROCK–SOLEIL beamline (Briois *et al.*, 2016 ▸; La Fontaine *et al.*, 2020 ▸). The performances will be illustrated using three case studies of functional materials under process conditions: a molecular complex for which the spin transition is pressure-induced, battery electrodes for which the 1 C charge is dynamically monitored, and heterogeneous bimetallic catalysts for which the activation is reported at the *K*-edge of both metals. These examples were chosen to illustrate the versatility of the beamline in varying the FoV used for the FF imaging. The strategy of post-data analysis leading to chemical speciation will also be discussed, depending on the information the user wishes to extract: good spatial resolution with moderate time resolution or vice versa. The use of the multivariate curve resolution alternating least-squares (MCR-ALS) fitting approach, nowadays in common use at the time-resolved quick-EXAFS ROCK beamline (Passos *et al.*, 2023 ▸; Cassinelli *et al.*, 2014 ▸; Lesage *et al.*, 2019 ▸; Eveillard *et al.*, 2020 ▸) for solving the mixture problem encountered during quick-EXAFS reaction monitoring, will be discussed in relation to the big data issue encountered when time and space resolution are used simultaneously.

## 2. Experimental

### 2.1. Hyperspectral FF X-ray transmission microscope implemented at the ROCK quick-EXAFS beamline

**Figure 1:**
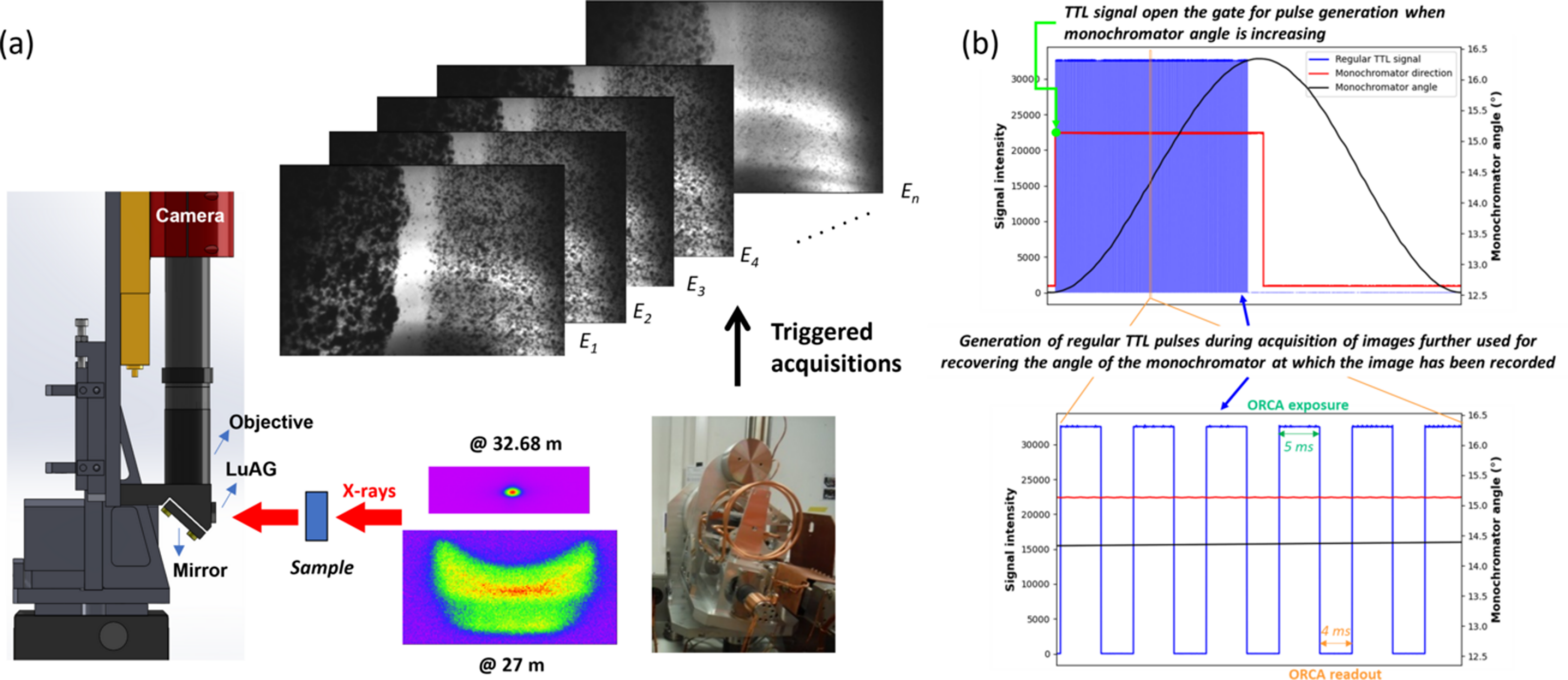
(*a*) Schematic representation of the home-made transmission X-ray microscope implemented at the ROCK beamline at Synchrotron SOLEIL. (*b*) Principle of triggering the ORCA Flash4.0 V3 CMOS camera for recording a stack of images during a half-period of oscillation of the monochromator and of the correspondence between image and Bragg angle of the monochromator used for the energy recovery.

The FF imaging method implemented at the ROCK beamline [Fig. 1 ▸(*a*)] is based on the recording of a stack of magnified two-dimensional transmission images at different energy values across the absorption edge of the absorbing element of interest using a pixelated ORCA Flash4.0 V3 digital CMOS camera (Hamamatsu) coupled with a thin LuAG:Ce scintillator (from CRYTUR). The scintillator, operating with lights off inside the experimental hutch, converts X-rays into visible light, which is reflected by a mirror towards the magnifying microscopy objective. The edge trigger mode of the camera is used for performing image exposure synchronized by a counter/timer card (National Instruments PXI 6602) acting as external trigger with active gate. The TTL signal defining the change of direction of the monochromator angle (Fonda *et al.*, 2012 ▸) is used by the PXI 6602 card to gate the counting process for acquiring images at the onset of the ascending angle half oscillation period of the monochromator [Fig. 1 ▸(*b*)]. Subsequently, the counter counts TTL pulses of 9 ms encompassing the camera exposure time (typically 5 ms) and the camera readout time (typically 4 ms). The number of pulses, and therefore of images in the hyperspectral stack, is set to 500 and 580 images for 0.078 Hz and 0.090 Hz oscillation frequency of the monochromator, respectively [Fig. 1 ▸(*b*)]. The total number of recorded images in a cube is intentionally lower than the theoretical number which could be calculated considering the exposure and readout times per image and the time of the half ascending oscillation period. This choice allows us to eradicate the recording of images with oversampled energy as a result of monochromator deceleration before direction change and to avoid any recording of images after the monochromator direction change. The images are stacked in a hyperspectral cube with the edf file format (edf stands for European data format) and associated with the energy values at which they were acquired thanks to synchronous recording of a NeXus file containing the triggered pulse signals and encoder values of the monochromator Bragg angle positions. Hyperspectral cubes are recorded continuously while the quick-EXAFS monochromator is oscillating, with a repetition rate every 12.8 to 11.1 s, depending on the oscillation frequency. The amplitude of the channel-cut crystal oscillation, in addition to defining the energy range of the spectrum, will determine the energy step separating consecutive images in the hyperspectral cubes. The smaller the oscillation amplitude, the better the energy resolution is for the spectra obtained, as illustrated in Fig. S1 of the supporting information for the three case studies presented in the paper. The procedure of data acquisition is controlled by the *Tango* software bus interfaced with Python scripting, making the acquisition procedure fully automated.

The pixel size of the ORCA Flash 4.0 V3 is 6.5 µm and the sensor array is made of 2048 × 2048 pixels which is reduced to 2048 (H) × 748 (V) pixels for hyperspectral imaging. Depending on the magnifying microscopy objective mounted on the camera, the pixel size and FoV range from 0.65 µm to 1.625 µm and 1.3 mm to 3.3 mm, respectively, as detailed in Table 1 ▸. At the ROCK beamline, the size of the beam can be continuously varied horizontally from ∼0.5 mm to 3.3 mm full width at half-maximum (FWHM) by moving the experimental table from the focus point of the toroidally focusing first mirror positioned 32.68 m downstream of the ROCK bending-magnet source up to about 5 m upstream from the focus position, and vertically from ∼0.3 mm to 2 mm by changing the curvature of the vertically focusing M2B mirror placed after the monochromators (Briois *et al.*, 2016 ▸). Considering that the ROCK’s channel-cut monochromators are not fixed-exit, the vertical size of the beam is carefully optimized to ensure the sample is always illuminated during the oscillation amplitude of the channel-cut crystal. This versatility of the beam size perfectly matches the versatility of the FoV which can be achieved by transmission X-ray microscopy by changing the magnification of the objective.

**Figure 2:**
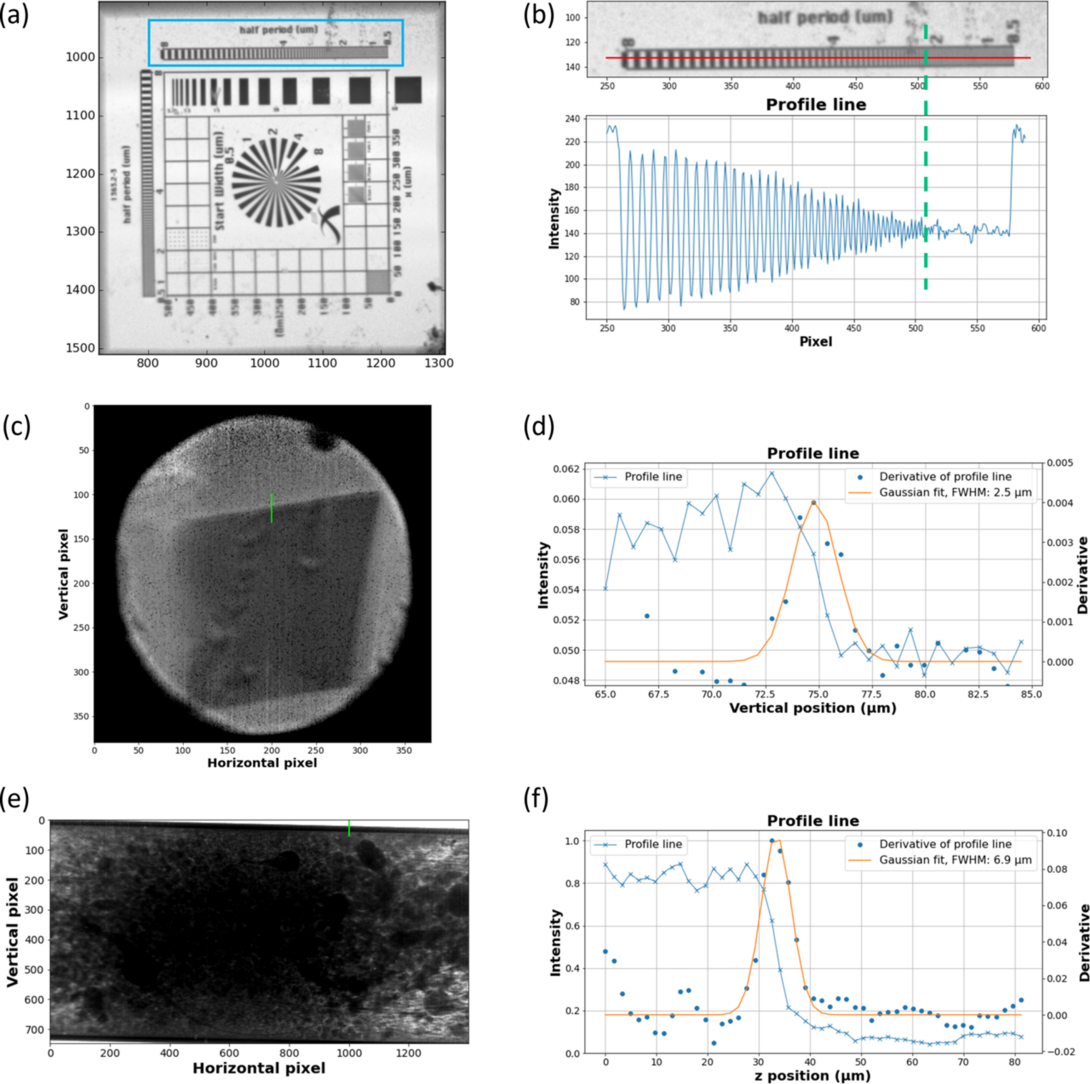
Spatial resolution evaluated from the Xradia resolution pattern and from the edge profile line of the sample. (*a*) Normalized (dark image and flat-field-corrected) projection of the Xradia resolution pattern (Zeiss) recorded at 7225 eV using a 5× magnifying Mitutoyo objective with a pixel size of 1.3 µm × 1.3 µm at 8 mm from the LuAG:Ce scintillator and (*b*) zoom (top) of the top horizontal bar boxed in blue in (*a*): the black lines are spaced from each other by a calibrated distance, ranging from 8 to 0.5 µm. The moment at which the black ticks cannot be distinguished from each other indicates the spatial resolution limit. A profile (bottom) of the intensity of the red line displayed in the zoom. The green dashed line indicates the pixel index at which the profile line no longer exhibits regular oscillation revealing the limit of spatial resolution. Edge profile line of the sample for (*c*, *d*) case study 1 and (*e*, *f*) case study 3. For both cases the image on the left represents the absorption map at 7159 eV corrected from the flat-field and the electronic noise of the camera. The procedure to estimate the spatial resolution consists of plotting a profile line somewhere in the absorption map where an abrupt change of the contrast was observed. This was done on the edge of the spin transition single crystal in (*c*) and on the wall of the quartz capillary containing the catalyst in (*e*) crossed by the green line on the absorption maps. The intensity along this line relative to the pixel number, converted into micrometres according to the size of the pixel reported in Table 1 ▸, is then plotted in (*d*) and (*f*) (blue line with crosses) and the derivative of this curve is calculated (blue circle). The spatial resolution was estimated to be ∼2.5 µm and ∼6.9 µm by calculating the FWHM of the derivative fit by a Gaussian model (orange line) for case studies 1 and 3, respectively.

The spatial resolution achieved for each case study has been evaluated by considering either an Xradia resolution pattern (Fig. S2) placed at a distance from the scintillator comparable with that used for the samples or the edge profile line (Smith, 1999 ▸) of the samples when possible. The closest the camera is to the sample (8 mm), the better the spatial resolution, which is evaluated in Fig. 2 ▸ to be around 2–3 µm, irrespective of the FoV matching the beam size. For a distance of 20 mm between the scintillator and the sample, which is a good compromise for bulky sample environments, the spatial resolution is evaluated to be around 4 µm using the Xradia pattern and ∼7 µm using the edge profile line. Differences between both evaluations for case study 3 could result from the fact that the capillary edge and the Xradia pattern were not in the same focus plane of the camera considering the cylindricity of the capillary and the flat shape of the pattern.

### 2.2. FF hyperspectral data acquisition and data analysis

**Figure 3:**
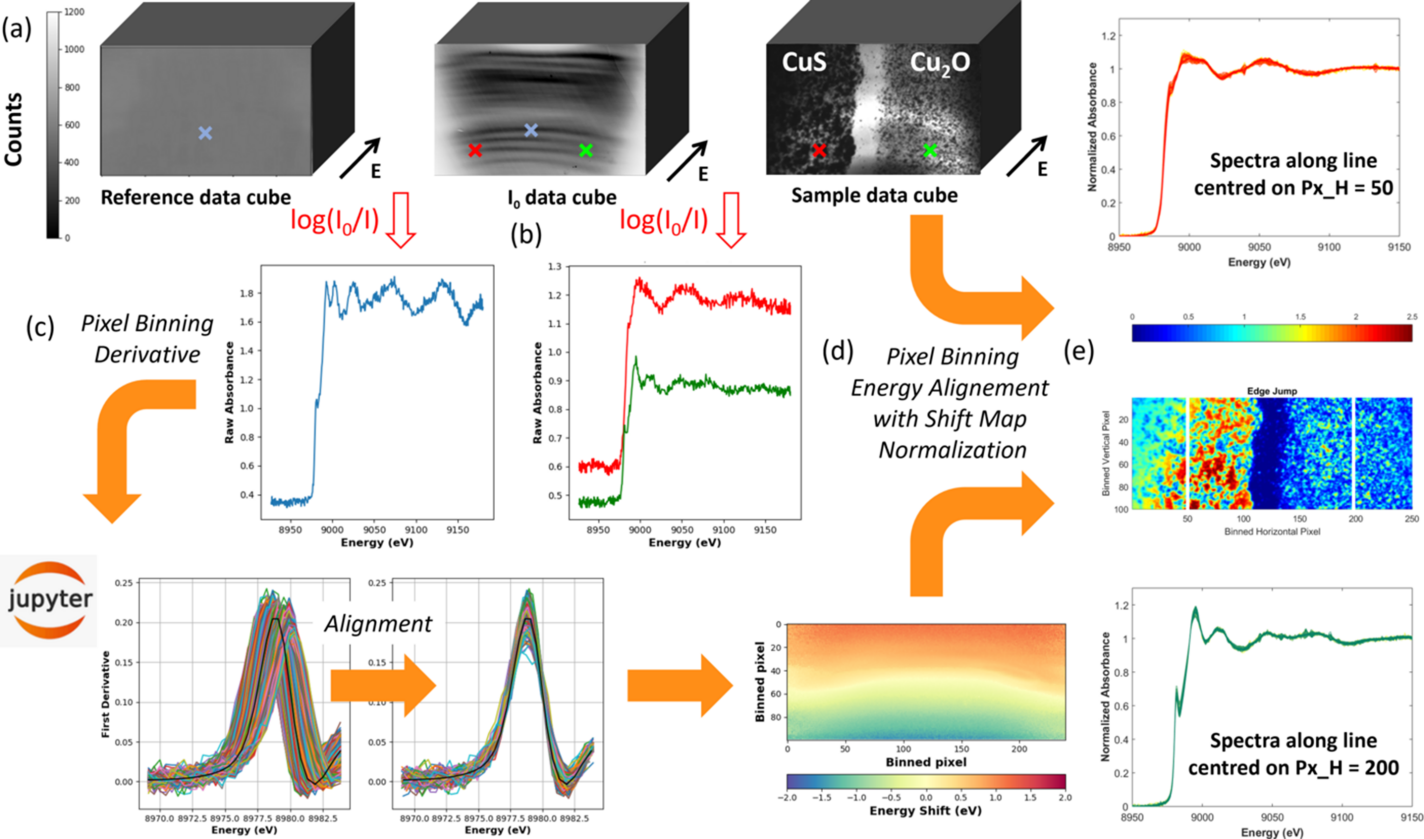
Summary of hyperspectral acquisition strategy and subsequent post-treatment analysis for a Cu *K*-edge dataset recorded for a sample prepared from the juxtaposition of a pellet of CuS (on the left) and a pellet of Cu_2_O (on the right). (*a*) At the same position of the camera sensor, hyperspectral cubes of a reference Cu metallic foil, of the flat-field image for *I*_0_ and of the sample of interest are recorded successively. (*b*) Using the Beer–Lambert law, the spectra of each pixel of the reference image (blue cross) and of the sample image (green and red crosses) can be calculated. (*c*) After pixel binning, the first derivatives of the spectra of the Cu reference foil hyperspectral cube are calculated and realigned to the first derivative (black curve) of the quick-EXAFS spectrum of the reference foil absolute energy calibrated. An energy-shift map is therefore obtained consisting at each pixel of the Δ*E* offset value used for absolute energy scale alignment. (*d*) The energy shift map is used for energy alignment at each pixel of the spectra of the sample hyperspectral cubes, performed after pixel binning similar to the one used for the reference and before normalization. (*e*) Outcomes of the sequence of *Jupyter* notebooks visualized along vertical lines of pixels in the CuS and Cu_2_O areas located in the edge jump map (white lines) for which values are related to the color scale displayed at the top of the map.

We will illustrate in this paper the convenience of the versatility of the beam size for imaging samples with sizes ranging from ∼200 µm for single crystals of spin-crossover compounds to ∼3 mm for electrode materials or catalyst beds (Table 1 ▸). However, the use of defocused beams within the optical layout characterizing the beamline (Briois *et al.*, 2016 ▸) adds complexity for hyperspectral imaging arising from an increase of the energy bandwidth in the beam footprint. For instance, as detailed in Fig. S3 of the supporting information, at the Cu *K*-edge the energy dispersion in the footprint compared with the absorption-edge position of spectra measured at the center of the FoV can range from Δ*E* = ±0.4 to ±1.5 eV depending on the in-vacuum filters used after the first mirror to reduce the heat load on downstream optics, in particular the monochromators. To overcome this issue, which is prejudicial for monitoring tiny changes in energy positions of spectra resulting from redox reactions, we developed strategies in both data acquisition and data post-treatment. This encompasses, firstly, the systematic measurement of hyperspectral cubes for a reference foil, characteristic of the absorbing element of interest, during the mandatory sequence of acquisition of flat-field cubes (for *I*_0_) and sample hyperspectral cubes, the three being acquired at the same position on the camera sensor, and, secondly, using *Jupyter* notebooks, the determination from the reference foil of a shift map energy alignment in each binned pixel of the image which is subsequently transferred to the sample binned images, as displayed in Fig. 3 ▸. The complete workflow from raw data to post-treated hyperspectral cubes using interactive *Jupyter* notebooks is summarized in Fig. S4 of the supporting information and described in depth by Briois *et al.* (2024 ▸). HDF5 files containing the normalized spectra calculated on each binned pixel, the edge jump image, the energy shift map and the mask used to define the sample location on the image are finally saved.

To eradicate monochromator energy drift during hyperspectral cube acquisition and allow the merging of successive cubes for improving the signal-to-noise (S/N) ratio of XAS spectra recorded for steady-state conditions, an energy pre-alignment of each cube is performed during step 2 of data extraction (Fig. S4) considering a glitch of the monochromator measured with an ionization chamber placed before the sample during hyperspectral imaging measurements, as shown in Fig. S5(*a*) of the supporting information.

FF X-ray microscopy imaging in comparison with scanning X-ray microscopy imaging presents the disadvantage that flat-field (for *I*_0_) and sample hyperspectral cubes are not recorded simultaneously. To properly normalize the transmitted sample intensity in each pixel of the image and obtain its absorbance as a function of energy using the Beer–Lambert law, the flat-field images must be recorded for each sample at the same position of the pixel array camera sensor each time the camera is moved. Such a recording is usually performed before starting the acquisition of the sample cubes, to avoid the recording of sample cubes unusable for the normalization in the case of beam failure in the storage ring. Flat-field images at the end of the hyperspectral monitoring of the dynamic transformation are also recorded for verifying the repeatability of the former measurement, as a result of the beam stability, the latter being ensured by the top-up injection mode and active orbit feedbacks in operation at SOLEIL (Couprie *et al.*, 2013 ▸). The 1% of intensity variation of the incident beam inherent to the top-up injection mode of the machine occurring every 2 min is minimized by merging several *I*_0_ cubes over a time encompassing the top-up; typically 20 cubes are merged in order to obtain flat-field images that can be used for the normalization.

The normalized spectra recorded at each binned pixel of the images are further used to determine the sample composition by using the multivariate curve resolution with alternating least-squares (MCR-ALS) fitting approach (de Juan *et al.*, 2014 ▸). Home-made MATLAB scripts are used for reading the HDF5 files and for unfolding the hyperspectral imaging cube into a two-dimensional experimental data matrix **D** mandatory for applying the bilinear decomposition of spectra into a matrix of concentration **C** and a matrix **S** of pure spectra of species using the MCR-ALS GUI 2.0 developed by Tauler *et al.* on the MATLAB platform (Jaumot *et al.*, 2015 ▸).

### 2.3. Experimental conditions for the pressure-induced spin transition in the Fe(o-phen)_2_(NCS)_2_ complex

A single crystal of Fe(o-phen)_2_(NCS)_2_ (o-phen = ortho-phenanthroline) of size ∼150 µm × 150 µm was loaded inside a diamond anvil cell (DAC) equipped with thin diamonds (1 mm each) optimized for 1% of transmission at the Fe *K*-edge (7112 eV), a 800 µm culet and a 60 µm-thick CuBe gasket with a 400 µm drilled hole and silicon oil pressure-transmitting medium (Gaspar *et al.*, 2018 ▸). A ruby crystal was also loaded inside the DAC sample chamber near to the single crystal to be used as an internal standard for pressure calibration by measurement of its fluorescence lines under laser irradiation. The pressure inside the DAC was increased using an inflatable gas membrane remotely controlled by a Pace controller. Hyperspectral cubes were measured at steady-state pressures of 0, 0.46, 0.50, 0.63 and 3.40 GPa, and during pressure increases from 0.63 to 3.40 GPa applying pressure on the membrane at 0.01 bar s^−1^.

The grazing incidence of the pink and monochromatic beams on the harmonic rejection mirrors with B_4_C stripes was 3.2 mrad. A Si(111) channel-cut monochromator was used with an oscillation amplitude set to 0.5° around a 16.05° Bragg angle at an oscillation frequency of 0.078 Hz. This allows the measurement of 500 images between 7075 eV and 7230 eV in 6.4 s every 12.8 s. The energy step between successive images is in the range 0.25–0.5 eV depending on the Bragg angle position on the half-rising period of the monochromator oscillation [Fig. S1(*a*)]. The ORCA camera was equipped with a 10× Mitutoyo magnifying microscopy objective [Plan Apo, numerical aperture (NA) = 0.28] leading to a pixel size of 0.65 µm. 100 µm-thick LuAG:Ce was used as scintillator. To eradicate Bragg diffraction peaks of the diamonds, the cell was slightly tilted from normal incidence of the X-ray beam.

More details about the data analysis procedure are given in the supporting information (Figs. S6 to S10 and Table S1).

### 2.4. Experimental conditions for the *operando* cycling of LiFePO_4_ battery composite electrodes

Self-supporting LiFePO_4_ electrodes (hereafter referred as LFP) with a 6 mm diameter were prepared from a mixture of 84.4% LFP (from SOC P2N) (with 0.9% carbon coating), 10% Super P carbon black and 5.4% polytetra­fluoro­ethyl­ene binder. Li metal was used as the negative electrode and 1 *M* LiPF_6_ in ethyl­ene carbonate/di­ethyl carbonate with 1/1 ratio as the electrolyte soaked in one layer of Whatman glass fiber used as separators. The charge of the LFP electrodes has been imaged using an electrochemical cell developed at SOLEIL thanks to the ANR PULSSE funding (Leriche *et al.*, 2010 ▸). The electrochemical cell was assembled in transmission geometry in an Ar-filled glovebox and the Be cathode window was protected by a 4 µm-thick sheet of ultra-pure aluminium foil. These half-cells were connected to a galvanostat/potentiostat (VMP3 of Biologic) and charge rates to 1 C ≃ 180 mA g^−1^ of active material, *i.e.* charge in 1 h, were applied in galvanostatic mode with potential limitation (4.2 V versus Li/Li^+^) (Figs. S11 and S12).

The same optics and alignment settings as those used for the spin transition study and previously described in Section 2.3 were chosen for the LFP study. The only difference was the position of the experimental table located 3.8 m downstream of the focus point, a position that has been chosen not only to enlarge the horizontal FoV on the electrode to nearly 2.66 mm but also to minimize the photon density on the electrodes which are known to be sensitive to radiation damage (Blondeau *et al.*, 2022 ▸; Jousseaume *et al.*, 2023 ▸). Vertically, the beam was focused by the M2B mirror leading to a size of 0.45 mm. The ORCA camera was equipped with a 70 µm LuAG:Ce scintillator and a 5× Mitutoyo magnifying microscopy objective (Plan Apo, NA = 0.14) leading to a pixel size of 1.3 µm. The scintillator was located at about 7–8 mm from the electrode. The region of interest (RoI) over the camera sensor was 2048 (H) × 348 (V). With a cube recorded every 12.8 s, the deintercalation rate per cube for a 1 C charge was −0.0036 Li.

The data analysis procedure is detailed in the supporting information (Figs. S13 to S19 and Table S2).

### 2.5. Experimental conditions for the activation of a FeCu bimetallic catalyst

The activation by H_2_ of a bimetallic FeCu catalyst used for the hydro­conversion of organic molecules was monitored by FF hyperspectral imaging, simultaneously at the Fe and Cu *K*-edges. The catalyst was prepared by Fe^III^ and Cu^II^ nitrate impregnation onto Sipernat-50 silica. The loading after reduction was 10 wt% for each metal. After drying in an air flow at 100°C for 16 h, the impregnated system was calcined under synthetic air up to 500°C with a ramp rate of 5°C min^−1^ and 5 h of isothermal heating at 500°C. The activation of the calcined catalyst was carried out at ROCK using a gas blower which heated a 1.2 mm-internal-diameter quartz capillary in which 10 mg of catalyst was loaded. The catalyst bed was ∼4 mm long and maintained with glass wool at the center of the capillary reactor, above the nozzle of the gas blower. The activation was carried out under 10 ml min^−1^ H_2_ mixed with 3 ml min^−1^ N_2_, at a heating rate of 10°C min^−1^ up to 400°C.

The grazing incidence of the pink and monochromatic beams on the harmonic rejection mirrors with B_4_C stripes was 2.8 mrad. A Si(111) channel-cut monochromator was used with an oscillation amplitude set to 3.92° around a 14.5° Bragg angle at an oscillation frequency of 0.09 Hz. This allows the measurement of 580 images between 7070 and 9090 eV in 5.56 s every ∼11.1 s. The energy grid at which the images are recorded for this two-edge experiment is displayed in Fig. S1(*b*). Considering that more images are recorded during the accelerating and decelerating phases of the channel-cut monochromator, after and before changing of oscillation direction, the energy step in these angular ranges is greatly reduced allowing the recording of XANES with a reasonable energy resolution at both extremes of the energy domain, at the Fe *K*-edge (7112 eV) and at the Cu *K*-edge (8979 eV), and Fe *K*-edge EXAFS spectra with a sparser energy step between consecutive images. The ORCA camera was equipped with a 4× Navitar magnifying microscopy objective (Plan Apo, NA = 0.2), leading to a pixel size of 1.625 µm, and a 70 µm LuAG:Ce scintillator.

The data analysis procedure is detailed in the supporting information (Figs. S20 to S30 and Table S3).

Table 2 ▸ summarizes (for the purpose of comparing between the three case studies) the main recording parameters used by FF imaging for acquiring one spectrum. It is noteworthy that standard quick-EXAFS can always be recorded, alternately with FF hyperspectral imaging, using the ionization chamber still in place before and after the sample. In that case the camera must be moved out of the way of the beam to allow X-rays to pass to the second ionization chamber, as detailed in Fig. S5(*b*). The time resolution obtained by standard quick-EXAFS for the case studies presented here is also summarized in Table 2 ▸. For the spin transition case study, the size of the single crystal was so small that it was necessary to close a lot the primary slits before the first ionization chamber, and the DAC was so absorbing that we wasted no time in scanning the position of the DAC to find the sample inside the pressure chamber and measuring it by standard quick-EXAFS. It is worth mentioning that, every time the camera is moved out of the way of the beam for standard quick-EXAFS acquisition, the recording sequence of cubes for the flat-field, the reference and the sample must be repeated, when moving back to FF imaging acquisition.

## 3. Results

### 3.1. Study of the pressure-induced spin transition in the Fe(phen)_2_(NCS)_2_ complex

The Fe(o-phen)_2_(NCS)_2_ sample belongs to the family of iron spin-crossover complexes for which spin-state bistability under temperature or pressure changes has been extensively studied in the last three decades by XAS (Briois *et al.*, 1995 ▸; Roux *et al.*, 1996 ▸; Boillot *et al.*, 2002 ▸), taking advantage of the high sensitivity of the technique to the electronic and structural local order modifications induced by spin conversion. Those studies give access to the fraction of high-spin (HS) and low-spin (LS) converted under stimuli and highlight the kinetics of spin crossover. Publications relating to the spatio-temporal behavior of the spin crossover are rather scarce and, to the best of our knowledge, report only the spatio-temporal dependence of thermally induced spin transitions observed by optical microscopy (Traiche *et al.*, 2017 ▸; Fourati *et al.*, 2019 ▸) or Raman imaging (Bedoui *et al.*, 2010 ▸).

**Figure 4:**
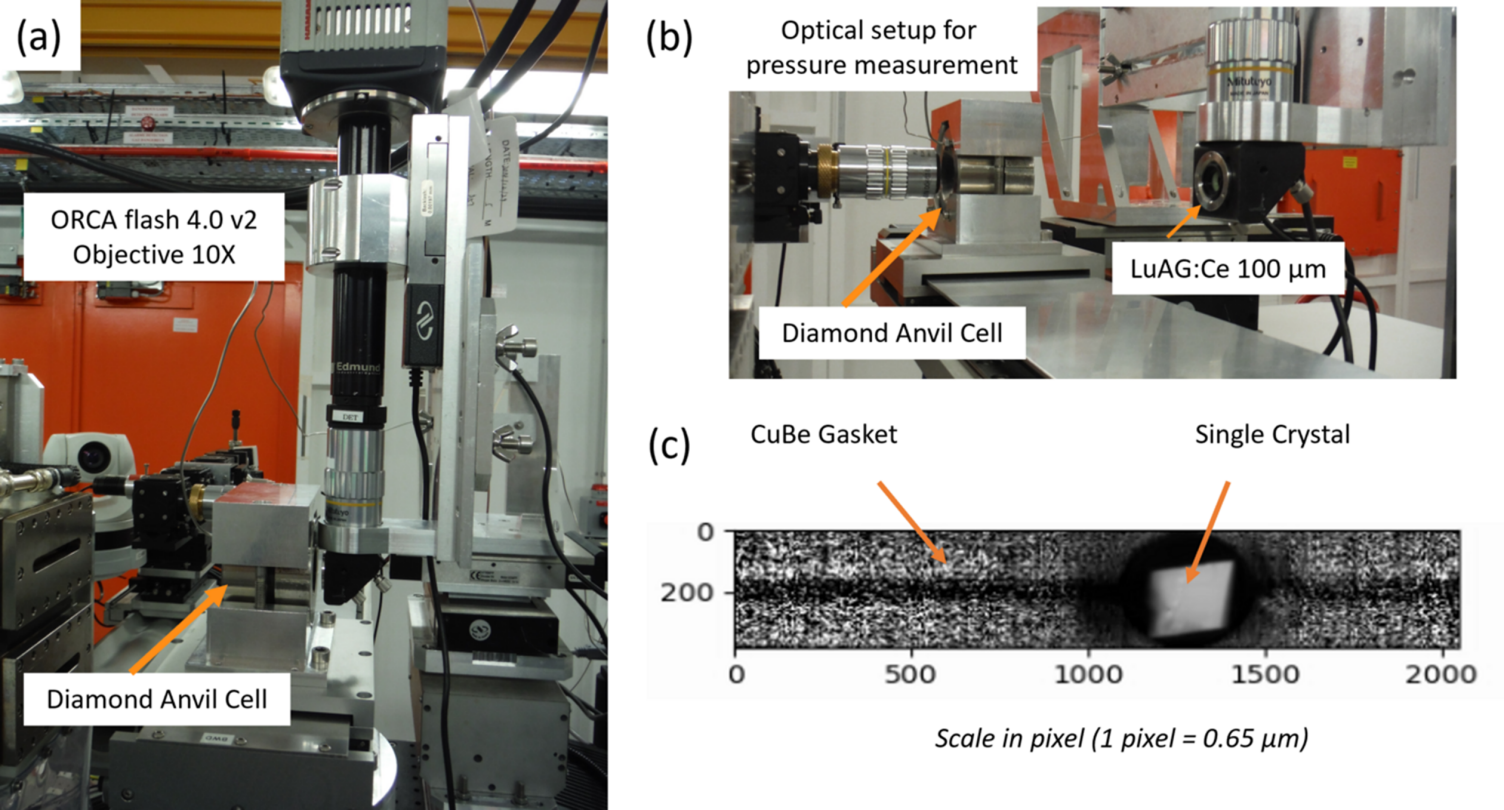
(*a*) Image of the setup showing the DAC and the 10× Mitutoyo magnifying microscopy objective mounted in front of the ORCA camera. (*b*) The DAC can be translated using a large stroke motorized actuator in front of the optical setup used for the ruby fluorescence measurement. (*c*) Absorption contrast image of the single crystal at ambient pressure inside the DAC obtained by dividing the intensity measured at a given energy after the edge energy by the intensity measured at a pre-edge energy.

Herein, we report for the first time hyperspectral FF XAS imaging developed at the ROCK beamline for studying the spatio-temporal dependence of pressure-induced spin transitions on an Fe(o-phen)_2_(NCS)_2_ single crystal. The set-up used for this first case study is displayed in Fig. 4 ▸, with the DAC located at the focusing point of the ROCK optics (Briois *et al.*, 2016 ▸) providing a photon beam size of 330 µm (H) × 140 µm (V) FWHM. Fig. 4 ▸(*c*) shows the single crystal illuminated by X-rays passing through the anvils and the gasket drilled hole.

**Figure 5:**
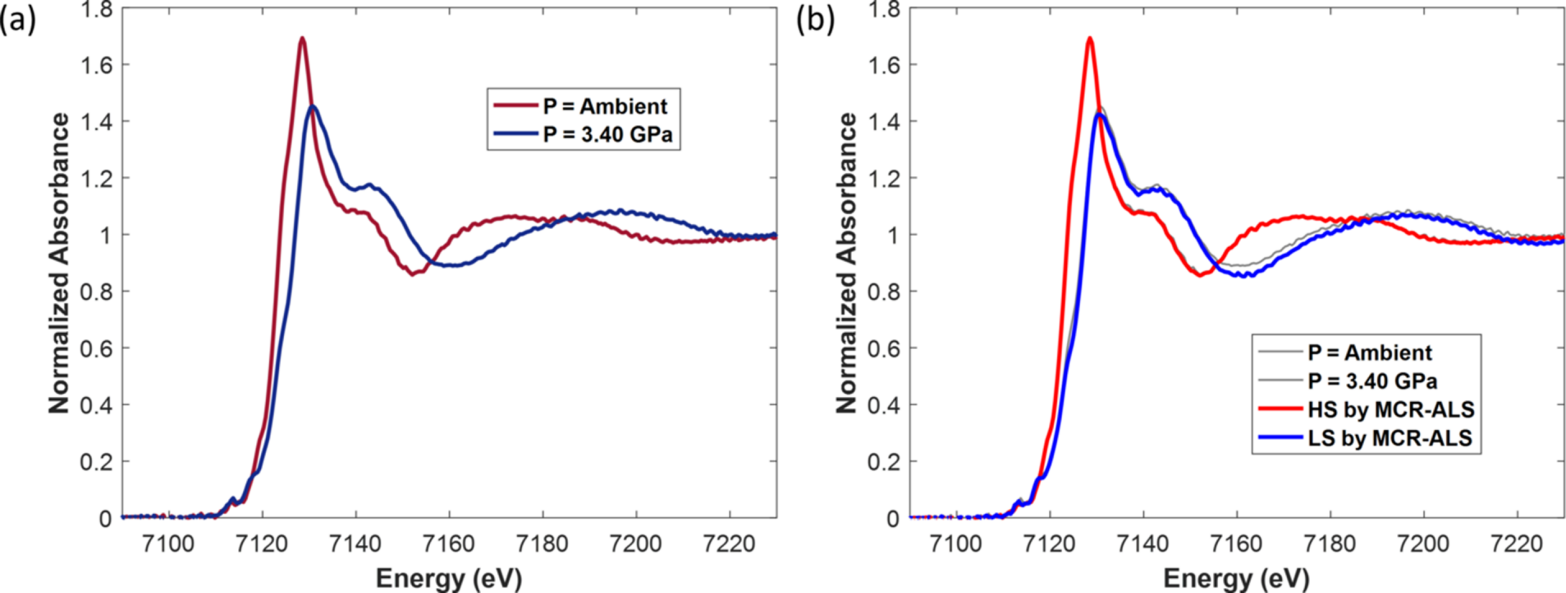
(*a*) XANES spectra recovered by merging all the pixels of the images recorded at room temperature under ambient pressure and 3.40 GPa, respectively. (*b*) Pure spectra corresponding to the HS and LS forms of the spin crossover complex isolated by MCR-ALS. For comparison purposes, the experimental spectra shown in (*a*) are displayed in gray color.

Previous studies report that the pressure required for the conversion of a half fraction of HS to LS ranges between 0.60 and 1.35 GPa (Roux *et al.*, 1996 ▸). Therefore, spectra, displayed in Fig. 5 ▸(*a*), which have been recovered from hyperspectral images by considering all the pixels measured at *P* = 0 GPa and 3.40 GPa, are accordingly characteristic of the HS and LS forms of the Fe(o-phen)_2_(NCS)_2_ complex, respectively. The spectra are in good agreement with those published in the literature (Briois *et al.*, 1995 ▸; Roux *et al.*, 1996 ▸; Mebs *et al.*, 2015 ▸). Modifications in the position of the Fe *K* rising edge and intensity of the white line during the spin crossover are a consequence of the local order change around the absorbing atom. This change encompasses a shortening of the Fe–ligand distances in the iron coordination shell leading to a modification of the overlap between the *p* atomic orbitals of the ligands and the *p* atomic orbitals with bonding character of the metal, as discussed by Briois *et al.* (1995 ▸). However, even though nearly pure spectra appear to have been obtained by merging all the pixels of both images (spectra which could in principle be used for linear combination fitting necessary for quantification of both spin states in the images), we preferred to perform a multivariate analysis over all the spectra recorded during the steady state and the pressure ramp. In addition to eliminating possible incomplete spin transitions over a domain of the single crystal, the approach allows for the utilization of all the variance contained in the 22368 spectra (Fig. S9 of the supporting information) for isolating true pure spectra by MCR-ALS and obtaining simultaneously the speciation in each pixel. These pure spectra are shown in Fig. 5 ▸(*b*) in comparison with those presented in Fig. 5 ▸(*a*). The largest deviation between pure MCR-ALS spectra and experimental spectra calculated by merging the pixels of images recorded at ambient pressure and 3.40 GPa is found for the LS spectrum, suggesting that an incomplete HS to LS transition has occurred. Obtaining spatially resolved information is then mandatory for confirming such deviation.

**Figure 6:**
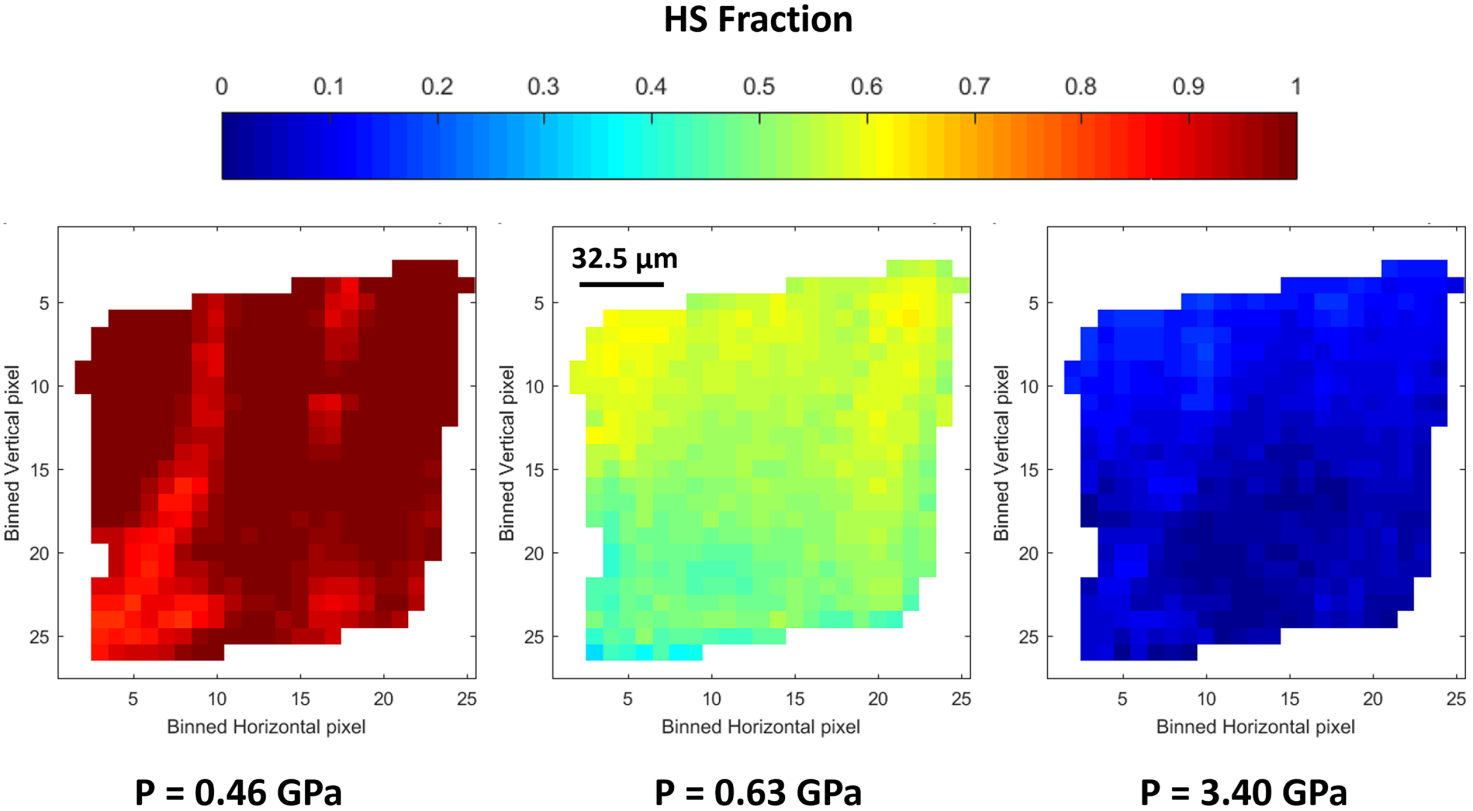
Distribution of the HS state at 0.46, 0.63 and 3.40 GPa quantified by MCR-ALS of the quick-EXAFS spectra measured in each pixel image. The pixel size is 6.5 µm × 6.5 µm. The color scale at the top is proportional to the HS fraction, from deep red = 1 to deep blue = 0.

**Figure 7:**
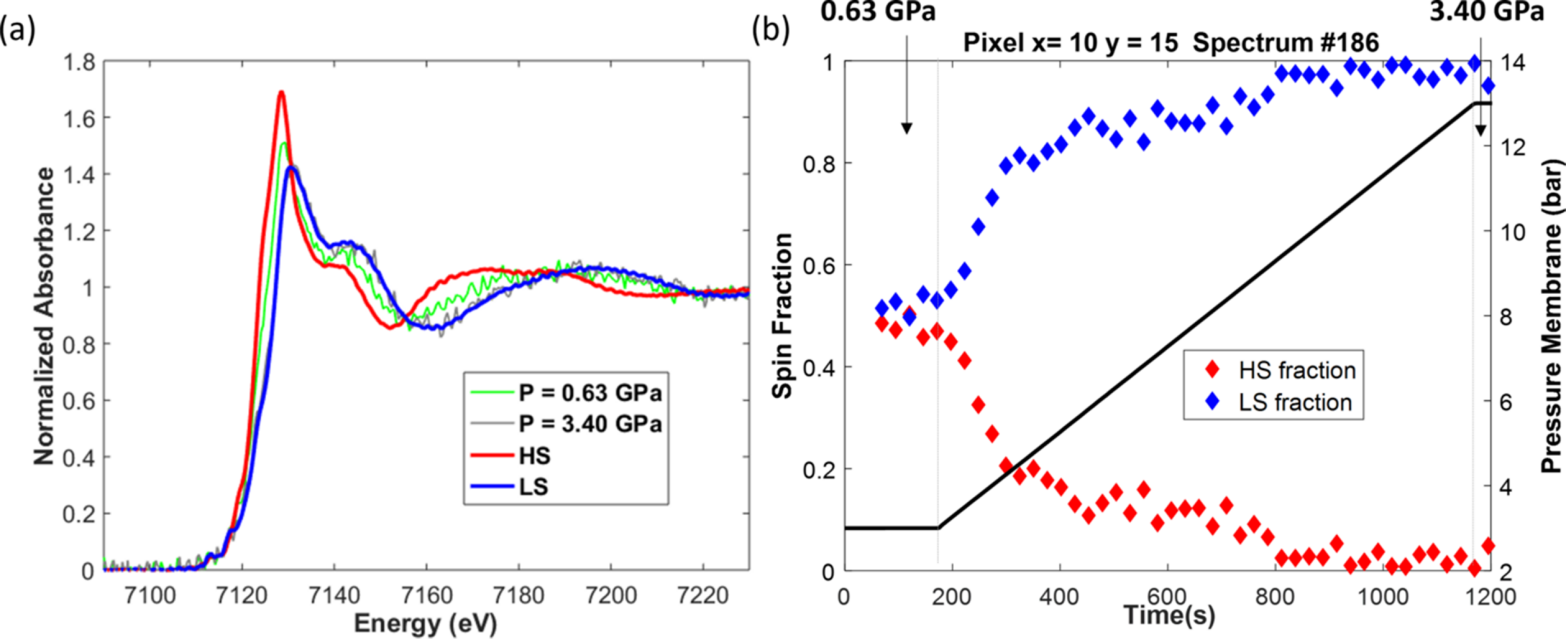
(*a*) Experimental spectra recorded at isobar conditions at pixel *Px* = 10 and *Py* = 15 of images shown in Fig. 6 ▸ for *P* = 0.63 GPa (green noisy spectrum) and 3.40 GPa (gray noisy spectrum) compared with the spectra of HS and LS isolated by multivariate analysis. (*b*) Evolution of the spin fraction at this pixel during an increase of pressure from 0.63 to 3.40 GPa determined by MCR-ALS analysis.

Taking advantage of the measurements of at least ten hyperspectral cubes under isobaric pressure conditions, the distribution of the HS and LS forms in the single crystal, displayed in Fig. 6 ▸, were obtained with a 10 × 10 pixel binning, leading to a spatial resolution of 6.5 µm × 6.5 µm. Examples of linear combination fitting quality at different pixels within the images are presented in Fig. S10 of the supporting information. It is noteworthy from the distribution image at *P* = 0.46 GPa that the transition of the HS form to the LS form initiates from the bottom-left crystal corner and seems to be partially and non-homogeneously propagated in the crystal. The onset of nucleation from a corner of the single crystal does not result from a pressure gradient in the DAC. In fact, it has also been observed for thermally induced spin transitions observed by optical microscopy (Traiche *et al.*, 2017 ▸; Fourati *et al.*, 2019 ▸). In comparison with the iron atoms in the crystal bulk, those at the corner of the single crystal have the largest frustrated coordination characterized by lower metal–ligand bond energy. We assume that such a feature facilitates the motion of atoms for hosting the spin transition from HS to LS which results in important structural modifications at the level of the metal polyhedron, such as the shortening of the metal–ligand bond lengths. As soon as the pressure increases, the LS fraction increases everywhere in the crystal, as expected for a nucleation process. The combination of nucleation and non-homogeneous propagation leads to the coexistence of both spin states with fractions gradually changing with the pressure increase. This corresponds to a non-equilibrium thermodynamic condition inside the crystal, as generally reported for first-order phase transitions with large volume continuity. From this point of view, the spin-crossover process induced under pressure is different from the thermally induced one for which a real propagation front of a single domain from a crystal corner towards the opposite corner is observed by optical microscopy (Traiche *et al.*, 2017 ▸; Fourati *et al.*, 2019 ▸). The dynamics of the propagation for the pressure-induced spin transition presented herein, monitored from 0.63 GPa to 3.40 GPa with a spatial resolution of 9.1 µm (14 × 14 pixel binning) (see the movies of the supporting information), reveal a slight propagation from the bottom to the upper edges with a trend towards the corner opposite to the nucleation center observed at 0.46 GPa but always with the presence of both spin-state forms. The transformation takes place in less than 10 min, as displayed in Fig. 7 ▸, for a pixel located at *Px* = 10 and *Py* = 15 of the speciation map shown in the movies. This corresponds to an increase of the pressure membrane from 3 bar to 9 bar. This unprecedented observation clearly demonstrates how powerful the hyperspectral quick-EXAFS imaging is for monitoring the spatio-temporal dynamics of pressure-induced spin transitions and paves the way for exciting experiments offering experimental evidence for consolidating theoretical models describing the propagation anisotropy of spin domains.

### 3.2. Operando cycling of LiFePO_4_ battery composite electrodes

LFP is an archetype cathode material, for which the heterogeneity of the biphasic transition during lithium intercalation or de-intercalation (discharge/charge reaction) upon battery cycling has been demonstrated by many spatially resolved characterization techniques (Leriche *et al.*, 2010 ▸; Ouvrard *et al.*, 2013 ▸; Strobridge *et al.*, 2015 ▸), 2D imaging techniques (Liu *et al.*, 2010 ▸; Ouvrard *et al.*, 2013 ▸; Arai *et al.*, 2021 ▸) and 3D imaging techniques (Liu *et al.*, 2019 ▸). At the scale of the millimetre-sized electrode device but also of the micrometre- or nanometre-sized active particle, under *operando* or *in situ* conditions, it has been shown that some regions of the electrode and of the active particle can be over-charged and others over-discharged in relation to the expected voltage composition (Liu *et al.*, 2010 ▸, 2019 ▸; Meirer *et al.*, 2011 ▸; Ouvrard *et al.*, 2013 ▸; Nakamura *et al.*, 2014 ▸; Arai *et al.*, 2021 ▸). The discrepancy is assigned to the intrinsic complexity in the composite electrode made of active LFP particles, electrolyte and graphite particles: the connectivity between them and the resulting tortuosity in the composite affect the Li diffusion in the electrolyte and electronic conductivity through the cathode, notably depending on the charge rate conditions (Wang *et al.*, 2014 ▸; Strobridge *et al.*, 2015 ▸). In the second case study presented in this paper, we will first discuss how quick-EXAFS hyperspectral FF imaging can be used for mapping the heterogeneity of the electrochemical reaction at the micrometre scale with second time resolution. Beyond the heterogeneity characterization which has so often been reported in the literature as mentioned above, we will discuss how hyperspectral XAS imaging could be efficiently used for drawing correct conclusions from *operando* monitoring of charge cycling in comparison with non-spatially resolved XAS measurements.

**Figure 8:**
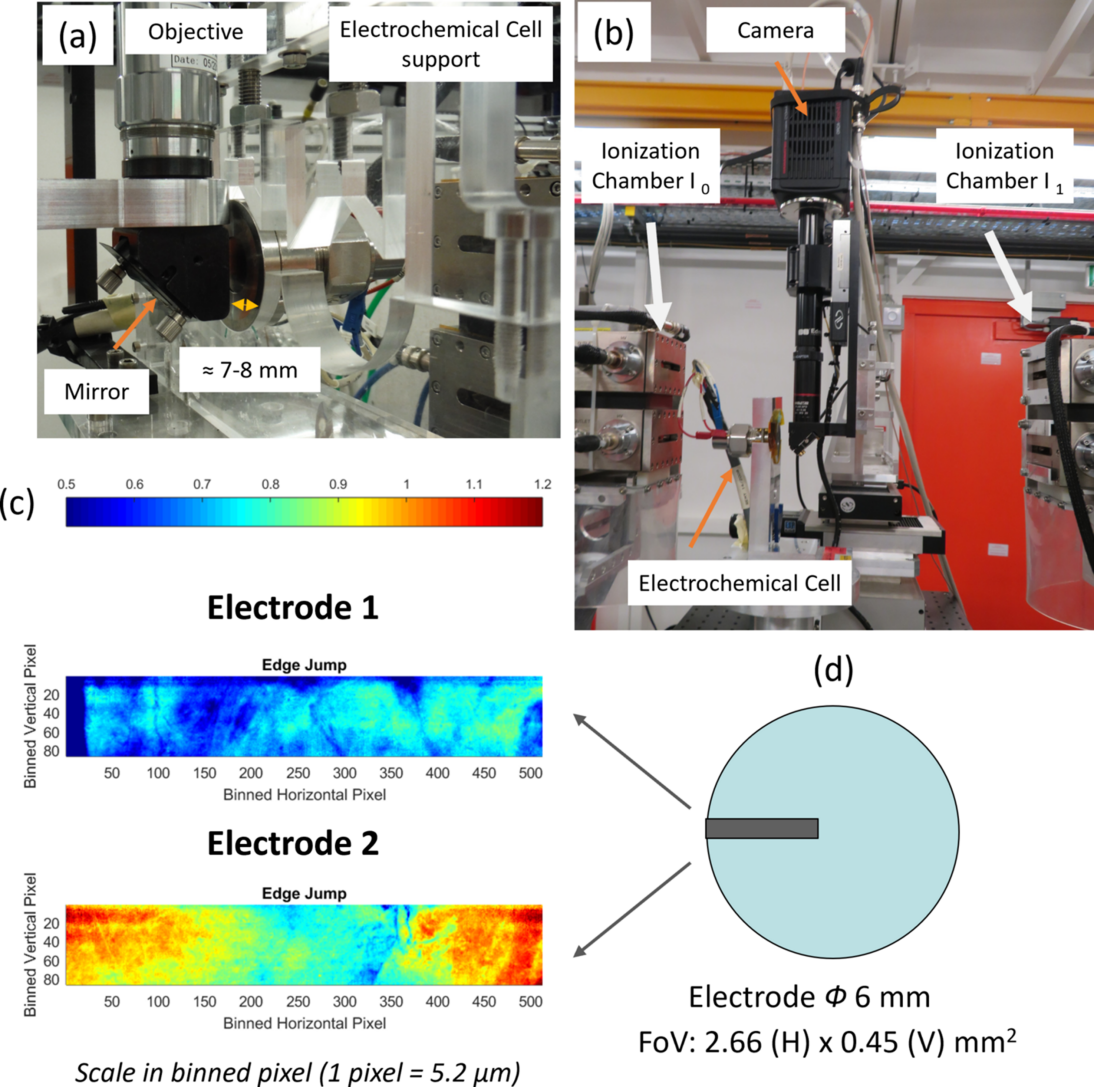
(*a*, *b*) Images of the electrochemical cell in position between the two ionization chambers and in front of the camera optics. (*c*) Iron distribution determined from the XAS edge jump measured at each binned pixel of the hyperspectral images for the two LFP electrodes investigated in the case study. A common color scale of the edge jump for both electrodes is given at the top. (*d*) Schematic of the FoV area (gray rectangle) over the area of the electrode (blue disk of diameter 6 mm).

The set-up used for monitoring the LFP charge by hyperspectral imaging is shown in Figs. 8 ▸(*a*) and 8 ▸(*b*). Although the Leriche-type electrochemical cell (Leriche *et al.*, 2010 ▸) can host a smaller electrode active surface than 6 mm in diameter, matching the largest FoV on the camera available at the beamline (3.3 mm × 2.0 mm), we have intentionally used a FoV (∼2.6 mm × 0.5 mm) sampling only 1/20 of the active electrode surface [Fig. 8 ▸(*d*)] for which the deintercalation rate curves are reported in Figs. S11 and S12 (insofar as such a beam size is representative of one conventionally used for *operando* standard quick-EXAFS monitoring of charge/discharge in electrode materials). The charge of two LFP electrodes presenting a different homogeneity of the iron spatial distribution was studied [Fig. 8 ▸(*c*)]. Electrode 1 is characterized by a nearly homogeneous edge jump map over the FoV as opposed to a non-homogeneous iron distribution for electrode 2 due to variation in the electrode thickness.

**Figure 9:**
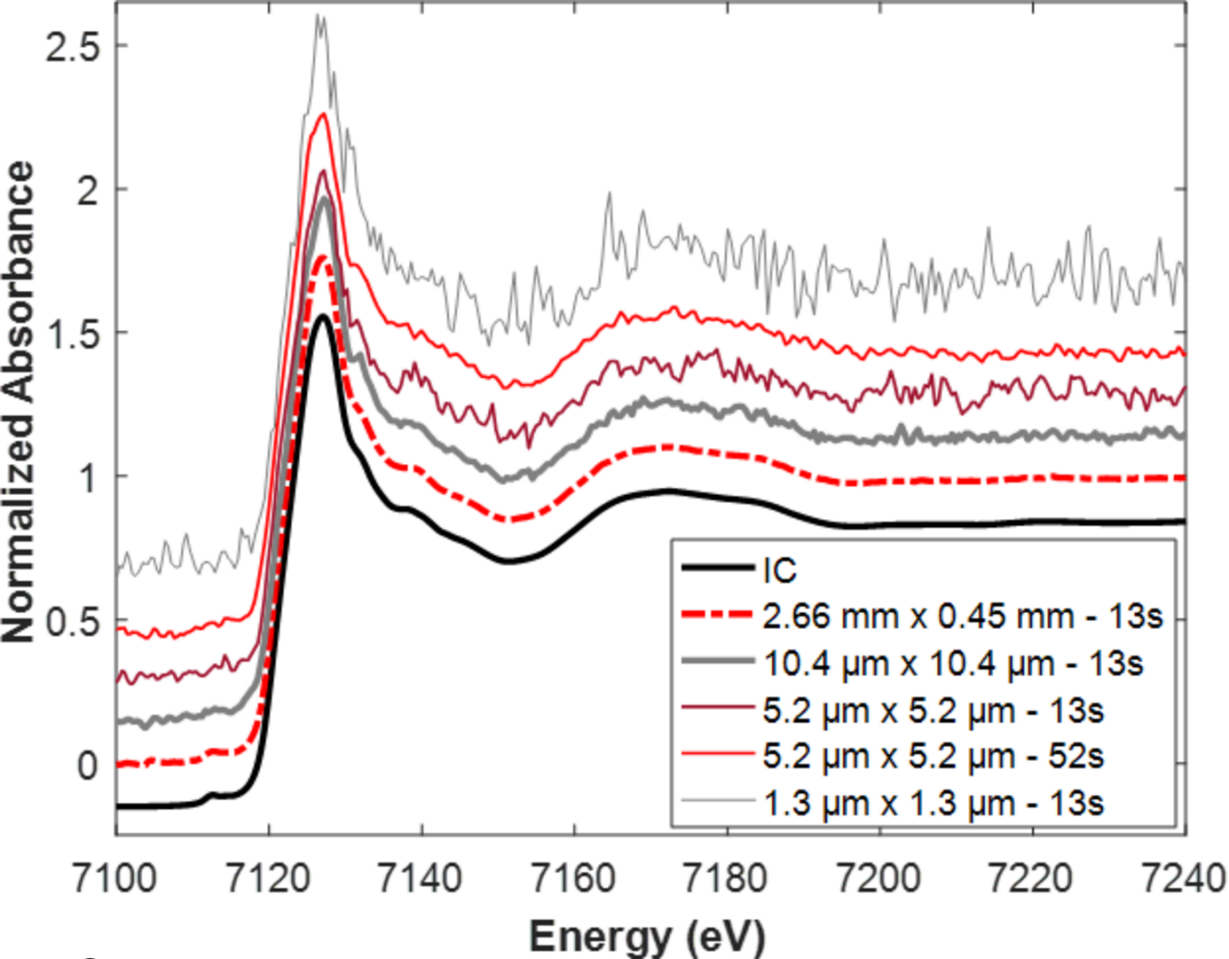
Comparison of the XANES spectrum of the LFP pristine electrode measured using an ionization chamber (IC) and extracted from hyperspectral imaging varying the pixel binning and the cube merging.

Standard quick-EXAFS acquisitions are very helpful during beam time for measuring the global composition of the sample at any stage of the reaction and for making a decision on whether to follow up the hyperspectral monitoring, considering the ionization chamber outcome. Even if the post-treatment process of raw cubes was made easier with the *Jupyter* notebooks developed at the beamline, the time required for raw hyperspectral cube transfer from the computer linked to the ORCA camera to the SOLEIL data storage and subsequent time for post-treatment of hyperspectral images do not allow a quick decision to be made during the experiment. In addition, the measurements of some steady-state conditions, using the ionization chambers or the camera, offer good opportunities to evaluate the quality of the hyperspectral spectra and to find the best compromise for improving the S/N of spectra with pixel binning and cube merging. Fig. 9 ▸ compares for the pristine electrode the spectra recorded using the ionization chamber, using all the pixels of the FoV and using different binning or cube merging strategies. It was decided to proceed with the analysis of the data recorded during the charge monitoring with a spatial resolution equal to the intrinsic spatial resolution of the beamline (Table 1 ▸) considering a 4 × 4 pixel binning (leading to 5.2 µm × 5.2 µm as spatial resolution). To keep a manageable number of spectra to deal with, a merging of four consecutive cubes (leading to 52 s as time resolution), corresponding to the deintercalation of 0.0144 Li by merged cube, was carried out, leading to 85 and 95 merged cubes as a result of the charge monitoring of electrode 1 and electrode 2, respectively (Figs. S13 and S14).

**Figure 10:**
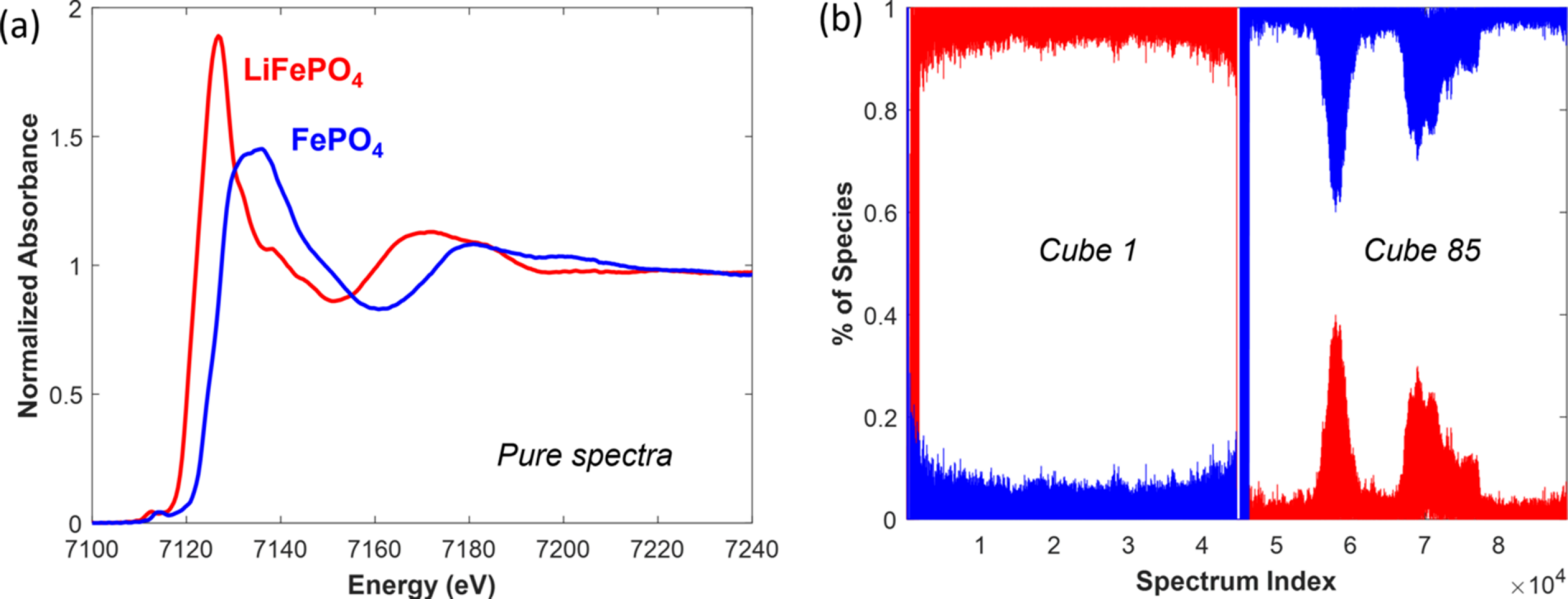
MCR-ALS outcome resulting from the analysis of a dataset built from the flattening of cube 1 and cube 85 recorded during the hyperspectral monitoring of the 1 C charge of electrode 1. (*a*) Pure spectra and (*b*) concentration profile of both species as a function of spectrum index in the dataset.

Considering pixel binning, in each image made of 512 × 87 binned pixels, 44544 spectra were recorded which had to be individually rebuilt by linear combination fitting to extract the chemical speciation of the electrode at a given composition. With the recording of 85 to 95 merged cubes during the 1 C charging of both electrodes, 3786240 to 4231680 spectra were therefore processed using a two-step method. First, the pure spectra corresponding to the delithiated phase and lithiated phase were extracted by MCR-ALS from the variance of the 89088 spectra composing the matrix obtained after unfolding the first and last cubes of the 1 C charge (Figs. S15 to S17). Then the pure spectra, shown in Fig. 10 ▸(*a*) for electrode 1, were imposed in further MCR-ALS minimizations as equality constraints for minimizing each of the remaining cubes measured during the LFP charging. The movies corresponding to the distribution images of LFP and FP species during 1 C charge of both electrodes are given in the supporting information. Examples of linear combination fittings at different pixels within one image for electrode 1, and from which speciation maps shown in the movies are built, are presented in Fig. S18 of the supporting information. It is interesting to note that even for cube 85 measured for electrode 1 the composition extracted by MCR-ALS [Fig. 10 ▸(*b*)] at the end of the charge reveals a partial inhomogeneity in the species distribution with two islands richer in LFP, as shown by the distribution map displayed in Fig. S19.

**Figure 11:**
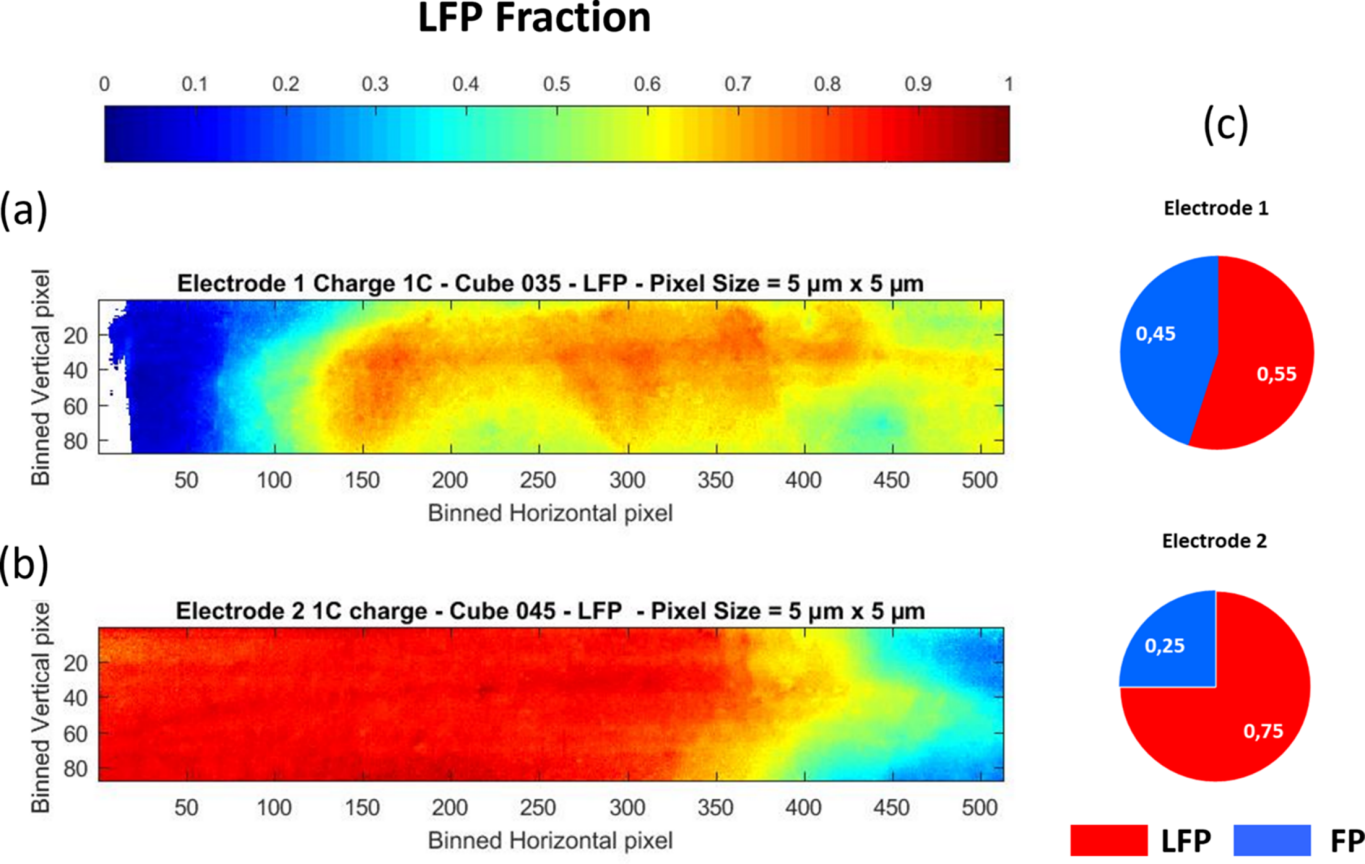
LFP fraction distribution map for (*a*) electrode 1 and (*b*) electrode 2 for a voltage corresponding to 0.5 Li deintercalated during a 1 C charge of electrodes. Spatial resolution 5.2 µm × 5.2 µm. The fractions associated with the color of the colorbar at the top were obtained from the linear combination fitting of the 44544 spectra on each image considering the pure LFP and FP spectra isolated by MCR-ALS (Figs. S16 and S17). (*c*) Global composition for the FoV of both electrodes investigated by hyperspectral imaging considering the spectra averaged over all the pixels.

The movies display the dynamics of the spatial distribution of LFP measured for each electrode during 1 C charge and highlight the heterogeneous behavior of the phase transformations, irrespective of the considered electrodes. Considering, for instance, in Figs. 11 ▸(*a*) and 11 ▸(*b*) the spatial distribution of LFP measured for both electrodes at a voltage corresponding to the extraction of 0.5 Li^+^, local over-charge and over-discharge clearly coexist in the imaged area for which a uniform green color would be expected for homogeneous transformation. The global composition [Fig. 11 ▸(*c*)] determined for electrode 1 from the average of all the spectra in the image appears closer to the targeted composition than that calculated for electrode 2. This results from a better preparation of electrode 1 compared with electrode 2 for which the behavior at the FoV scale is like the charge capacity measured for the entire electrode.

**Figure 12:**
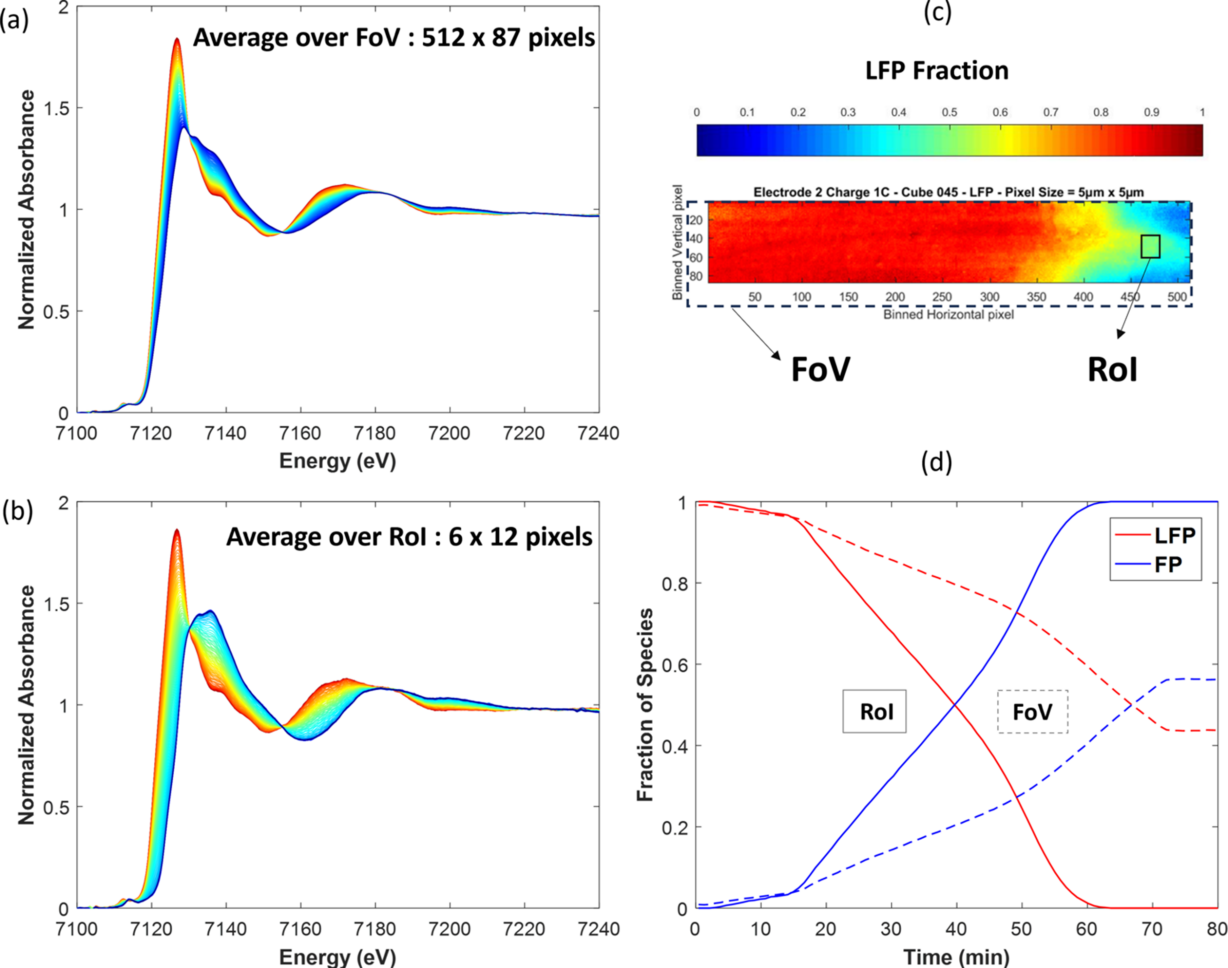
(*a*) Evolution of the spectra averaged over the full FoV during the monitoring of the 1 C charge of electrode 2, mimicking the outcome of quick-EXAFS monitoring without spatial resolution. (*b*) Evolution of the spectra averaged over the RoI displayed in (*c*) during the monitoring of the 1 C charge of electrode 2. (*c*) LFP distribution map at half Li deintercalation obtained at 5.2 µm × 5.2 µm spatial resolution. (*d*) Concentration profiles as a function of time obtained during the monitoring of the 1 C charge of electrode 2 by MCR-ALS of data measured over the FoV in (*a*) – dashed lines – or over the RoI in (*b*) – full lines. Spectra isolated for the RoI as pure spectra of LFP and FP species were used as equality constraints for the MCR-ALS minimization of the dataset recorded over the FoV.

The discrepancy between the global composition deduced from the FoV analysis and targeted composition prompts a general comment about *operando* quick-EXAFS characterization of battery cycling without spatial resolution. Depending on the beam position on the electrode at which characterization has been performed, the discrepancy between both compositions can be important, making it difficult to understand the electrochemical behavior and establish the structure/charge-capacity relationship. This is particularly true if only a single position over the electrode has been measured by quick-EXAFS. As an average measurement, without spatial resolution, the probability of reaching the full charged or discharged state at the end of the electrochemical reaction is very low, which is a strong limitation for correctly deciphering the pure spectra by MCR-ALS and therefore drawing the correct evolution of concentration profiles. For this reason it is highly recommended, when monitoring battery cycling by *operando* standard quick-EXAFS, to explore several positions over the electrode (Eveillard *et al.*, 2020 ▸; Blondeau *et al.*, 2022 ▸) to increase the spectral variance for minimizing the risk of incomplete reaction analysis. Hyperspectral imaging offers a good alternative to the problem of incomplete reactions by the increase of variance gained by the huge increase of spectra measured during cycling. Fig. 12 ▸ reports a comparison of the MCR-ALS analysis carried out over spectra averaged over all the pixels [Fig. 12 ▸(*a*)], mimicking the standard quick-EXAFS monitoring (without spatial resolution) and leading to an average concentration of species for electrode 2, with the MCR-ALS analysis obtained over the selected RoI [Fig. 12 ▸(*b*)] matching the expected rate of Li deintercalation [the green color area in the speciation map corresponding to 0.5 Li deintercalated presented in Fig. 12 ▸(*c*)]. The comparison of concentration profiles for both datasets [Fig. 12 ▸(*d*)] highlights how post-processing of hyperspectral imaging can be efficiently performed for facilitating electrochemistry interpretation and drawing correct conclusions from *operando* experiments.

In addition to RoI selection in the 2D maps for matching the intercalation–deintercalation rate with speciation, hyperspectral FF imaging could be efficiently used to mitigate radiation damage on the dose-sensitive electrode materials or electrolyte (Blondeau *et al.*, 2022 ▸; Jousseaume *et al.*, 2023 ▸). Indeed, the beam size for FF imaging can be much larger than that used for conventional measurements, leading to an effective decrease of photon density by several orders of magnitude and thus to a radiation dose bearable by the sample. Despite a significant S/N decrease of hyperspectral images recorded with such a low photon density, it has been demonstrated by the previous case study, using a very absorbing DAC environment, that MCR-ALS is a powerful method for extracting from noisy spectra accurate redox evolution and high-quality pure spectra with a high S/N ratio.

Although 2D XAS imaging of lithium intercalation or deintercalation in electrode battery materials has been commonly reported in the literature (Liu *et al.*, 2010 ▸; Ouvrard *et al.*, 2013 ▸; Arai *et al.*, 2021 ▸), the second temporal acquisition for FF quick-EXAFS imaging presented herein is also clearly an opportunity for the study of faster charging batteries. Herein, the 1 C monitoring of the battery has been carried out considering a merge of four consecutive cubes to obtain a total number of spectra that is manageable for further MCR-ALS analysis. However, if we consider a 4 C charging of a battery taking place in 15 min to completion, the same number of cubes (∼80) will be recorded with the quality of the data per cube good enough for analysis, as can be concluded from the data reported in Fig. 9 ▸ for the 4 × 4 pixel binning in 13 s. In addition, as usually carried out during the monitoring of slow-charging batteries by conventional quick-EXAFS (Blondeau *et al.*, 2022 ▸; Wernert *et al.*, 2023 ▸), three electrochemical cells can be simultaneously cycled and alternately measured in order to optimize the use of beam time. A similar strategy could be proposed for the alternate 2D imaging of several cells at 1 C or slower charging, provided there is a synchronous exchange of the cells during the descending oscillation of the monochromator where no images are acquired.

### 3.3. Monitoring the activation of a bimetallic catalyst

Along catalytic beds, gradients in the reactant concentration and in the temperature are well known factors affecting the catalyst speciation, and thus the local catalytic performance. This makes spatiotemporally resolved characterizations mandatory to properly derive the electronic and structural information in relation to the activity/selectivity of catalysts. Among the techniques offering such information, spatially resolved synchrotron radiation methods have received tremendous interest over the last few decades, as reviewed in many publications (Grunwaldt *et al.*, 2009 ▸; Urakawa & Baiker, 2009 ▸; Andrews & Weckhuysen, 2013 ▸; Bañares & Daturi, 2023 ▸). Imaging methods based on X-ray fluorescence are particularly powerful for unraveling the co-location of several elements of interest in catalytic grains (Price *et al.*, 2015 ▸; Sheppard *et al.*, 2017 ▸), but, when addressing chemical and local order spatial heterogeneity on a catalytic bed by hyperspectral XAS imaging, the characterization is rarely multi-element (Grunwaldt *et al.*, 2006 ▸; Hannemann *et al.*, 2007 ▸), at least not during the same experiment. The multi-edge characterization of multi-element catalysts available at the ROCK quick-EXAFS beamline has been shown to be very attractive for shedding light on synergetic relationships taking place during a single reaction (Lesage *et al.*, 2019 ▸; Kokliukhin *et al.*, 2021 ▸; Klag *et al.*, 2023 ▸; Robert *et al.*, 2023 ▸). Such potentiality has been extended to the imaging capability implemented at the beamline, as recently reported for NiCu bimetallic catalysts (Briois *et al.*, 2024 ▸) for which a stack of 580 absorption images were recorded over an energy range of 1000 eV. In the third case study presented herein, we extend the energy range to more than 2000 eV with multi-edge FF hyperspectral quick-EXAFS imaging monitoring of the activation of a bimetallic SiO_2_-supported catalyst containing the same amount of Fe and Cu. To the best of our knowledge, this is the first time that such simultaneous imaging performed at the Fe and Cu *K*-edges has been reported.

**Figure 13:**
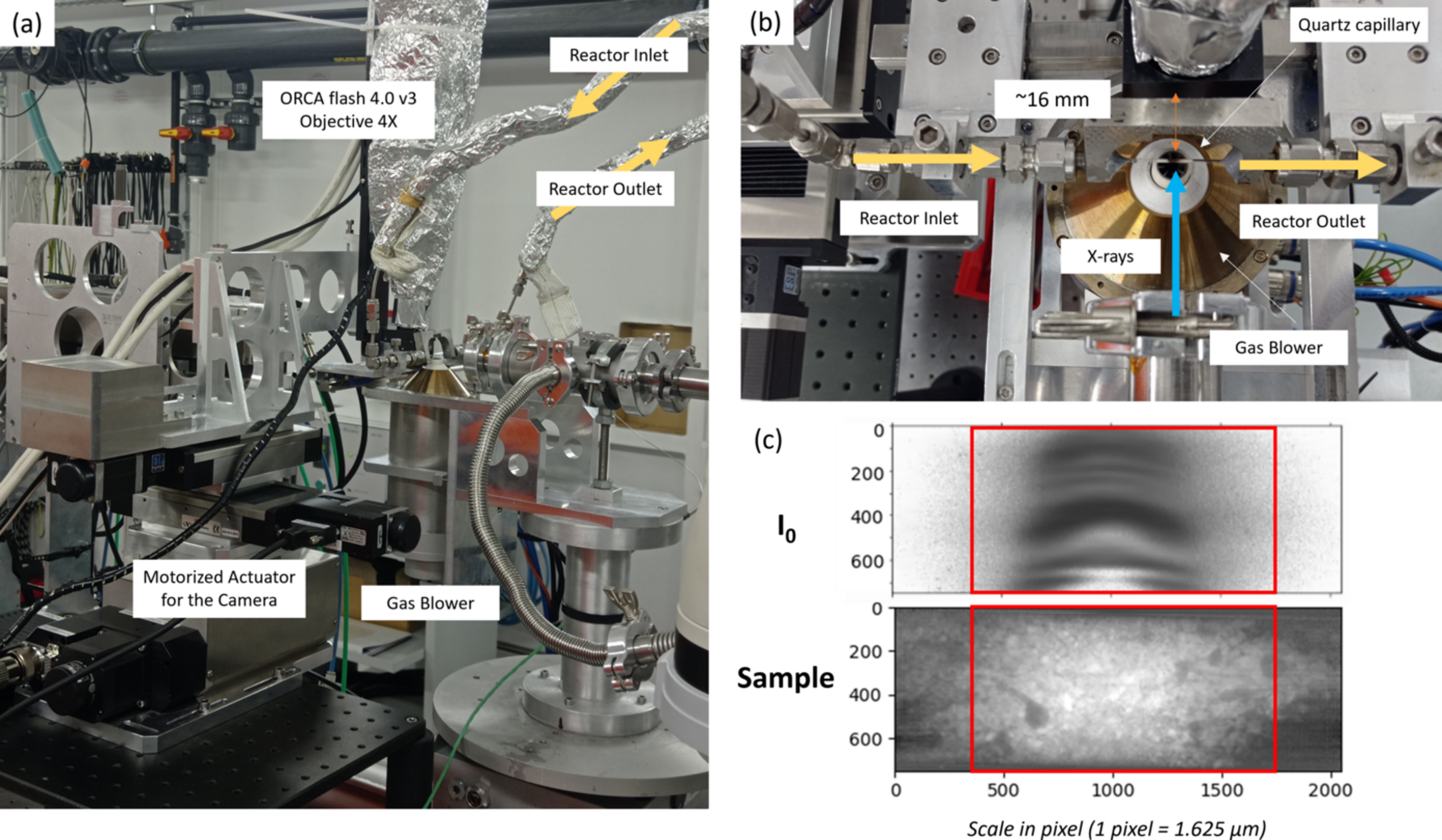
(*a*, *b*) Images of the setup showing the gas blower and quartz capillary reactor containing the catalyst bed. The distance between the capillary and the reactor was set to 16 mm. At this distance the temperature of the scintillator is around 40°C for a setpoint temperature of the gas blower of 560°C (temperature at the sample position 500°C). (*c*) *I*_0_ flat-field image (top) and absorption contrast image (bottom) of the catalyst bed at room temperature obtained by dividing the intensity measured at a given energy after the edge energy by the intensity measured at a pre-edge energy. The RoI of the images considered for data analysis is displayed by the red rectangle: its size is 1400 (H) × 740 (V) pixels.

The setup used for this study is displayed in Fig. 13 ▸. The FWHM beam size at ∼2.5 m upward of the focusing optic position was 1.5 mm (H) × 1.2 mm (V). With a FoV on the sensor made of 2048 (H) × 748 (V) pixels, *i.e.* 3.3 mm × 1.2 mm, the RoI in the images used for data post-treatment was set to 1400 pixels (H) × 740 (V) [red delimited area in Fig. 13 ▸(*c*)], *i.e.* 2.3 mm (H) × 1.2 mm (V), meaning that the tails of the diffuse beam were used in the data processing of the images in order to expand to a larger scale the analysis of heterogeneity and speciation along the catalyst bed.

**Figure 14:**
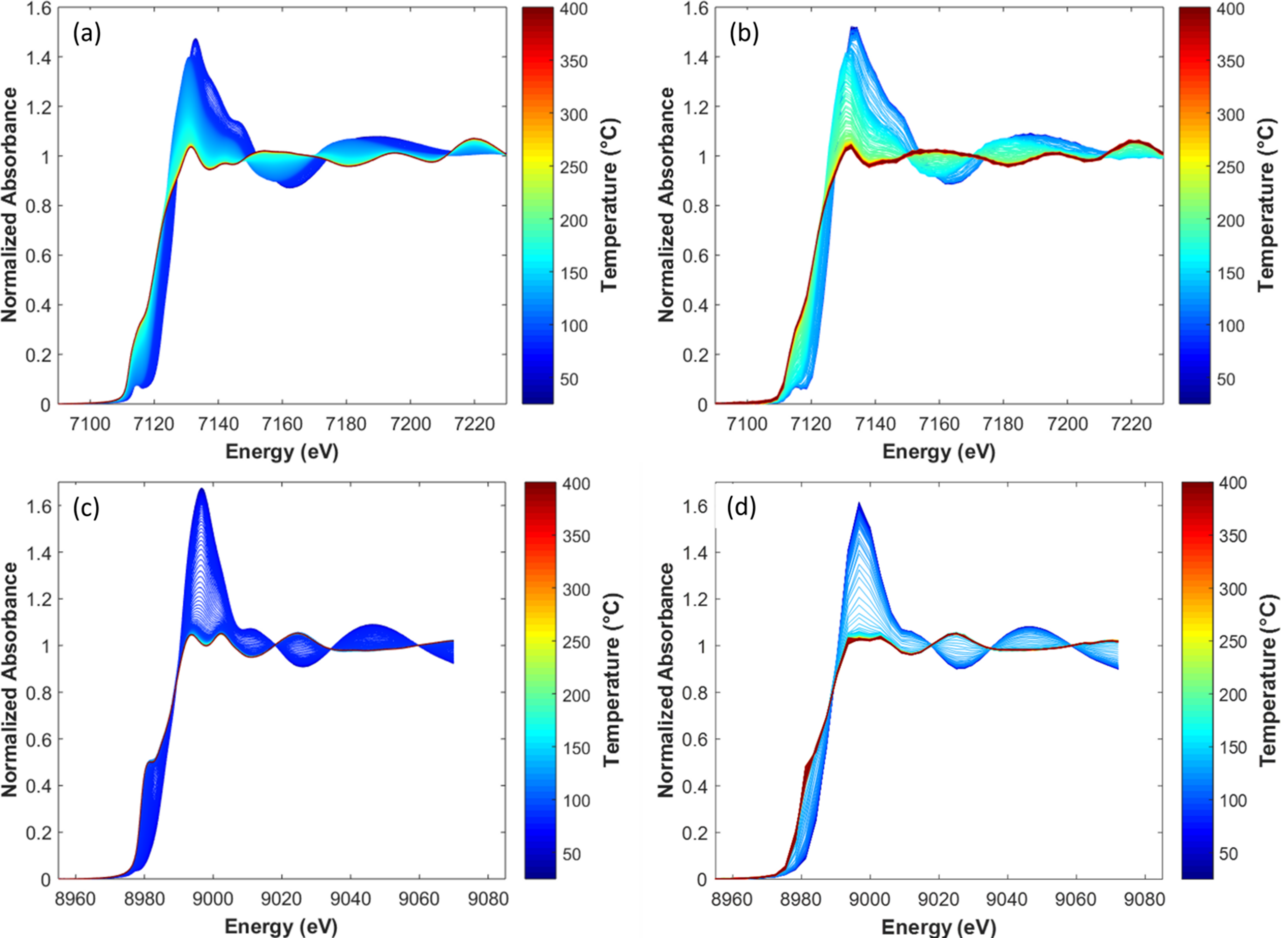
Fe *K*-edge and Cu *K*-edge XAS monitoring of the reductive activation of the FeCu catalyst carried out by heating the catalyst from 25 to 400°C followed by one hour of isothermal heating at 400°C. Panels (*a*) and (*c*) show quick-EXAFS (1176 spectra, each being the average of ten spectra measured at the acquisition rate of 250 ms). Panels (*b*) and (*d*) show FF hyperspectral XAS imaging (300 spectra averaged over all the pixels of the catalyst bed and recorded each in 5.56 s).

To evaluate the quality of the spectra recovered by FF hyperspectral XAS imaging, which is intrinsically based on separate measurements of the sample absorption and of the *I*_0_ flat-field – both measurements being performed with a time interval of several tens of minutes or even 2 or 3 h – we first compare in Fig. 14 ▸ the Fe and Cu *K*-edge dataset recorded with the high time resolution of the quick-EXAFS mode (one spectrum every 250 ms, ten spectra merged in 5 s) and the average of the spectra recovered by FF imaging over all the pixels of the FeCu/SiO_2_ catalyst bed (one spectrum recorded in 5.56 s but every 11.1 s). The energy distribution of spectra derived from hyperspectral cubes presented in Figs. 14 ▸(*b*) and 14(*d*) is sparser than those measured by quick-EXAFS, with 370 energy values at the Fe *K*-edge and 78 at the Cu *K*-edge for the imaging dataset against three to seven times more energy points for quick-EXAFS spectra, respectively. Despite a poorer time and energy resolution for FF imaging derived spectra, the similarity of the outcomes (Figs. S22 to S26) obtained by MCR-ALS analysis for both datasets not only proves the high stability of the beamline for offering spatially resolved spectra with high data quality but also magnifies the strength of the beamline for the monitoring of dynamic mass transport phenomena with characteristic time scales of hundreds of milliseconds (Kalz *et al.*, 2017 ▸). Strict interchangeability of the spectral information recorded by quick-EXAFS is observed with that obtained by FF hyperspectral imaging, provided that the former is projected on the 580 energy values corresponding to the recording of images in the hyperspectral cube. Therefore, the MCR-ALS spectra of pure species derived from the quick-EXAFS dataset projected over the energy grid of the imaging can be used, if necessary, as an initial guess (even as constraints) in the minimization of the imaging dataset.

**Figure 15:**
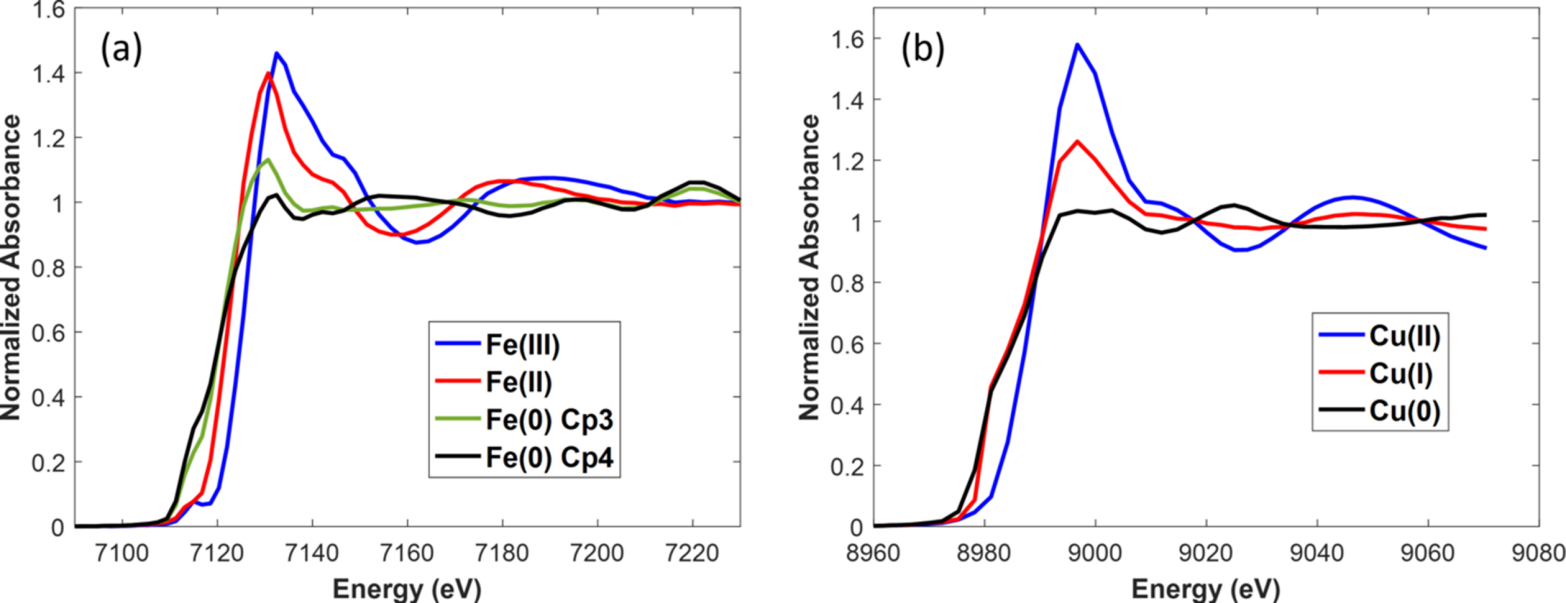
(*a*) Fe *K*-edge and (*b*) Cu *K*-edge pure spectra isolated by MCR-ALS during the FF hyperspectral XAS imaging monitoring of the activation of the FeCu/SiO_2_ catalyst presented in Figs. 14 ▸(*b*) and 14(*d*).

This first analysis carried out both on the quick-EXAFS dataset and on the spectra dataset obtained by averaging all the pixels from each imaging hyperspectral cube recorded for the catalyst bed (Figs. S25 and S26) reveals that the activation is explained by the succession of four Fe species [initial Fe(III), Fe(II) intermediate and two Fe(0)-containing species, labelled hereafter Fe(0)-Cp3 and Fe(0)-Cp4, respectively (Cp3 and Cp4 = MCR-ALS components sorted out by their sequence of appearance)] and three Cu species [initial Cu(II), Cu(I) intermediate and Cu(0)], respectively. The spectra isolated from the average of the pixelated spectra are presented in Fig. 15 ▸. The identification of those species is beyond the scope of the paper and will be discussed in a forthcoming publication in relation to environmental scanning transmission electron microscopy carried out during activation on the same FeCu/SiO_2_ catalyst.

**Figure 16:**
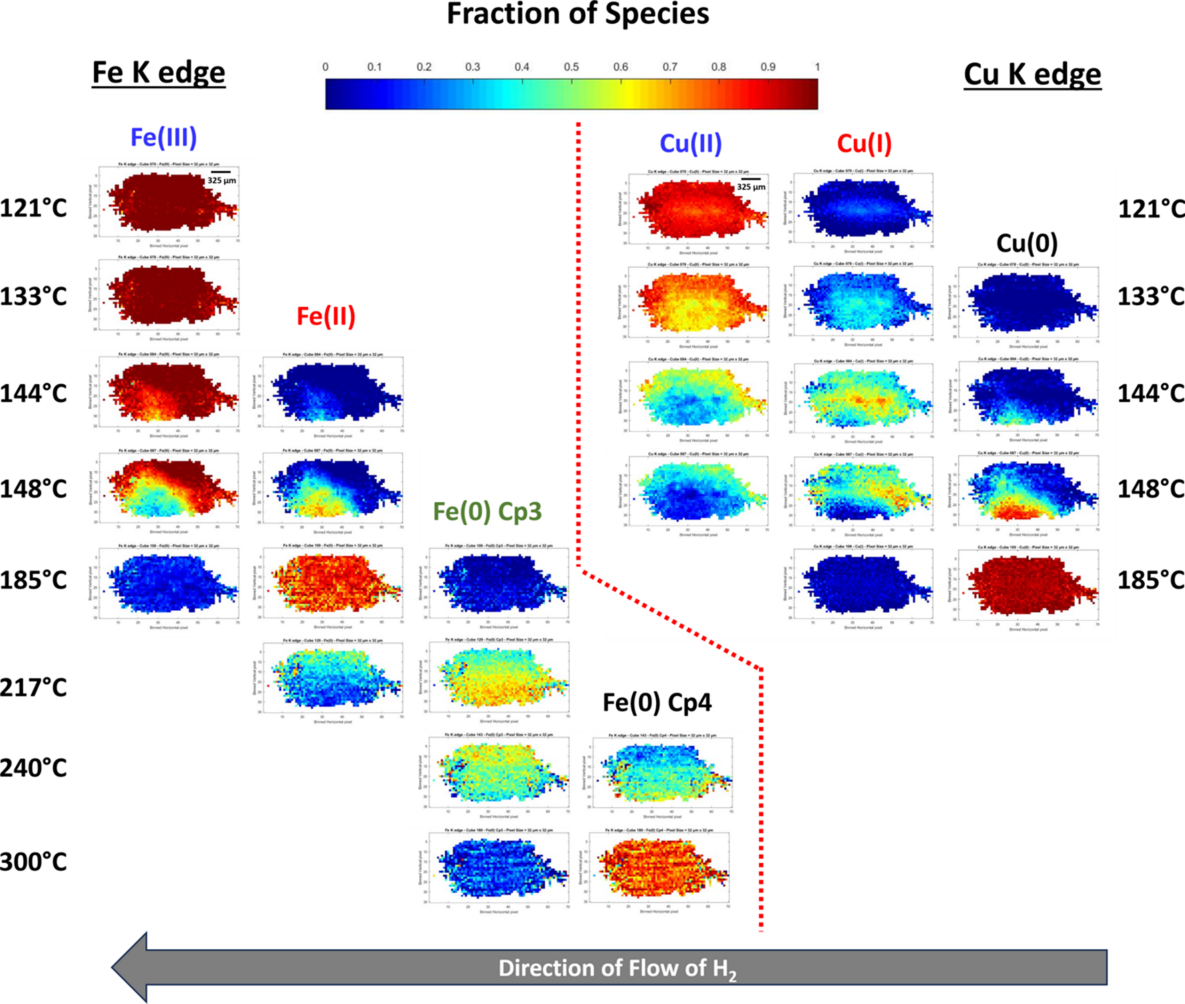
Speciation maps at the Fe *K*-edge and Cu *K*-edge derived from MCR-ALS minimization of the hyperspectral imaging dataset (180 cubes) measured during the monitoring of the activation of the FeCu/SiO_2_ catalyst under H_2_ for selected temperatures. The pixel size is 32.5 µm × 32.5 µm. The color bar is related to the fraction of species. The heater is located at the bottom of the image. The flow of hydrogen is from the right to the left of the capillary.

A 20 × 20 pixel binning, leading to a binned pixel size of 32.5 µm by 32.5 µm, has been considered as a good compromise for keeping the time resolution during the activation monitoring and for offering meaningful spatially resolved information over the catalyst bed. Details on the MCR-ALS minimization performed on the dataset gathering the unfolded spectra of each cube are given in the supporting information. The distribution maps over the catalyst bed for some selected temperatures are displayed in Fig. 16 ▸. Examples of linear combination fittings at different pixels within the images, from which speciation maps shown in Fig. 16 ▸ are built, are presented in Figs. S29 and S30 of the supporting information. First it is clearly observed that the reduction of species begins in the zone of the reactor which is closer to the gas blower nozzle and progresses radially with a cone shape according to the unidirectionality of the air blower heating (from the bottom of the image to the top), possibly leading to a radial temperature gradient. Actually, as this experiment was designed to demonstrate the efficiency of hyperspectral imaging carried out at two edges for shedding light on spatially correlated transformations of Fe and Cu species along the catalyst bed, no special care was taken to minimize any radial temperature gradients, as could be done by using a Kapton hood (Newton *et al.*, 2019 ▸). As presented in Fig. 16 ▸ and in the movies provided as supporting information, for copper, reduction first takes place at around 133°C from Cu(II) to Cu(I) without a change of the oxidation state of the trivalent iron species. The distribution map clearly shows that the onset of formation of Fe(II) species at 144°C is concomitant with the reduction of Cu(I) into Cu(0), occurring at the closest position of the gas blower nozzle assumed to be the hotter zone, and rapidly spreading in the air-blowing cone. Note that, despite the high heating rate of 10°C min^−1^, the dynamics of copper and iron reduction across the catalyst bed can be followed inside a temperature interval of only a few degrees. Copper reduction is complete at ∼160°C. Then, in a second stage, Fe(II) first transforms into Fe(0)-Cp3 with the dynamics of the transformation still taking place from the side of the gas blower nozzle but with a homogeneous radial spreading from the bottom to the top. The same behavior occurs for the transformation of Fe(0)-Cp3 into Fe(0)-Cp4, and the reduction of iron is nearly complete at the start of isothermal heating at 400°C.

**Figure 17:**
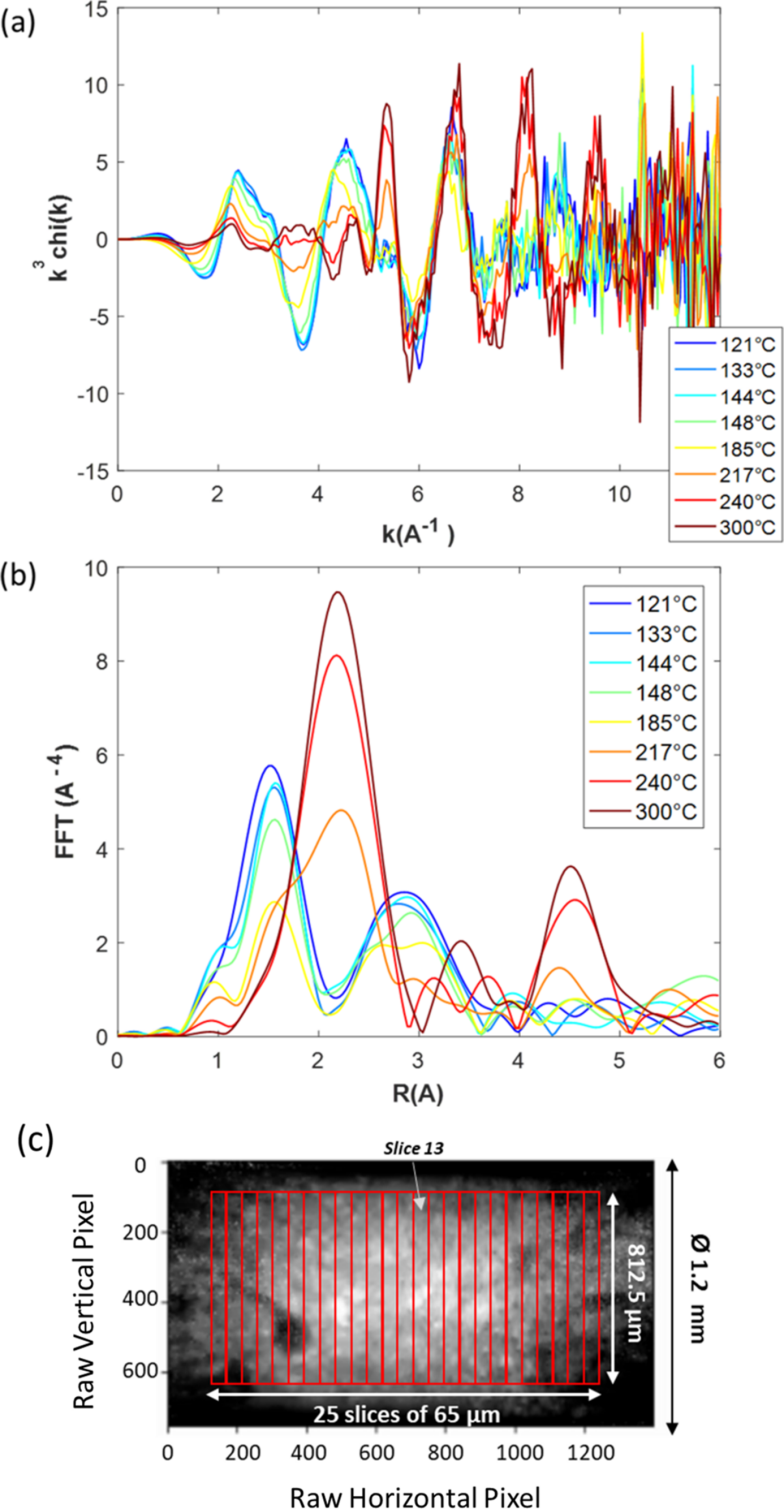
(*a*) EXAFS spectra at the Fe *K*-edge and (*b*) associated Fourier transform moduli extracted from a pixel at the center of the catalyst bed. The extraction of EXAFS spectra has been performed on a RoI with a single vertical pixel of size equal to 500 raw pixels (812.5 µm), for covering the inner radial section of a capillary of 1.2 mm diameter, and 25 slices, each being equal to 40 raw pixels (65 µm), for slicing axially the catalyst bed in equal sections, as displayed in (*c*). The EXAFS spectra were reported at pixel *Px* = 13, *i.e.* just above the gas blower nozzle. A single cube measured in 11.1 s has been used for EXAFS extraction. The cubes are related to the temperatures for which speciation maps are presented in Fig. 16 ▸. EXAFS and Fourier extraction parameters are reported in Table S3.

It is noteworthy that the Fe *K*-edge plots in Figs. 14 ▸(*a*) and 14(*b*) are centered on the XANES region for sake of comparison but an extended energy range has been collected for the same cubes which allows EXAFS and Fourier transforms of EXAFS spectra to be extracted, as presented in Fig. 17 ▸ for the selected temperatures shown in Fig. 16 ▸. As intrinsically the S/N for the EXAFS signal is lower than that obtained on the XANES part, larger binned pixels were considered with slices of 65 µm equally distributed along the catalyst bed across the diameter of the capillary (812.5 µm). This corresponds to the binning of 20000 raw pixels recorded in a single cube made of 500000 raw pixels.

## 4. Discussion

Worldwide, synchrotron radiation facilities are currently moving towards fourth-generation light sources (Chapman, 2023 ▸) for which the beam will be exceedingly bright and coherent. If the benefits of the upgraded machines for imaging and tomography techniques are clear, those for conventional X-ray absorption beamlines are more debatable. No significant increase of flux at those beamlines is expected, which could be beneficial for instance for increasing the fluorescence detection limit or speed acquisition, but rather a major increase of photon density on the sample as a result of the brighter source will be obtained, which is extremely prejudicial for beam-sensitive samples. In the perspective of SOLEIL’s upgrade (Nadji & Nadolski, 2023 ▸; Susini *et al.*, 2024 ▸), the ROCK beamline started to diversify its portfolio of XAS-related techniques, combining the high time-resolution offered by the quick-EXAFS mode with micrometre-scale FF imaging. Such a development enables deeper insight on the dynamic transformation of heterogeneous systems of interest today at the beamline, *i.e.* battery electrode materials and catalysts in working conditions (La Fontaine *et al.*, 2020 ▸). With the same imaging method, we have also explored the heterogeneous nucleation and propagation of spin states within a spin crossover single crystal, as presented herein. More recently, the Mo uptake of alumina extrudates during impregnation/maturation by Mo-based oxidic precursor solutions together with transformation kinetics during subsequent drying (Barata *et al.*, 2023 ▸) have been investigated. The interactions between As and natural organic matter in flowing conditions mimicking the transfer of pollutants in environmental systems (Hoffmann *et al.*, 2012 ▸) has also been explored. Those studies will be presented in forthcoming papers. The broad scientific cases for which quick-EXAFS FF hyperspectral imaging have been used at ROCK have exemplified how relevant and powerful the method is. With the promises of the benefits of fourth-generation light sources for imaging techniques releasing the constraints for the distance between sample and pixelated-array camera for optimal spatial resolution, more space for the installation of bulky environmental setups will be available, offering good conditions for *in situ* and *operando* characterizations of functional materials. In addition, as already emphasized with the battery electrode case study, the versatile defocused beam size in both directions used at ROCK for quick-EXAFS FF imaging will be very useful for mitigating beam radiation damage expected with fourth-generation synchrotron radiation facilities as a negative facet of the brighter source beneficial for speeding up the acquisition rate for imaging techniques. We can surmise that FF quick-EXAFS imaging activity at ROCK will increase a lot in the future and perhaps will overcome the demand of conventional quick-EXAFS measurements at fourth-generation light sources provided that faster readout cameras are available for increasing the number of energy values per spectrum and for achieving a better time resolution per cube. However, such a fate of FF XAS imaging at ROCK not only requires the development of data acquisition methodologies encompassing the variability of samples, FoV and spatial resolution as presented in the paper but also the availability of robust skills for data processing, analysis and storage.

Home-made *Jupyter* notebooks have been developed at the beamline for data processing which are efficient for pre­visualizing the data and making decisions about the pixel binning to use for having a good S/N ratio. In the scope of studying the dynamic development of spatial heterogeneity, we have showcased with the three science cases how intrinsically time and space resolution of the information provided by hyperspectral FF Quick-XAS imaging are related. It is worth mentioning that the cubes processed as inputs by the home-made *Jupyter* notebooks result from no-pixel-binned cubes, meaning that the pixel size afforded by the sensor after objective magnification is retained (Table 1 ▸) at this stage of data processing. Pixel binning, in compliance with the intrinsic spatial resolution taking account of the beamline source, optics and distance between camera and sample (Table 1 ▸), is performed as a second data processing step as a trade-off between spatial resolution and time resolution required for a good description of the chemical transformation.

For steady-state conditions, as no evolution occurs with time, several tens or hundreds of cubes can be merged to increase imaging statistics before normalizing spectra extracted after a moderate pixel binning. Ideally, the moderate pixel binning should be of the same order of magnitude as the intrinsic spatial resolution (Table 1 ▸) which has been roughly evaluated to be about four times the pixel sensor size after magnification. It is important to consider sample absorption for evaluating the number of cubes to be merged for displaying images at the intrinsic spatial resolution. This is, for instance, exemplified with electrode battery materials for which a good S/N ratio for Fe *K*-edge XANES spectra extracted with pixel binning at intrinsic spatial resolution (5.2 µm × 5.2 µm) is achieved with a merge of four cubes (Fig. 9 ▸) instead of 40 cubes for the Fe spin crossover complex measured at isobaric conditions in the DAC with 1% of transmission by the diamonds (Fig. S31).

The strategy used for finding the trade-off between spatial resolution and time resolution for monitoring a dynamic transformation shall include the time resolution that we want to keep for describing the process: this will give per raw pixel the number of spectra, *n*_s_ (*n*_s_ = 1 for no cube merging as for the case study 3, *n*_s_ = 4 for a merge of four cubes used for case studies 1 and 2). This value is then multiplied by 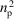 considering a *n*_p_ × *n*_p_ pixel binning. To properly evaluate the pixel binning, we usually start with a dataset corresponding to a steady-state condition for which, as already mentioned, a substantial number of cubes, *N*_c_, has been collected and a pixel binning close to the intrinsic spatial resolution for the sample performed, *n*_isr_. If the S/N ratio for the so-obtained steady-state spectra is suitable for further data analysis, 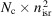 is the benchmark value of merged spectra to reach for the dynamic study, by applying 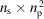. This is, for example, illustrated by monitoring the spin transition single crystal. A satisfactory S/N ratio is obtained for 40 steady-state cubes merged with a 4 × 4 pixel binning leading to 640 spectra (Fig. S31); similar data quality can be therefore achieved with *n*_s_ = 1 and *n*_p_ = 26 with a total of 676 spectra, or slightly better S/N ratio with *n*_s_ = 4 and *n*_p_ = 14 with a total of 784 spectra. According to the slow pressure increase of the DAC membrane and for keeping a significative spatial resolution in comparison with the crystal size, the second pair of values (*n*_s_ = 4 and *n*_p_ = 14) was considered as the best compromise for a good description of the spatial heterogeneity during the spin transition monitoring shown in Fig. 7 ▸(*b*).

Another point to consider during the evaluation of *n*_s_ and *n*_p_ is the huge amount of data generated per hyperspectral cube in relation to the computation time for further processing of the unfolded cubes by MCR-ALS minimization. For instance, if *n*_s_ = 1 and *n*_p_ = 20 has been considered a good compromise for case study 3, this choice was imposed more by the number of pixels to deal with for further MCR-ALS minimization of the 180 cubes rather than the S/N ratio, for which *n*_s_ = 1 and *n*_p_ = 4 would probably be good enough (Fig. S32). Namely, we have demonstrated with the three case studies that the use of the multivariate MCR-ALS analysis for data analysis appears as a robust method for eradicating random noise and extracting noise-free pure spectra. This results from the fact that the pure spectrum of a species is rebuilt from its contribution in each spectrum of the dataset. The huge number of pixels in each dataset is herein a clear advantage for eradicating noisy data. But, for the former binning (*n*_s_ = 1 and *n*_p_ = 20), the full data recorded for case study 3 with 180 cubes would be made of 441000 spectra which are still manageable on a personal microcomputer (Fig. S27 in the supporting information) as opposed to 8100000 spectra for the latter (*n*_s_ = 1 and *n*_p_ = 4) which would require the use of dedicated cores on a super calculator for MCR-ALS minimization.

Finally, the implementation of FF hyperspectral imaging on the ROCK beamline produces ∼30 terabytes of raw data in a week of experiments. The question of big data management, including data accessibility, processing and analysis, is therefore raised. First, given the amount of data produced during a run, users cannot leave the beamline with the data on a hard disk. In this case, the use of Globus API to access the data from outside SOLEIL will allow the user to download their data for further processing/analysis. In that respect, the *Jupyter* notebooks and related Python codes that were developed for data processing will be soon available to the community from Git. However, it means that users must have a storage server large enough to be able to store such amounts of data. Furthermore, data processing, using the *Jupyter* notebooks, and data analysis through MCR-ALS performed on hundreds of thousands of spectra extracted from pixels of hundreds of hyperspectral cubes, need machines that are powerful enough. At SOLEIL, the Data Analysis Remote Treatment Service (DARTS) (Farhi, 2023 ▸) already offers virtual machines connected to the data and to conventional X-ray data processing software with computing capabilities for every SOLEIL user (both internal and external) whereas the Virtual Infrastructure for Scientific Analysis (VISA) (Götz *et al.*, 2023 ▸) is in the process of being tested as a solution for remote data access. More specifically, the *Jupyter* notebook and related Python codes developed for FF hyperspectral imaging data processing and analysis will soon be available in DARTS or VISA using the *JupyterHub* and/or *JupyterLab* framework.

## 5. Conclusions and perspectives

The three case studies presented herein illustrate how the hyperspectral imaging implemented at the ROCK–SOLEIL quick-EXAFS beamline benefits from (i) the versatility of the beamline for adapting the FoV to the sample size and (ii) the stability of the beamline for acquiring a large energy range in a short time frame in full-field X-ray transmission microscopy.

Despite the energy dispersion in the beam footprint associated with a thermal bump on the beamline optics, the stability of the beamline allows us to accurately correct the energy misalignment of the spectra within the FoV in a first step of post-data-processing involving separate, but reproducible, measurement of hyperspectral images for a reference calibration foil, the flat-field and the sample. A set of *Jupyter* notebooks is available to automatize the post-data-analysis of the FF images, including energy alignment, normalization, pixel binning and cube merging. MCR-ALS analysis has been shown to be a unique and powerful tool for isolating the spectra of pure species and the composition from tens of millions of spectra recorded on tens or hundreds of pixelated cubes during the monitoring of a reaction. The full beamline capability coupled with the routine automatization of post-data imaging analysis have been used efficiently for monitoring the dynamic evolution under constraints of micrometre-sized heterogeneity for functional materials belonging to different scientific fields.

If hyperspectral µ-XAS 2D mapping in a DAC has been reported in the past for characterizing natural geological Fe-based samples decomposed under pressure by laser heating (Muñoz *et al.*, 2008 ▸; Aquilanti *et al.*, 2009 ▸), long scanning acquisition (∼100 min map^−1^) with 5 µm steps precluded the study of metastable states. FF quick-EXAFS hyperspectral imaging allows this limitation to be overcome by reporting a complete hyperspectral map in 13 s enabling the dynamic characterization of the pressure-induced spin crossover of a Fe(o-phen)_2_(NCS)_2_ single crystal. Herein we report, to the best of our knowledge, the first evidence that, although small cooperativity is observed, spin-transition induced by pressure is mainly driven by local nucleation.

*In situ* or *operando*X-ray microscopy monitoring of electrode materials cycling has been extensively reported in the last decade highlighting the heterogeneity of the electrochemical reaction encompassing the different length scales of a battery. Second temporal resolution using the acquisition performances of our quick-EXAFS monochromators for FF hyperspectral imaging perfectly matches the common charging rates of electrode materials. The development of heterogeneity in self-supported electrodes can be visualized in real time and under real operation conditions. It can be concluded that the inhomogeneous distribution of species upon electrode cycling makes the establishment of structure/charge-capacity relationships very challenging from non-spatially resolved techniques. In addition, the inherent averaging of inhomogeneity by quick-EXAFS can level the completeness of reactions. It has been clearly demonstrated that quick-EXAFS hyperspectral imaging is a very efficient method for eradicating apparently incomplete reactions on a global scale if the reaction is somewhere complete at the local pixel level.

The development of heterogeneity along catalytic beds has also been deeply scrutinized by X-ray microscopy over the two last decades, passing from the steady-state characterization of heterogeneity (Grunwaldt & Schroer, 2010 ▸) to the dynamic observation of propagation fronts (Alizadehfanaloo *et al.*, 2021 ▸). Our case study not only reveals the temperature gradients inside the catalyst bed linked to the use of the gas blower but also efficiently depicts the radial and axial gradient propagation along the catalytic reactor. Along with our previous work on NiCu-based catalyst (Briois *et al.*, 2024 ▸), the FeCu-based catalyst activations presented herein are, to the best of our knowledge, the first X-ray microscopy mappings reporting the simultaneous dynamic distribution of two elements at the catalytic bed level. The spatially resolved speciation retrieved by chemometrics analysis shows that the reduction process of Fe is locally triggered by the reduction of Cu. In addition, this last case study exemplifies how unique quick-EXAFS FF imaging is for unraveling the complexity of the transformation at both edges thanks to the access of well energy resolved spectra, allowed by the large number of energy points recorded within an energy stack. This permits four iron and three copper species involved in the catalyst activation to be isolated by MCR-ALS and their dynamic and synergic spatial distribution to be described as a function of the temperature increase.

Several improvements to the current acquisition scheme should be in operation in the near future with the scope to extend 2D µm-quick-XAS imaging to 3D µm-quick-XAS imaging. For quick-EXAFS tomography it is necessary that the sample environment is X-ray transparent over a wide angular range (180° if possible). For efficient data acquisition, a continuous scanning approach with simultaneous rotation of the sample and rocking of the monochromator is probably required. In order to synchronize these data, it would be advantageous to record the exact beam energy and projection angle of each projection image for sorting post-acquisition. This is reminiscent of the approach used by Klos *et al.* (2022 ▸). It is interesting to note that 3D-FF spectrotomography with 50 energy points in the 2D XANES projection has been successfully reported in the literature (Ferreira Sanchez *et al.*, 2021 ▸), even for characterization of catalysts under operation conditions (Becher, Ferreira Sanchez *et al.*, 2021 ▸; Becher, Weber *et al.*, 2021 ▸), but with the acquisition time accounting for several hours for one sample limiting its imaging in the steady state. Provided the required sample environment is available at the ROCK beamline for properly rotating the catalytic or electrochemical cell (Becher, Weber *et al.*, 2021 ▸; Liu *et al.*, 2019 ▸), the use of quick-EXAFS FF imaging will be beneficial not only for shortening the time acquisition of 2D XANES projection but also for acquiring XANES spectra with an extended energy range better suited for speciation. However, our target is to offer quick-EXAFS hyperspectral FF tomography for the dynamic study of functional materials, as presented herein for 2D imaging, and this will imply a significant shortening of the acquisition times of hyperspectral cubes to cope with the requirement to rotate the sample at the same time as we record the quick-XAS spectra. The few seconds of acquisition time for a cube could be scaled down first by decreasing the number of images recorded during the half-oscillation period of the monochromator. The third case study presented herein has, for instance, highlighted that valuable information at the Cu *K*-edge can be obtained with only 80 images in the stack. Reducing the number of images in the stack with the same integration time (5 ms) and readout time (4 ms) as those used today would roughly scale down the acquisition time by one order of magnitude (monochromator oscillation frequency at 0.7 Hz). Taking advantage of changing the today-used external triggering of the camera with separate exposure time and readout time to the synchronous readout trigger mode with continuous imaging, approximately twice the number of images could be recorded, and/or using a faster readout camera could also be helpful for reducing time acquisition. All in all, the recording of a quick-EXAFS hyperspectral cube in hundreds of milliseconds should be possible enabling the recording of a tomogram over 180° in a few minutes, thus paving the way for the 3D *operando* monitoring of inhomogeneous reactions (Schroer *et al.*, 2003 ▸; Price *et al.*, 2015 ▸; Ihli *et al.*, 2017 ▸; Becher, Ferreira Sanchez *et al.*, 2021 ▸). Nevertheless, quick-EXAFS tomography will involve the generation of larger datasets than with 2D, because a full-field quick-EXAFS spectrum should be acquired at each tomographic projection angle. This indicates that challenges of data storage and treatment must also be addressed during the design of such an instrument, similarly to a synchrotron tomography beamline.

## Supplementary Material

Supplementary Movie S1. DOI: 10.1107/S1600577524006581/uc5005sup1.mp4

Supplementary Movie S2. DOI: 10.1107/S1600577524006581/uc5005sup2.mp4

Supplementary Movie S3. DOI: 10.1107/S1600577524006581/uc5005sup3.mp4

Supplementary Movie S4. DOI: 10.1107/S1600577524006581/uc5005sup4.mp4

Captions to Supplementary Movies; Supplementary Figures and Tables. DOI: 10.1107/S1600577524006581/uc5005sup5.pdf

## Figures and Tables

**Table 1:** Characteristics of the transmission X-ray microscope for the three functional materials presented as case studies

Case study	Magnification	Pixel size (µm)	FoV (H × V) (mm)	Spatial resolution from Xradia (µm)	Sample–scintillator distance (mm)	Spatial resolution edge profile fit (µm)
1. Spin transition	10×	0.65	1.3 × 0.5	–	8 mm	∼2.5 µm
2. Battery	5×	1.3	2.6 × 1.0	∼2 µm	8 mm	–
3. Catalysis	4×	1.625	3.3 × 1.2	∼4 µm	20 mm	∼6.9 µm

**Table 2:** Summary of recording parameters for one XAS spectrum by FF imaging and by standard quick-EXAFS for the three case studies The number of points per spectrum for standard quick-EXAFS corresponds to the raw number of energy points collected by the PANDABOX hardware interfaced with the monochromator encoder position and providing the trigger line to the 16-bit analog-to-digital converter for synchronously accounting for the ionization chamber currents (Briois *et al.*, 2016 ▸). Interpolation of the oversampled (energies, intensities) values is then performed over an energy grid with a regularly spaced step of 0.25 eV in the XANES range and 2.0 eV in the EXAFS range leading to 1000 to 1500 energy points in each spectrum, depending on the case study. The time resolution for standard quick-EXAFS is equal to the time per spectrum insofar as spectra are acquired in both ascending and descending Bragg angle scanning contrarily to FF imaging for which only image acquisition is performed for ascending Bragg angles.

	Full-field imaging			Standard quick-EXAFS using IC		
Energy range (eV) for case study	Number of images	Time per spectrum	Time resolution	Number of energy points	Time per spectrum	Time resolution
1. Spin transition 7075–7230	500	6.4 s	12.8 s	Not applicable		
2. Battery 7075–7230	500	6.4 s	12.8 s	62500	250 ms	250 ms
3. Catalysis 7070–9090	580	5.56 s	11.1 s	62500	250 ms	250 ms
