# Supplementary material for: Hyperspectral full-field quick-EXAFS imaging at the ROCK beamline for monitoring micrometre-sized heterogeneity of functional materials under process conditions
Source: J Synchrotron Radiat. 2024 Aug 23;31(Pt 5):1084–104. doi: 10.1107/S1600577524006581 (PMC11371034; doi:10.1107/S1600577524006581)
Supplement: Supplementary file 5 [file s-31-01084-sup5.pdf]

JOURNAL OF SYNCHROTRON RADIATION

Volume 31 (2024)

Supporting information for article:

Hyperspectral full-field Quick-EXAFS imaging at the ROCK beamline for monitoring micrometre-sized heterogeneity of functional materials under process conditions

Valérie Briois, Jean Paul Itié, Alain Polian, Andrew King, Aliou Sadia Traore, Eric Marceau, Ovidiu Ersen, Camille La Fontaine, Laurent Barthe, Anthony Beauvois, Olga Roudenko and Stéphanie Belin

Supplementary Movies

Supplementary Movie S1: Spin Transition Case Study

The movie shows the evolution of the HS and LS speciation maps when pressure is applied on the cell membrane from 3 bar (0.63 GPa) to 9 bar (3.40 Gpa). The pixel size is 9.1 µm x 9.1 µm. The fraction of species is related to the color scale at the top of the images. Each image of the movie is the result of a merge of 2 hyperspectral cubes, i.e. 25.6 s corresponding to a membrane pressure increase in the DAC of 0.26 bar.

Supplementary Movie S2: LFP 1C charge monitoring Case Study

The movie shows the evolution of the LFP and FP speciation maps for Electrode 1 and Electrode 2 during 1C charge. The pixel size is 5.2 µm x 5.2 µm. The fraction of species is related to the color scale at the top of the images. Each image of the movie is a result of a merge of 4 hyperspectral cubes, i.e 51.3 s corresponding to the deintercalation of 0.0144 Li by merged cube during the Charge monitoring.

Supplementary Movies S3 and S4: FeCu catalyst activation monitoring Case Study

The movies show the evolution of the 4 Fe species and 3 Cu species speciation maps during activation of the FeCu catalyst heated by a gas blower from RT to 400°C. The pixel size is 32.5 µm x 32.5 µm. The fraction of species is related to the color scale at the top of the images. Each image of the movie corresponds to a single hyperspectral cubes recorded in 11.1 s which is associated to a temperature increase of 1.85 °C. The onset of modifications starts after cube 70 (T = 121°C) and is almost finished after cube 180 (T =300°C).

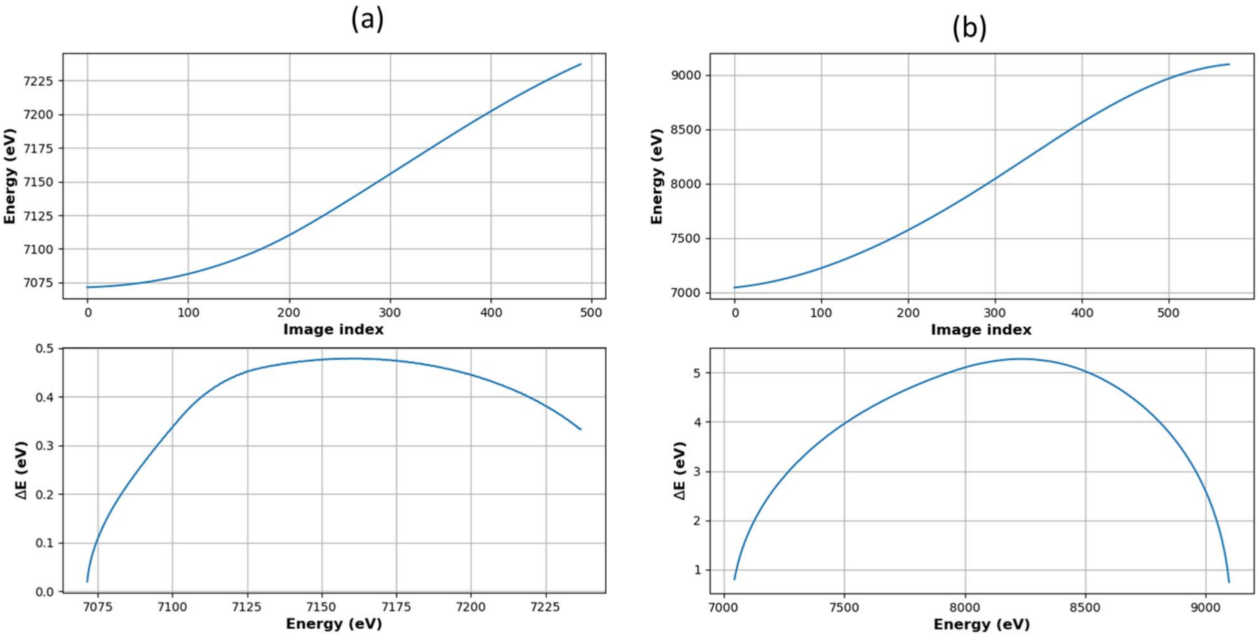

Figure S1 Values of the energy grid relative to the image index (up plot) and energy step relative to the energy values (bottom plot) used for (a) the spin transition complex and the battery electrode materials (oscillation amplitude of the Si(111) channel cut: 0.5°), and, (b) the bimetallic heterogeneous catalyst (oscillation amplitude of the Si(111) channel cut: 3.92°).

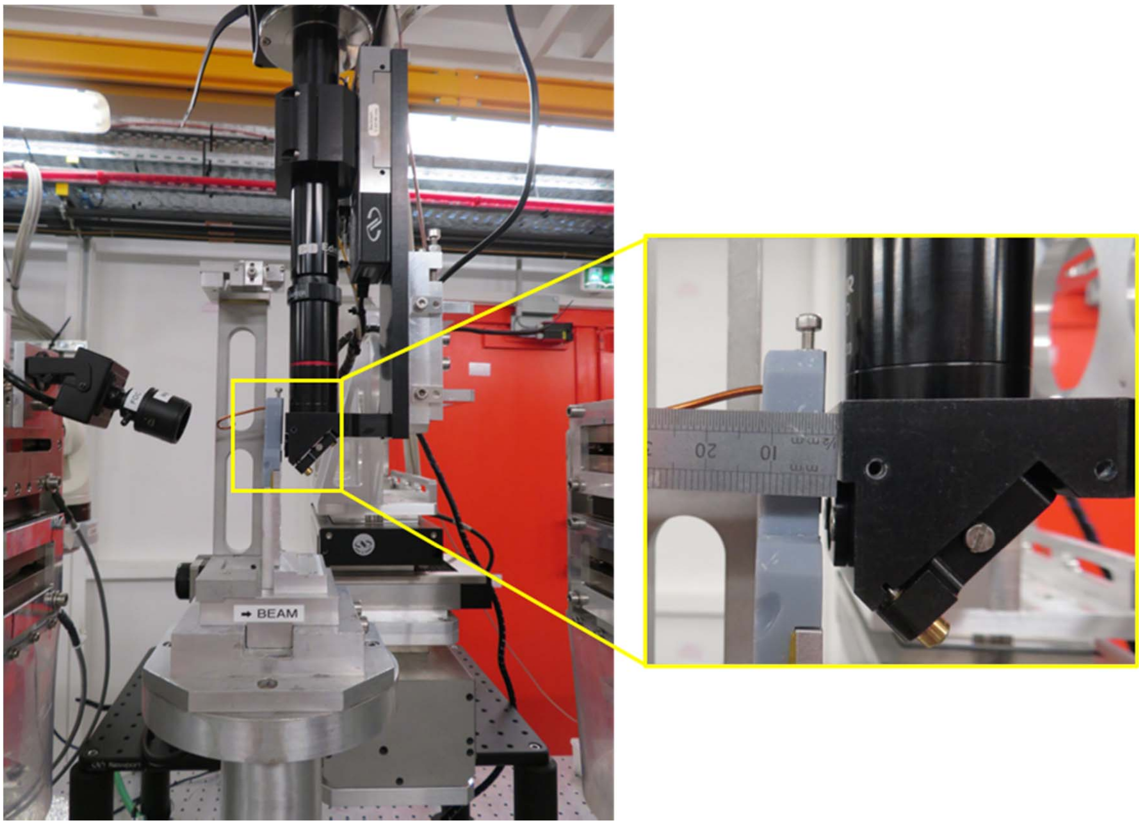

Figure S2 Setup used for measuring the Xradia spatial resolution pattern.

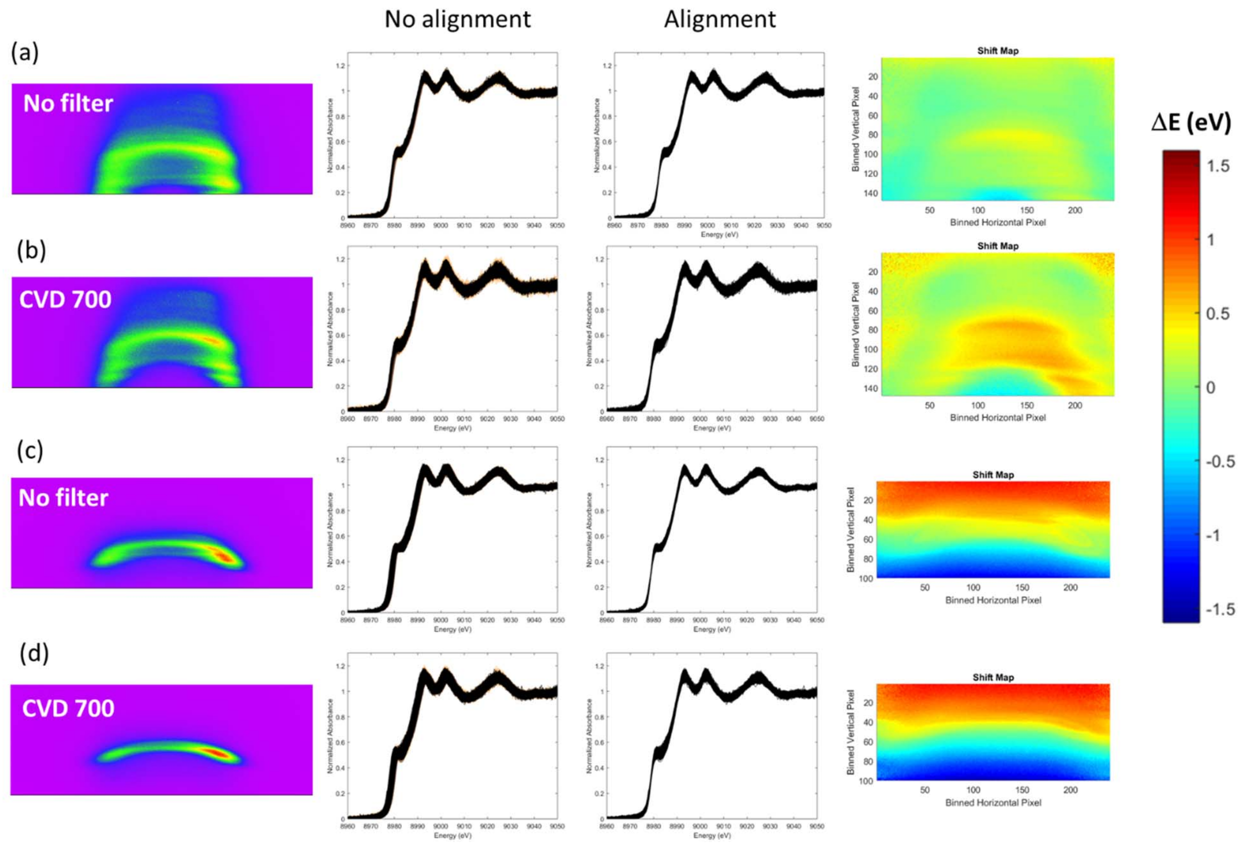

Figure S3 Impact of the dispersion of energy over the beam footprint for different beam sizes and different in-vacuum filters used for reducing the heat load on the monochromators. Measurements where performed at the Cu K edge with a Si(111) channel cut oscillating with an amplitude of 0.4°: (a) and (b) curvarture of the vertically focusing mirror M2b = 160 000 steps. (c) and (d) curvarture of the vertically focusing mirror M2b = 240 000 steps. (a) and (c) no filter to attenuate the beam reflected by the collimating mirror M1. (b) and (d) CVD diamond filter of 700 µm to attenuate the beam reflected by the collimating mirror M1. A Cu reference foil was mesurd in the 4 beam configurations. The spectra were recovered from FF hypersepctral imaging (x4 Navitar magnifiyng objective) considering a 5x5 pixel binning (or 8.125 µm as binned pixel size). The image size for (a) and (b) was 240 pixels x 148 pixels leading to 35 520 spectra. The image size for (c) and (d) was 240 pixels x 100 pixels leading to 24 000 spectra.

First column: I_0 beam images on the ORCA camera.

Second column: Cu reference foil spectra as measured, without alignment by post-processing data.

Third column: Cu reference foil spectra after alignment by post-processing data using the shift maps shown in the Fourth column.

Fourth Column: Shift maps calculated from the alignment of the first derivatives of the spectra measured by hyperspectral imaging with the first derivative of a Cu foil spectrum measured by Quick-EXAFS and absolutely calibrating in energy, *i.e.* with its first derivative maximum set to 8979 eV.

The shift maps are dependent on the use or not-use of filters to attenuate the beam reflected by the collimating M1 mirror and impinging the channel cut monochromator. For a given curvature of the vertically focusing M2b mirror, less attenuated the beam is, larger the size of the beam at the sample is, and, smaller the dispersion is. The dependence of the energy dispersion with the change of heat load on the channel-cut (using or nor the CVD 700) suggests a thermal bump on the channel cut not well compensated by its indirect water cooling and possibly amplified by the slow oscillation of the crystal (0.09 Hz) used for FF imaging. Indeed, the curvature of the first collimating M1 mirror has been optimized using reference foils in standard Quick-EXAFS in operation at 2Hz channel cut oscillation for achieving an optimal energy resolution on the spectra, irrespective of the choice of the filters. It is possible that for the slow monochromator oscillation (0.09 Hz), the curvature of M1 for minimizing the energy dispersion could be different to the one optimized at 2Hz and should be consequently further refined. Anyway, in response to the observed energy dispersion, we have developed a robust strategy to get rid of the problem and demonstrated by the results obtained with the different case studies its efficiency for the alignment of the spectra as post-treatment.

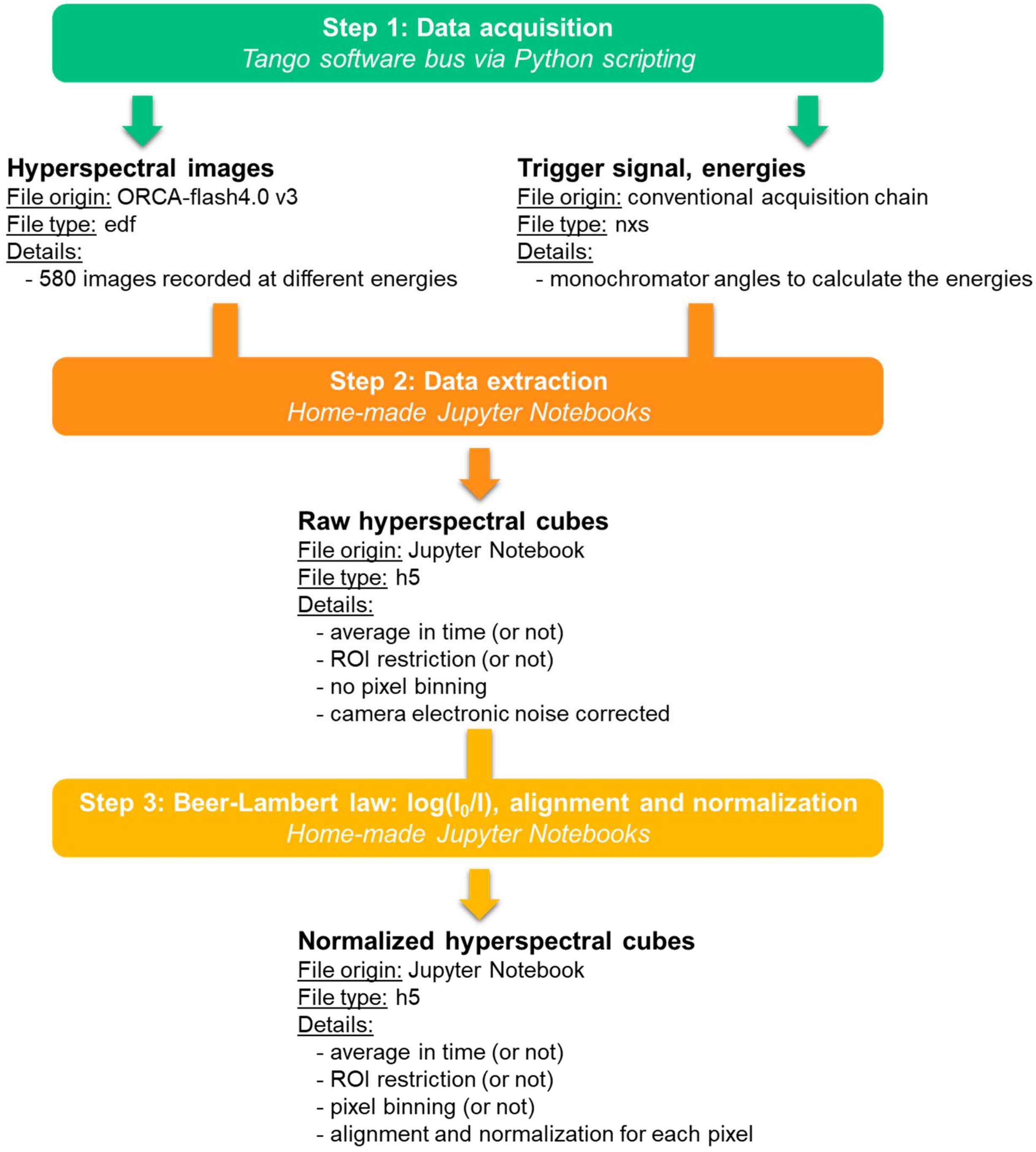

Figure S4 Schematic representation of the different data processing step (from data acquisition to normalized and aligned spectra) during a FF hyperspectral Quick-EXAFS acquisition

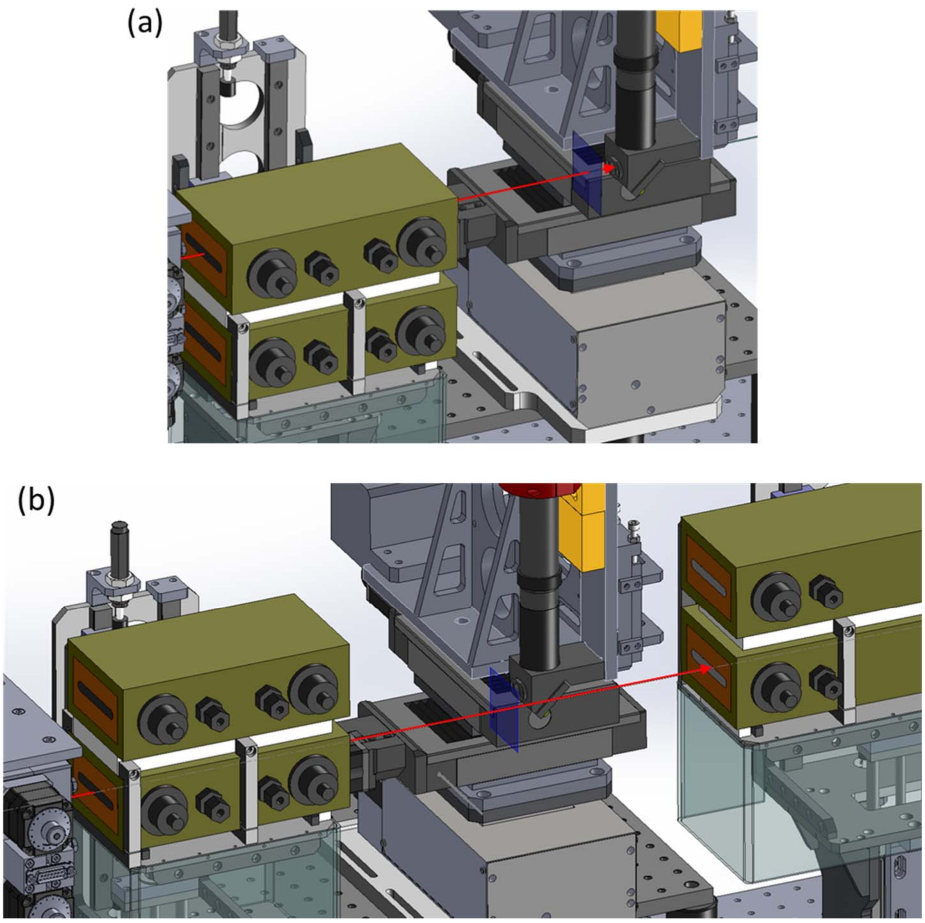

Figure S5 Configuration of the detectors (camera for hyperspectral imaging and ionization chambers) with respect of the sample. (a) for hyperspectral imaging, the beam hits the camera after passing the first ionization chamber. The first IC is filled with light gas (He:N_2 in 50:50 proportions) irrespective to the X-ray energy, to minimize the absorption of photon before the sample. The I_0 signal recorded with the IC in the imaging configuration and the external TTL signal triggering the acquisition of images are synchronously recorded and saved in a nexus file together with the encoded angles of the monochromator. Recording a I_0 with IC during FF hyperspectral acquisition enables to further perform a pre-alignment in energy of the hyperspectral cubes using a monochromator glitch that has been absolutely calibrated in energy. The external TTL signal triggering the images acquisition by the camera will be used during the data extraction process to correlate each image of the hyperspectral cube with the corresponding angle of the monochromator, *i.e.* with the energy. (b) for standard Quick-EXAFS, the camera is moved out of the way of the beam letting it passing through the second ionization chamber. To this purpose, a horizontal stroke of -50 mm compared to the position corresponding to the beam at the center of the pixel array sensor is used. At the same time that the camera is moved out, the second set of ionization chambers, originally designed for the “edge jumping” capability of the ROCK beamline (Briois *et al*., 2016) and filled with optimal gas composition according to the threshold energy of the absorbing element for recording I_0, I_{sample} and $I_{reference}$ in conventional Quick-EXAFS transmission mode is installed in front of the beam. This

conventional Quick-EXAFS configuration allows for quickly monitoring the state of the material at the end of a reaction previously monitored by FF hyperspectral imaging for which data analysis is slower and more complex than the one of conventional Quick-EXAFS.

Table S1 Normalization Parameters for the Fe K edge spectra recorded for the Spin Transition Complex

	Fe K edge E_0 = 7112 eV
Linear pre-edge function	From 7075 to 7108 eV
Post-edge polynomial Fitting	From 7140 to 7230 eV – 1st order

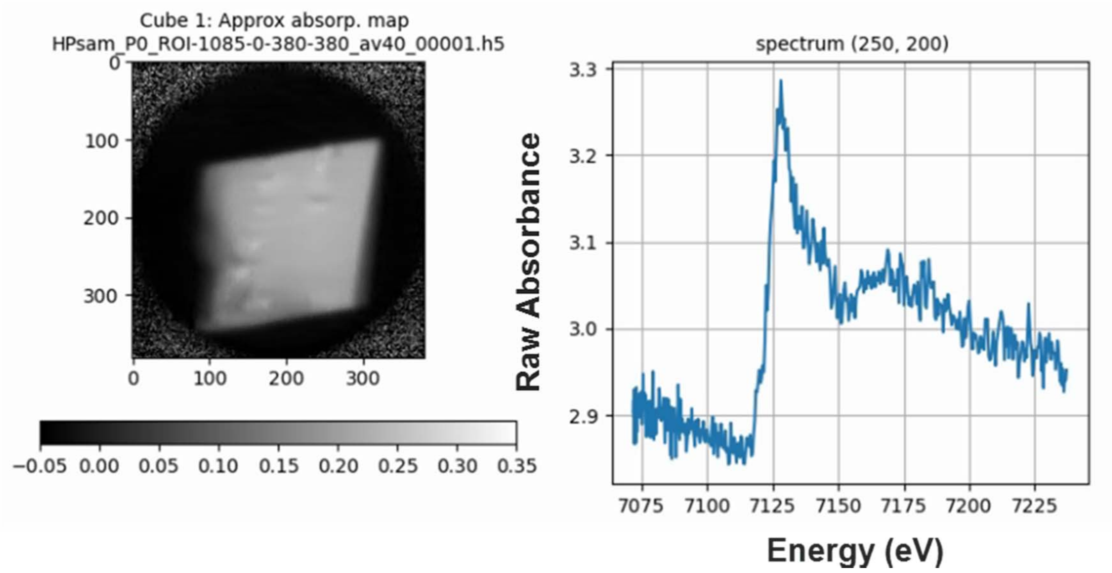

Figure S6 Contrast absorption map of the sample inside the DAC corresponding to the merge of 40 hyperspectral cubes and typical raw spectrum at a pixel of the image (Px = 250 and Py = 200). Herein the pixel size in the absorption map is 0.65 µm, *i.e.* the pixel size of the camera (6.5 µm) magnified by the x10 Mitutuyo objective.

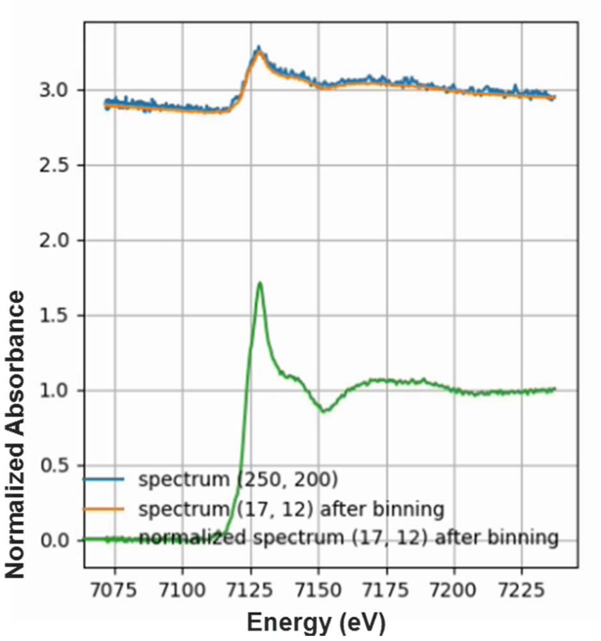

Figure S7 Quality of the spectra after a 10x10 pixel binning (leading to spatial resolution of 6.5 µm x 6.5 µm) and normalisation. A linear pre-edge function calculated between 7075 and 7108 eV and extrapolated through the post-edge region was substracted to the raw data and linear post-edge function fitted between 7140 and 7230 eV was used for normalisation.

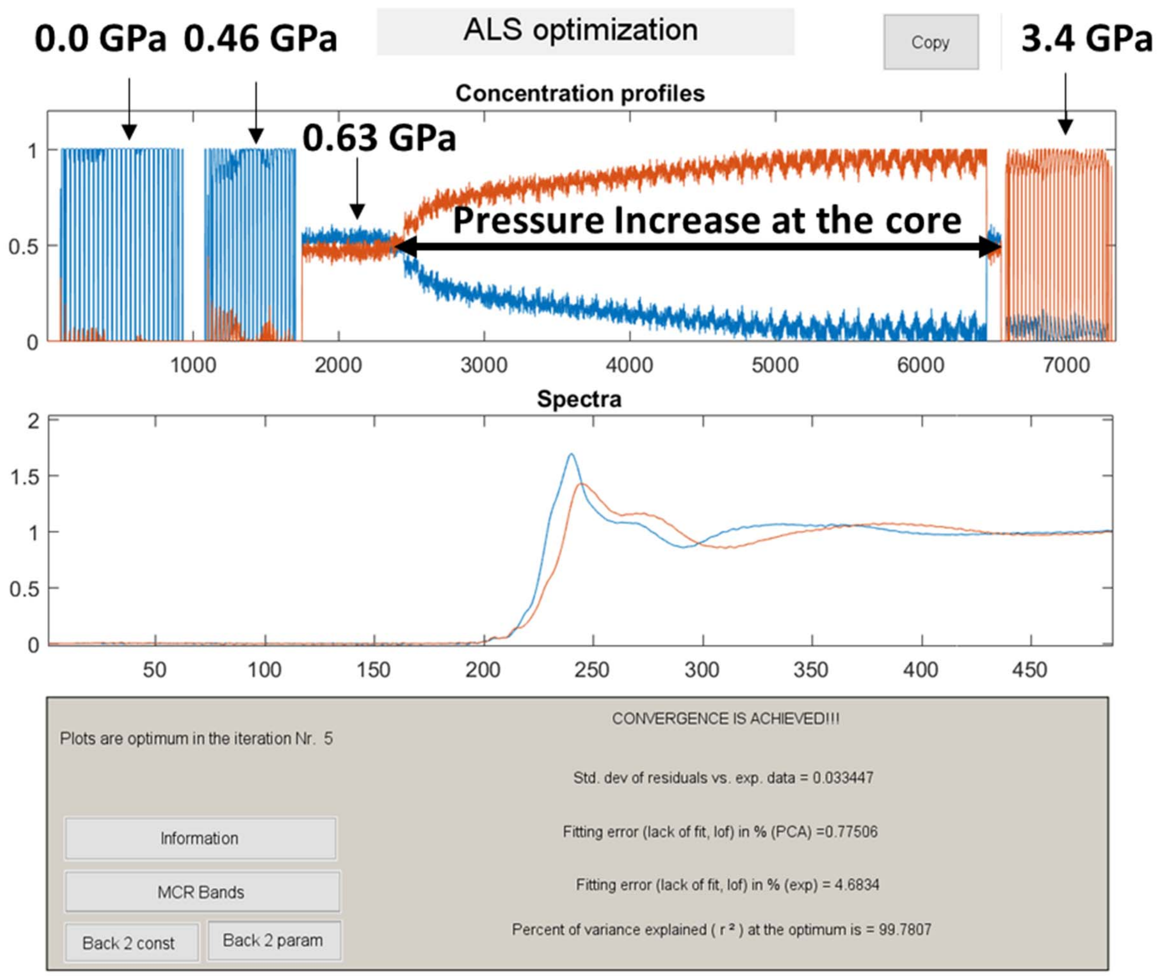

Figure S8 Outcome of the MCR-ALS minimization at the Fe K edge of the data set measured by hyperspectral imaging during the spin crossover monitoring. MCR-ALS was carried out considering all the spectra recorded with a spatial resolution of 6.5 x 6.5 µm² (binning of pixels: 10x10) for P = 0, 0.46, 0.63 and 3.40 GPa over the full single crystal image and over 65 x 65 µm^2 area at the center of the crystal for images measured during pressure increase. The MCR-ALS minimization was performed using as guessed matrix of spectra the one buit from the HS and LS spectra obtained by averaging the spectra measured at P=0 and P=3.4 GPa. Closure relation on the concentrations of both components, non negativity of concentration and spectra were used for minimization.

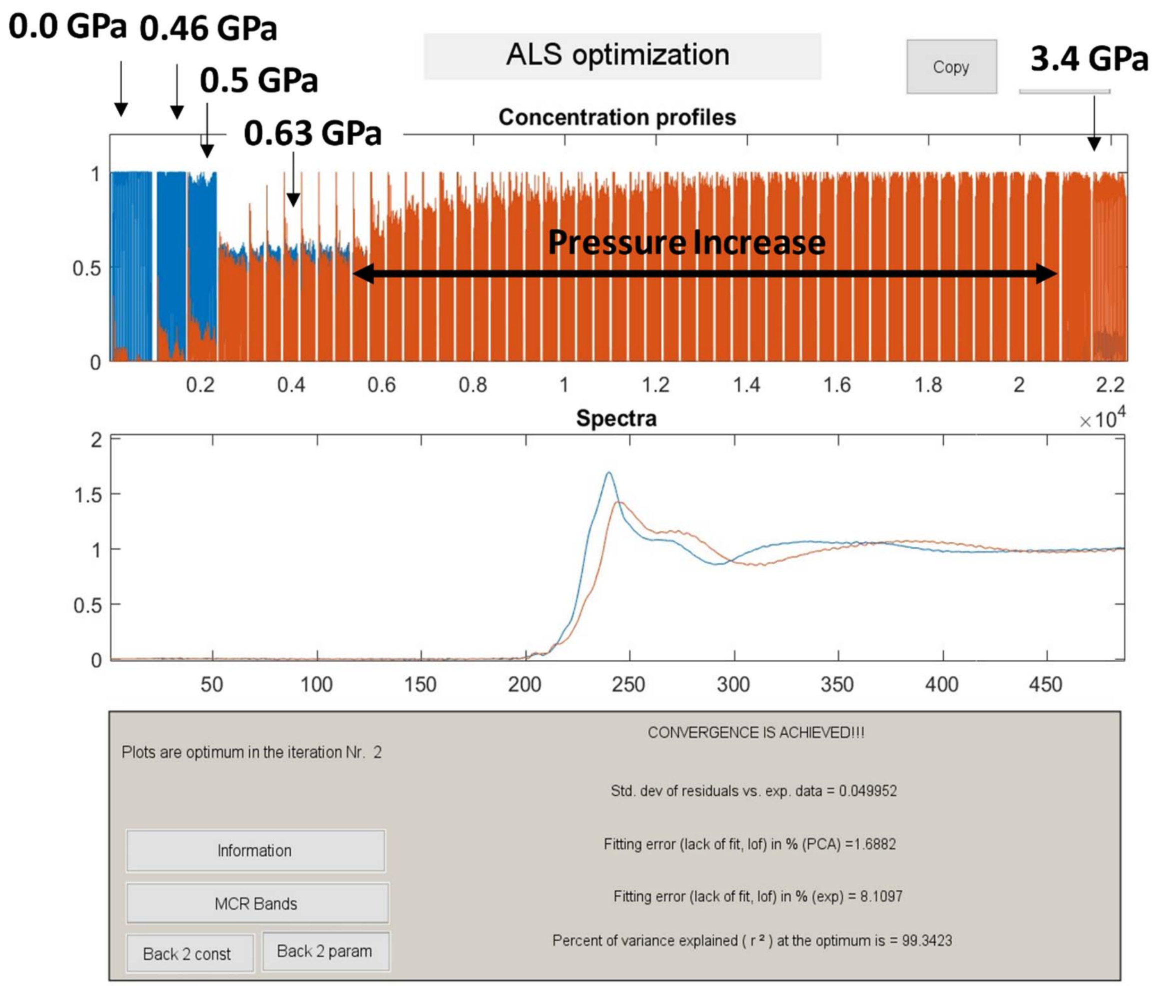

Figure S9 Outcome of the minimization performed for recovering the map distribution shown in Figure 6 and in the movies. The spectra determined by the minimization shown in Figure S8 were used for minimizing the matrix containing the spectra recorded under isobar conditions at P=0, 0.46, 0.50, 0.63 and 3.4 GPa (with pixel binning 10x10) together with the spectra recorded during the pressure increase (pixel binning 14x14). For the former between 10 to 40 hyperspectral cubes were merged to improve the S/N ratio, whereas for the dynamic monitoring of the pressure increase, only two hyperspectral cubes were merged leading to 47 time-averaged cubes recorded during the 20 minutes of pressure increase.

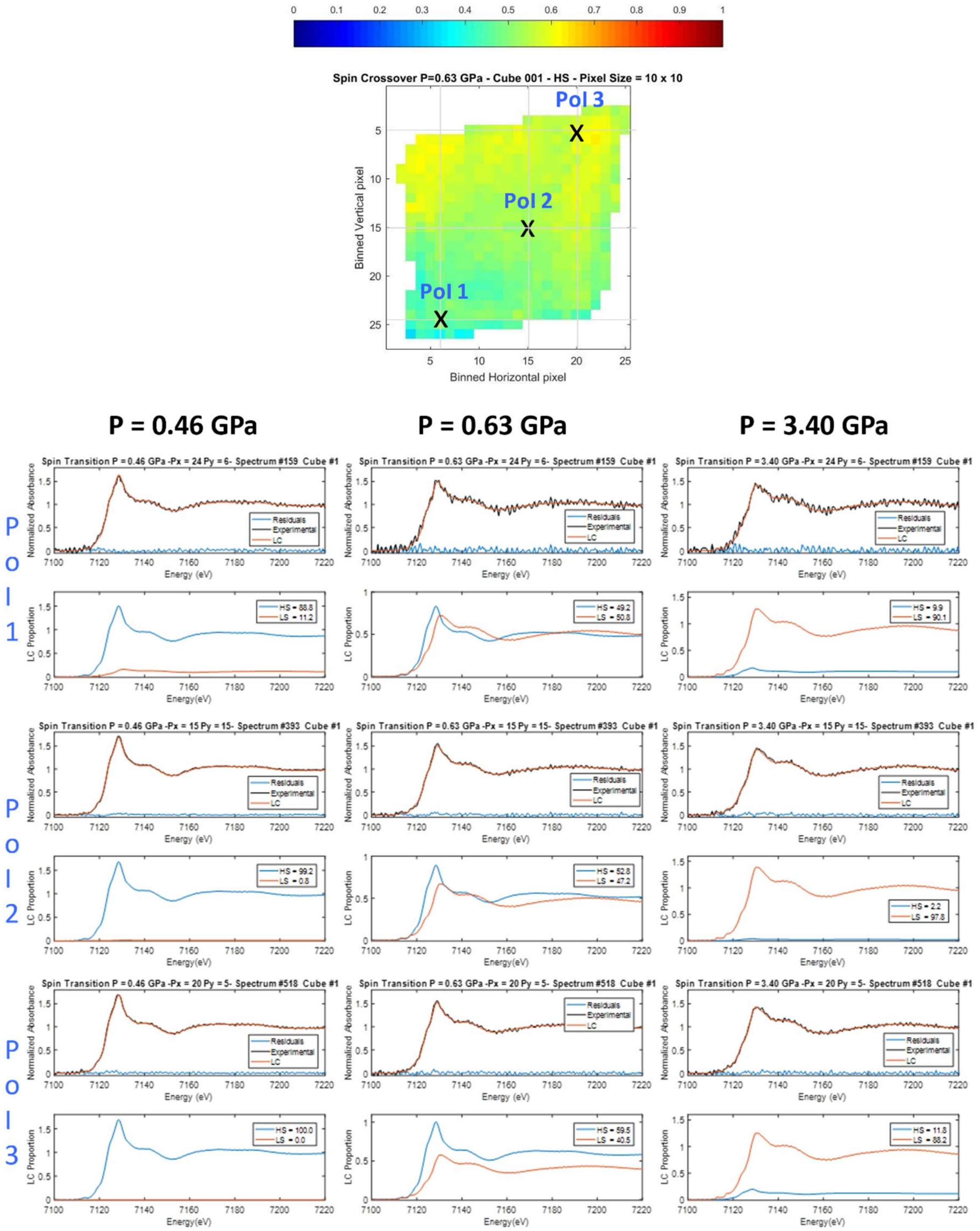

Figure S10 Comparison, for the cubes, shown in Figure 6, corresponding to steady-state characterizations at P=0.46, 0.63 and 3.40 GPa of the spin transition single crystal, of the experimental spectrum (black line), recorded at the different pixels (PoI) located on the map displayed at the top by crosses, with the MCR-ALS rebuilt spectrum (red line) and corresponding residuals (blue line) calculated from the difference between the experimental spectrum and rebuilt one. The weighted spectral contribution of HS (blue spectrum) and LS (red spectrum) components used to rebuild each spectrum is also reported at the bottom plot for each pixel and pressure condition.

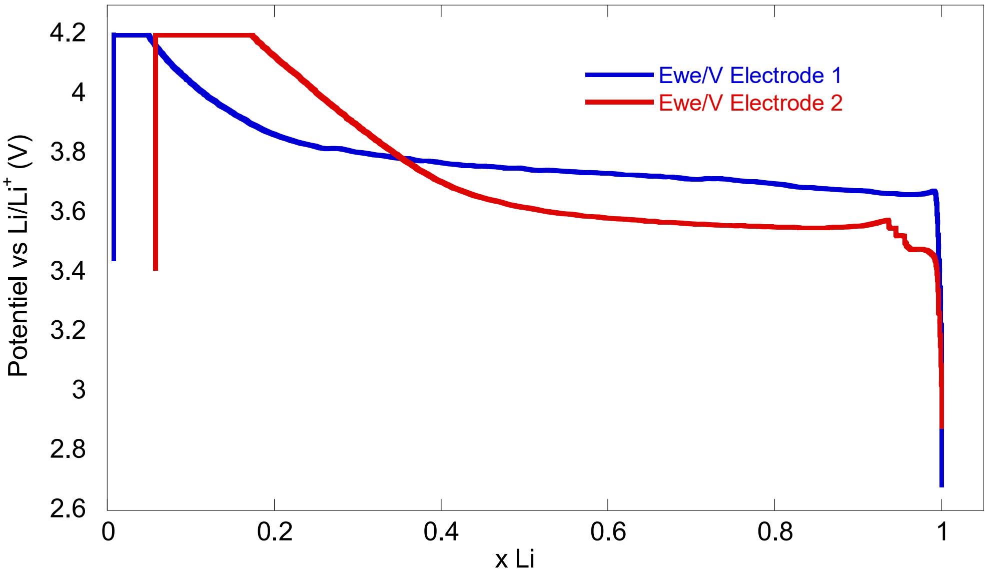

Figure S11 Galvanostatic charge of both LFP electrodes during the *operando* FF quick-XAS Imaging. Voltage versus x of Li^+ extracted with 1C rate.

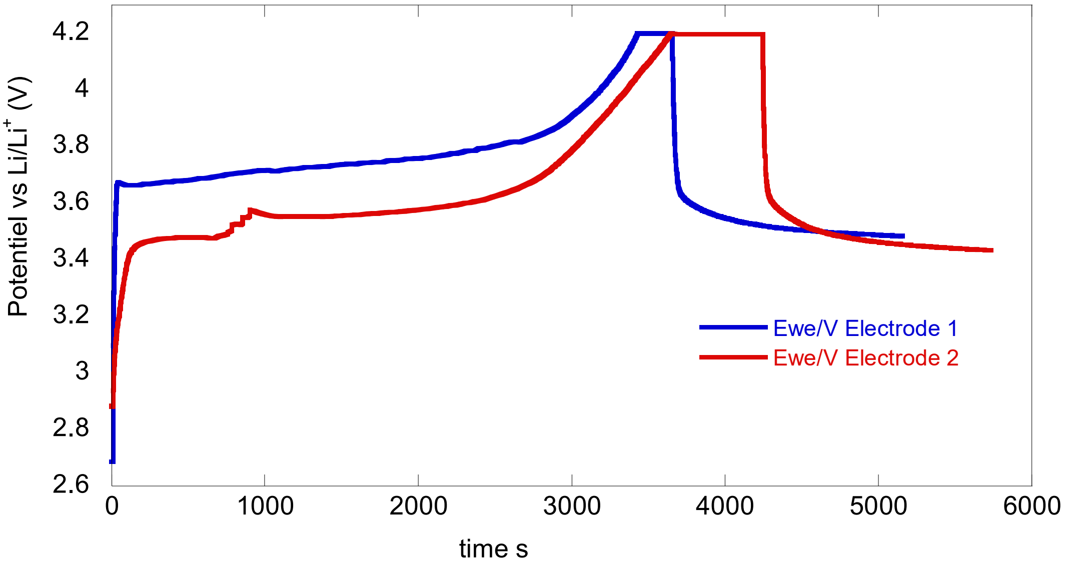

Figure S12 Galvanostatic charge of both LFP electrodes during the *operando* FF quick-XAS Imaging. Voltage versus time with 1C rate.

Table S2 Normalization Parameters for the spectra recorded at the Fe K edge for LFP Electrodes

	Fe K edge E_0 = 7112 eV
Linear pre-edge function	From 7075 to 7108 eV
Post-edge polynomial Fitting	From 7140 to 7230 eV – 1st order

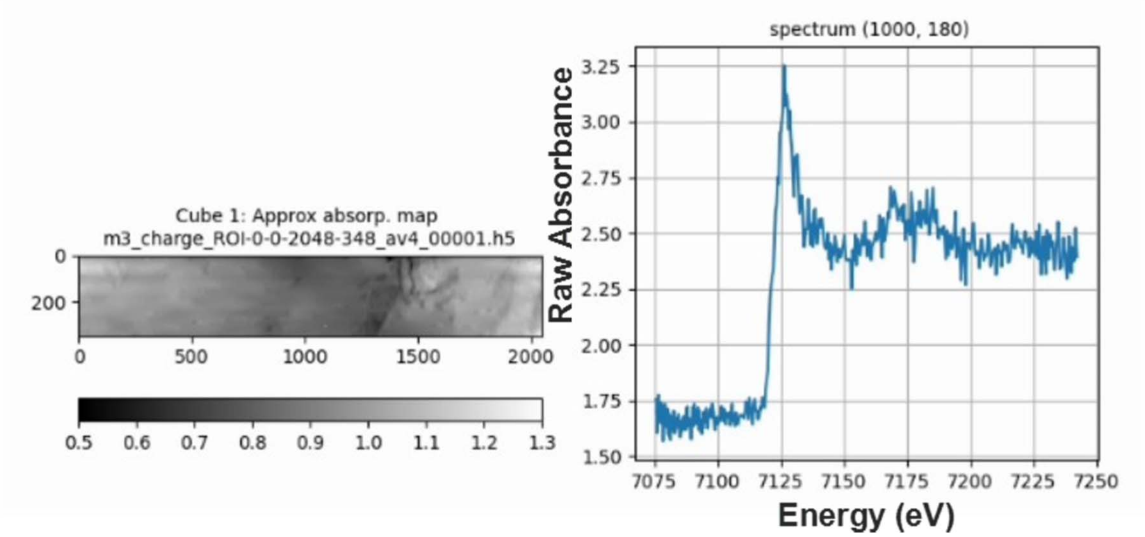

Figure S13 Contrast absorption map of the Electrode 2 corresponding to the merge of 4 hyperspectral cubes and typical raw spectrum at a pixel of the image (Px = 1000 Py = 180). Herein the pixel size is the pixel size of the camera magnified by the x5 Mitutuyo objective *i.e.* 1.3 µm.

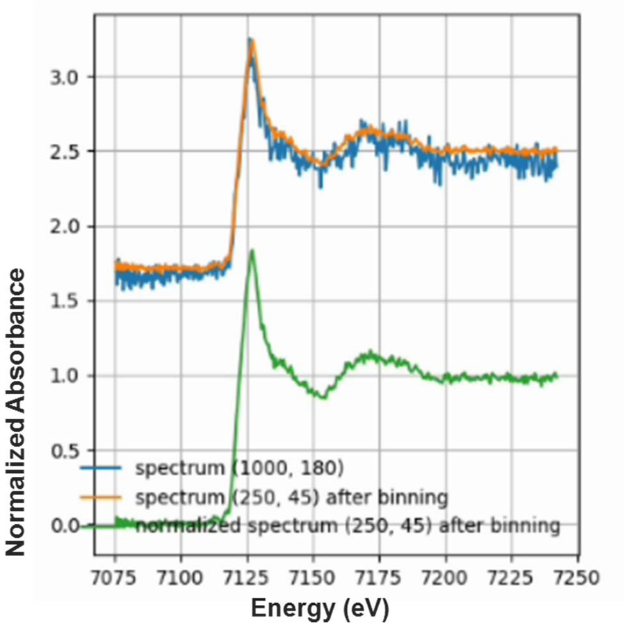

Figure S14 Quality of the spectra after a 4x4 pixel binning and normalisation. A linear pre-edge function calculated between 7075 and 7108 eV and extrapolated through the post-edge region was

substracted to the raw data and linear post-edge function ftted between 7140 and 7230 eV was used for normalisation.

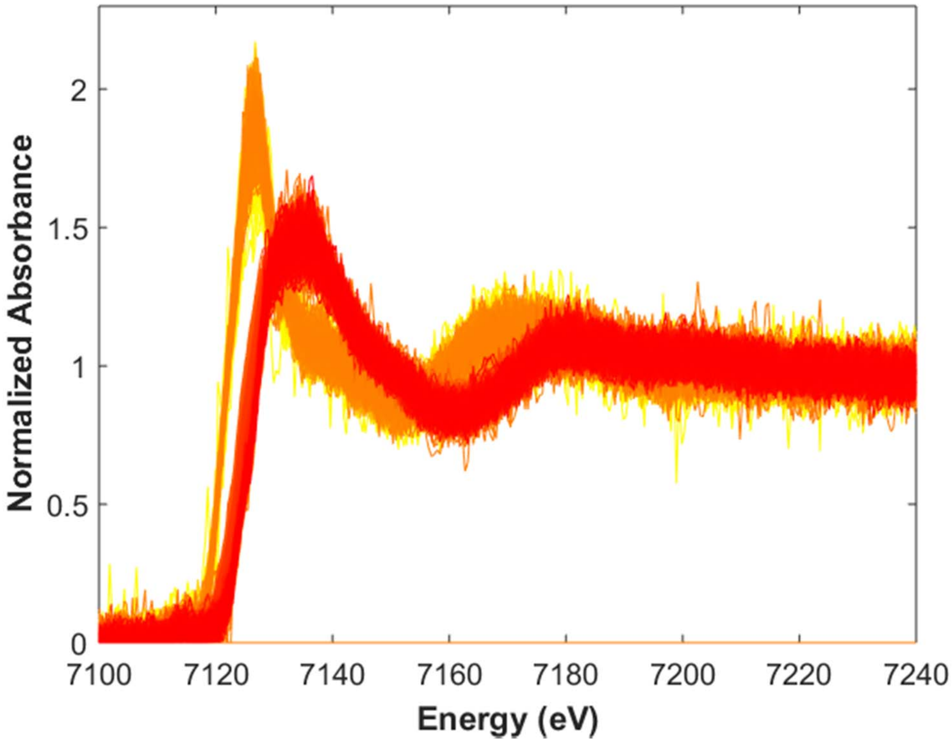

Figure S15 Experimental spectra extracted from the Cube 1 and Cube 85 for Electrode 1. The total number of spectra in both cubes amounts for 89 088 spectra. Only 1 spectrum over 10 is displayed in Figure S15.

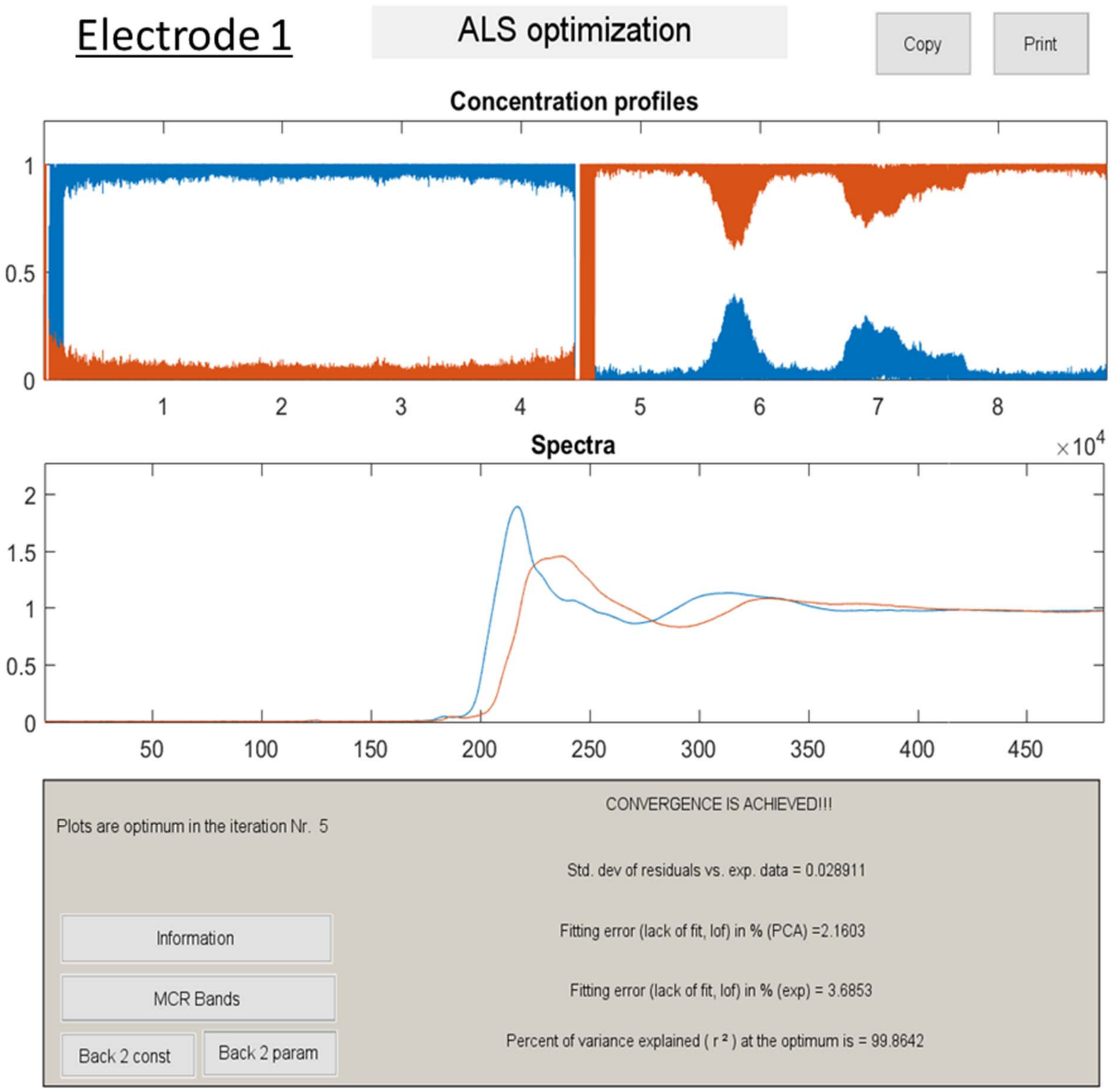

Figure S16 Outcome of the MCR-ALS minimization at the Fe K edge of the dataset measured by hyperspectral imaging for the first and last cubes of the 1C charge of Electrode 1 with a spatial resolution of 5.2 x 5.2 µm² (binning of pixels : 4x4). MCR-ALS was performed using as guessed matrix of spectra the one buit by averaging the spectra in each cube. Closure relation on the concentrations of both components, non negativity of concentration and spectra were used for minimization.

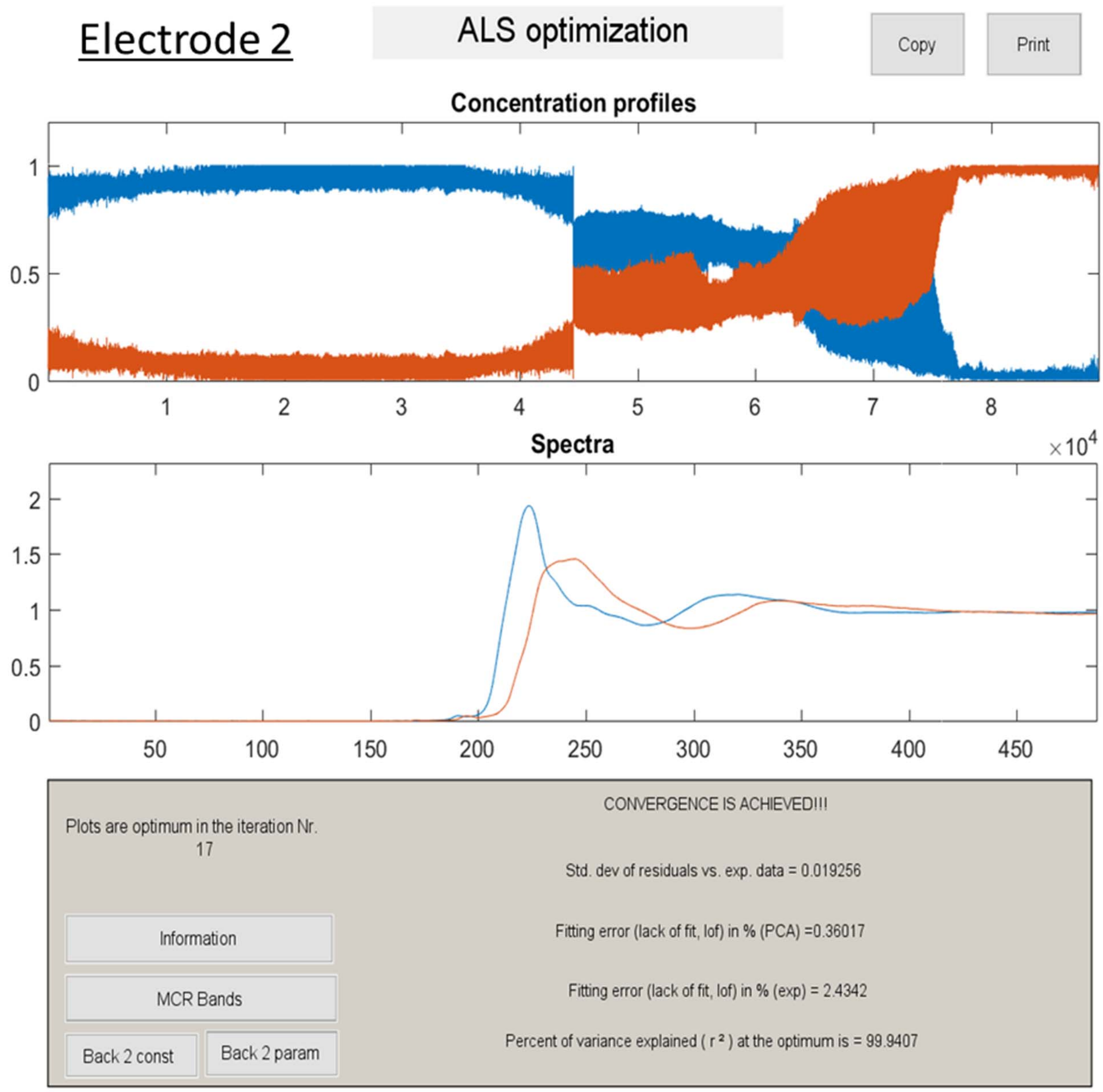

Figure S17 Outcome of the MCR-ALS minimization at the Fe K edge of the dataset measured by hyperspectral imaging for the first and last cubes of the 1C charge of Electrode 2 with a spatial resolution of 5.2 x 5.2 µm² (binning of pixels : 4x4). MCR-ALS was performed using as guessed matrix of spectra the one buit by averaging the spectra in each cube. Closure relation on the concentrations of both components, non negativity of concentration and spectra were used for minimization.

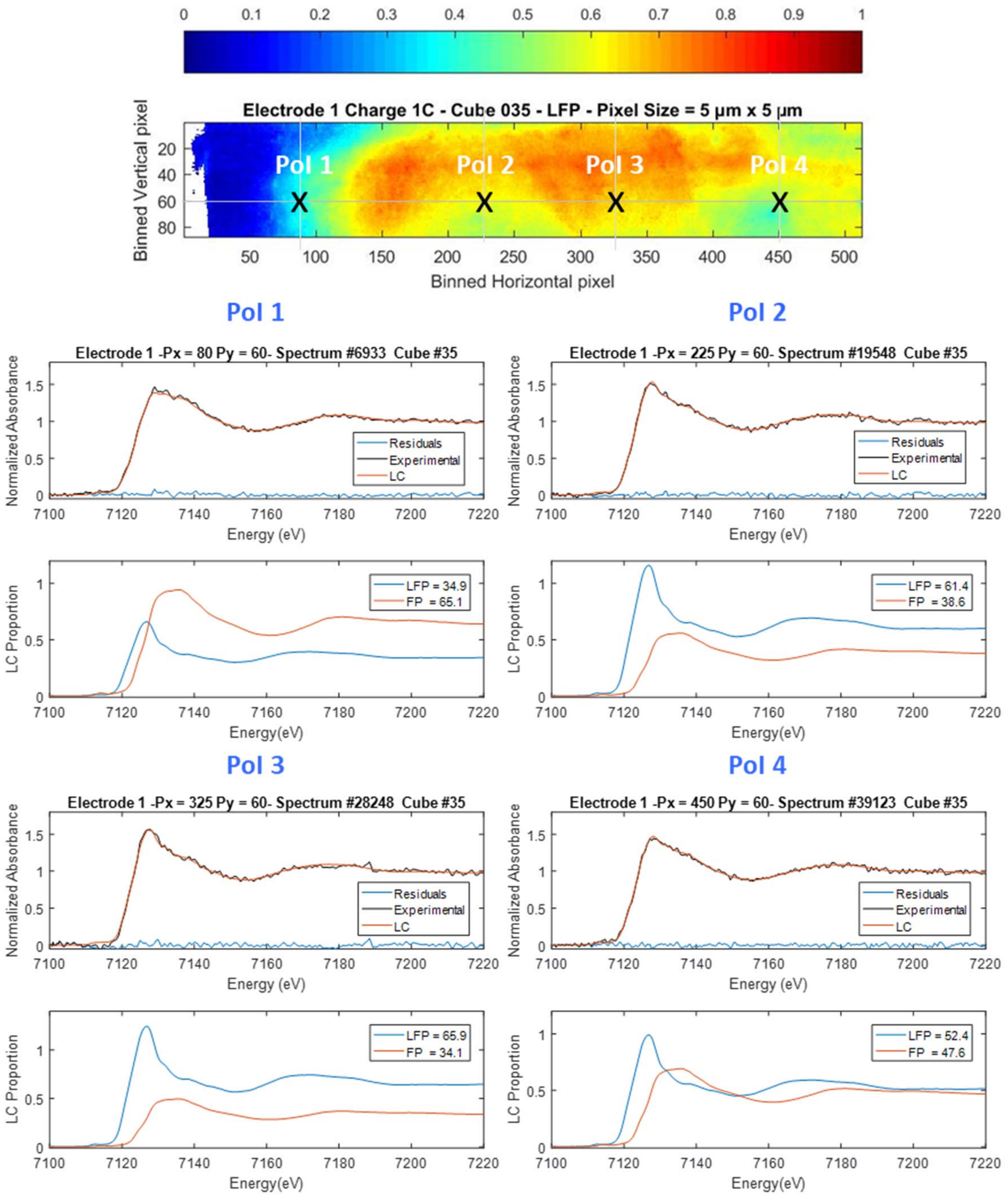

Figure S18 Comparison, for the Cube 35 recorded at voltage corresponding to half Lithium deintercalated during 1C charge of Electrode 1 (Figure 11), of the experimental spectra (black line) recorded at the different pixels (PoI) located on the map displayed at the top by crosses with the MCR-ALS rebuilt spectra (red line) and corresponding residuals (blue line) calculated from the difference between the experimental spectrum and rebuilt one. The weighted spectral contribution of LFP and FP components used to rebuild each spectrum is also reported at the bottom plot for each pixel.

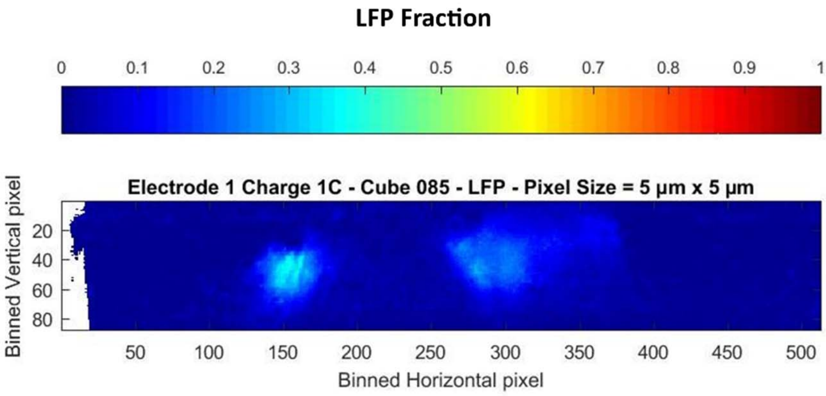

Figure S19 Distribution map of fraction of LFP at the end of the 1C charging of Electrode 1 (Cube 85). The fraction value is associated to the color in the color scale at the top.

Table S3 Normalization Parameters for the FeCu catalyst bed

	Fe K edge E_0 = 7112 eV	Cu K edge E_0 = 8979 eV
Linear pre-edge function	From 7062 to 7092 eV	From 8900 to 8960 eV
Post-edge polynomial Fitting	From 7152 to 8412 eV – 6th order	From 9010 to 9090 eV – 1st order
FT extraction	k_{min} = 3.8 Å^{-1} – k_{max} = 11.1 Å^{-1}<br>Kaiser window, dk = 2	

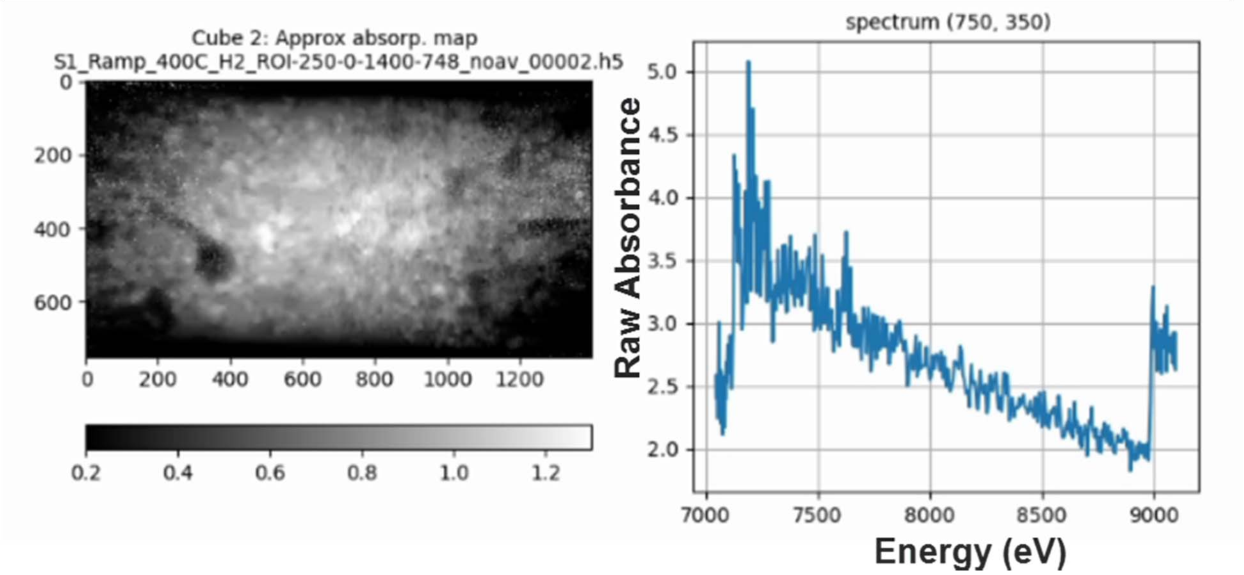

Figure S20 Contrast absorption map of the catalyst bed for a single cube and typical raw spectrum at a pixel of the image (Px= 750 and Py = 350). Herein the pixel size is the pixel size of the camera magnified by the x4 Navitar objective *i.e.* 1.625 µm.

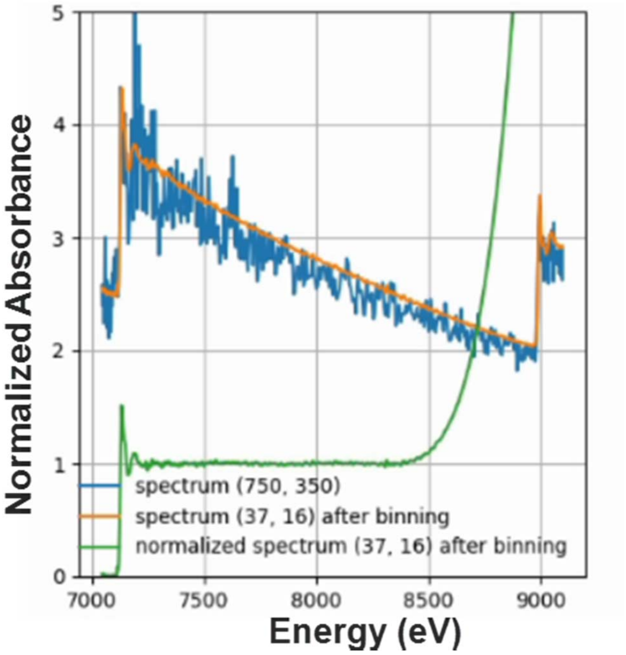

Figure S21 Quality of the spectra after a 20x20 pixel binning and normalisation with the Fe K edge parameters. A linear pre-edge function calculated between 7062 and 7092 eV and extrapolated through

the post-edge region was substracted to the raw data and polynomial function ftted between 7152 and 8412 eV was used for normalisation.

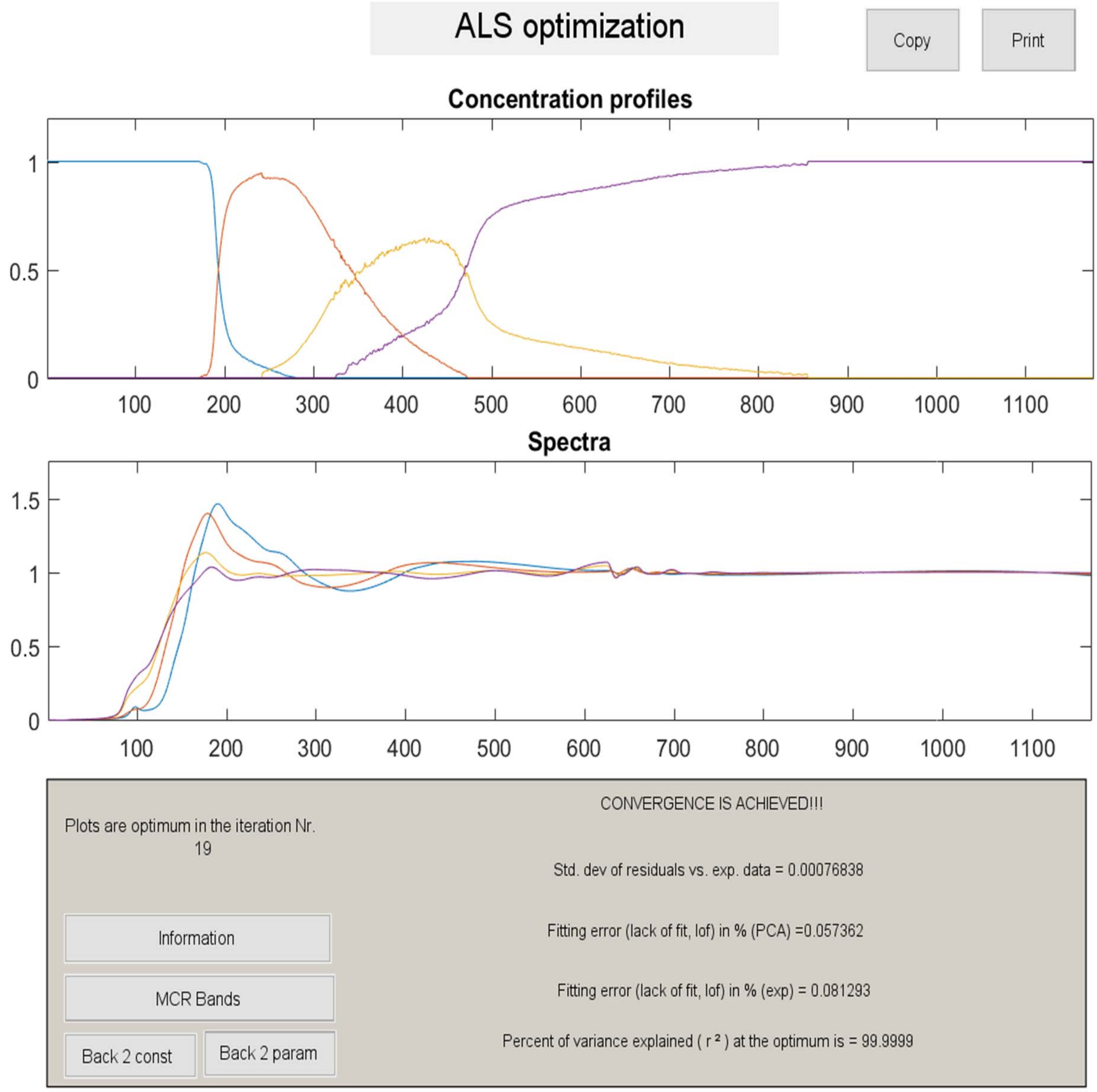

Figure S22 Outcome of the MCR-ALS minimization at the Fe K edge of the data set measured by Quick-EXAFS during the heating of the bimetallic $FeCu/SiO_2$ catalyst under H_2 and presented Figure 14 (a). Evolving Factor Analysis used for guessing the concentration matrix. Constraints: non negativity of C and S, unimodality for C and closure relation for C.

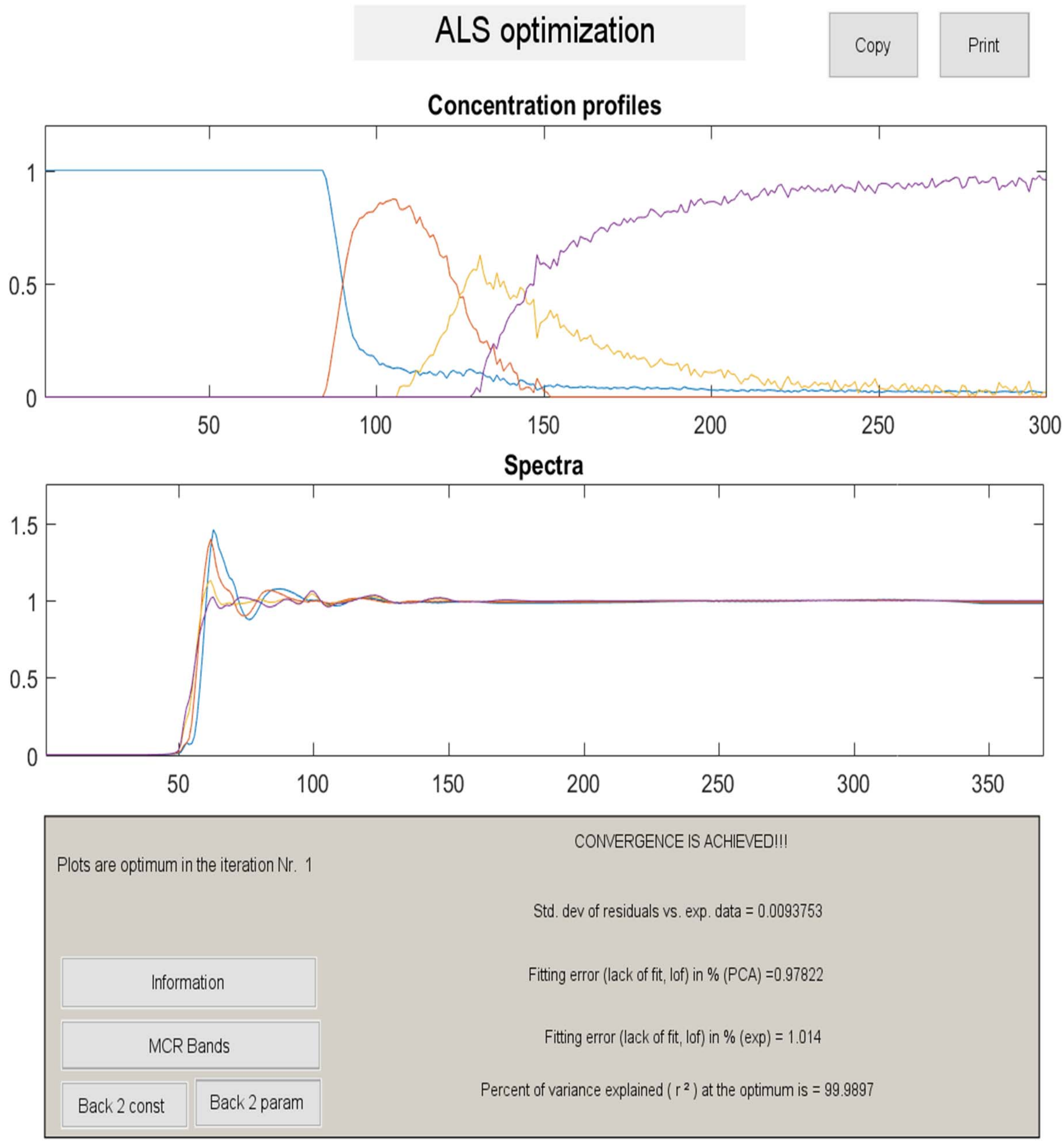

Figure S23 Outcome of the MCR-ALS minimization at the Fe K edge of the data set measured by hyperspectral imaging during the heating of the bimetallic FeCu/SiO_2 catalyst under H_2 and presented Figure 14(b). The spectra of this data set has been obtained by merging all the spectra of each pixelated image. The minimization was carried out by conisdering as guessed S matrix the matrix of spectra obtained from the Quick-EXAFS data set shown in Figure 14(a) but projected on the energy grid of the hyperspectral imaging. Constraints: non negativity of C and S, unimodality for C, closure relation for C and lower or equal constraint with the guessed S matrix.

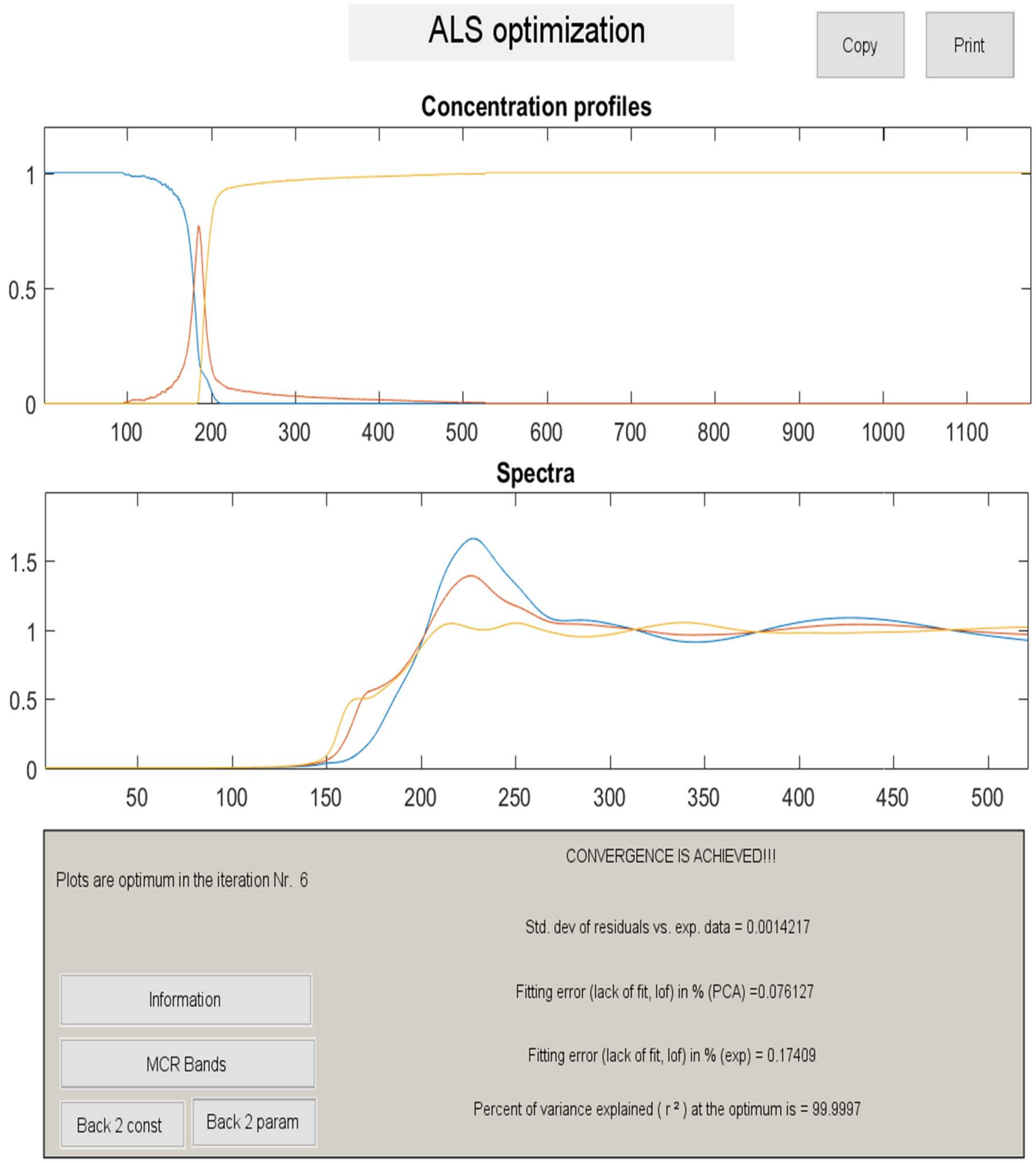

Figure S24 Outcome of the MCR-ALS minimization at the Cu K edge of the data set measured by Quick-EXAFS during the heating of the bimetallic FeCu/SiO_2 catalyst under H_2 and presented Figure 14(c). Evolving Factor Analysis used for guessing the concentration matrix. Constraints: non negativity of C and S, unimodality for C and closure relation for C.

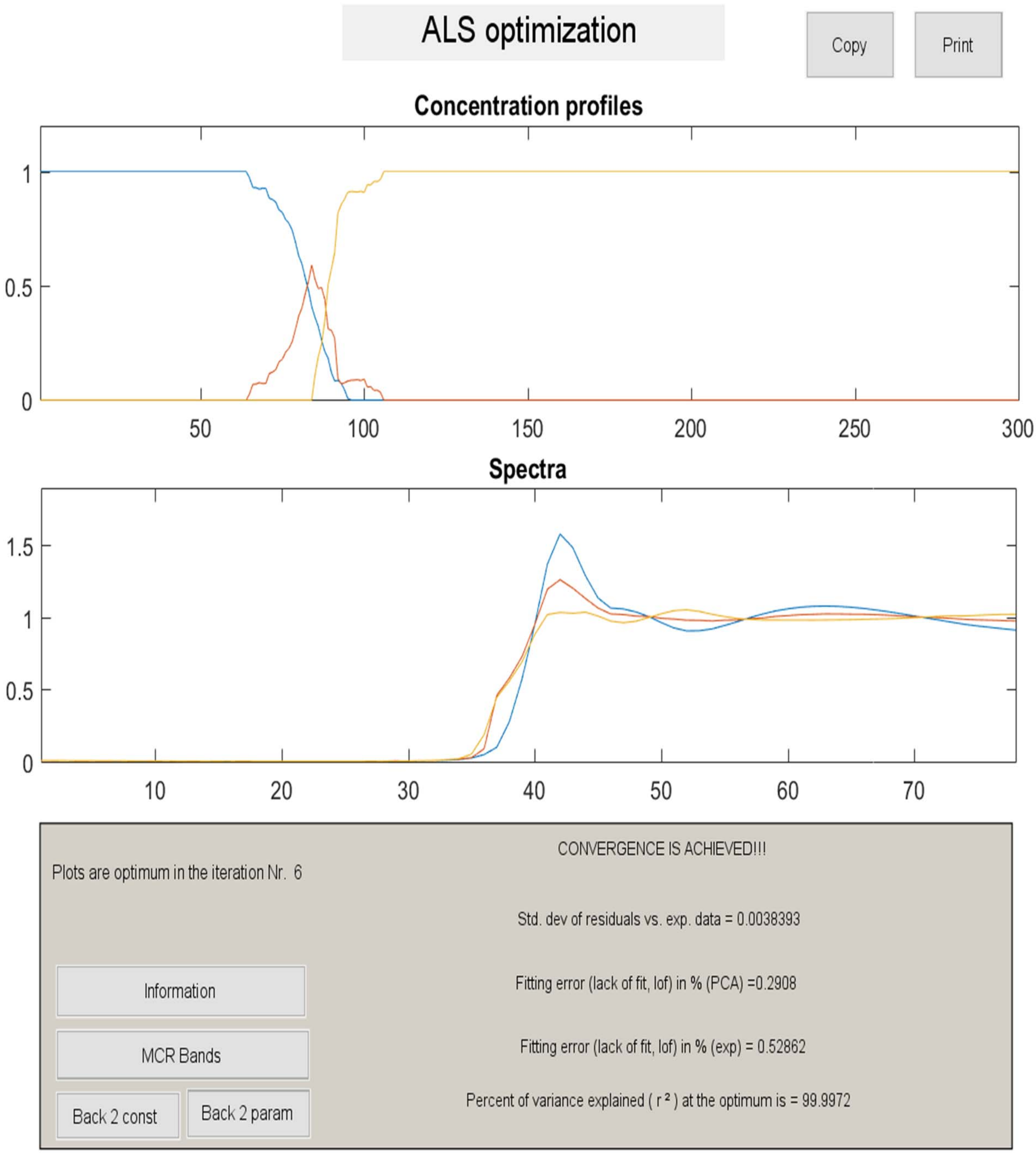

Figure S25 Outcome of the MCR-ALS minimization at the Fe K edge of the data set measured by hyperspectral imaging during the heating of the bimetallic $FeCu/SiO_2$ catalyst under H_2 and presented Figure 14 (d). The spectra of this data set has been obtained by merging all the spectra of each pixelated image. Evolving Factor Analysis used for guessing the concentration matrix. Constraints : non negativity of C and S, unimodality for C and closure relation for C.

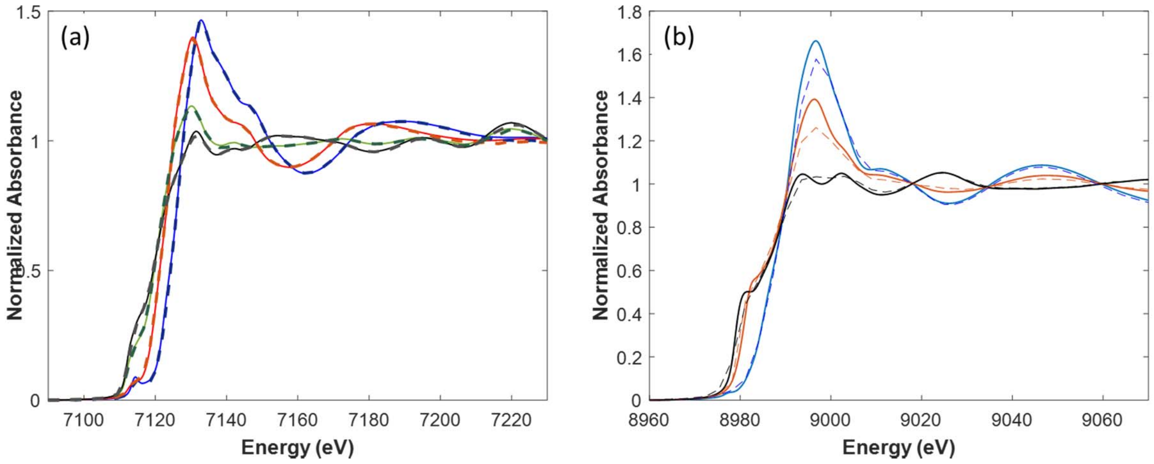

Figure S26 Comparison of the pure spectra isolated by MCR-ALS (a) at the Fe K edge for minimization carried out in Figures S22 (full lines) and S23 (dashed lines) and (b) at the Cu K edge for minimization carried out in Figures S24 (full lines) and S25 (dashed lines).

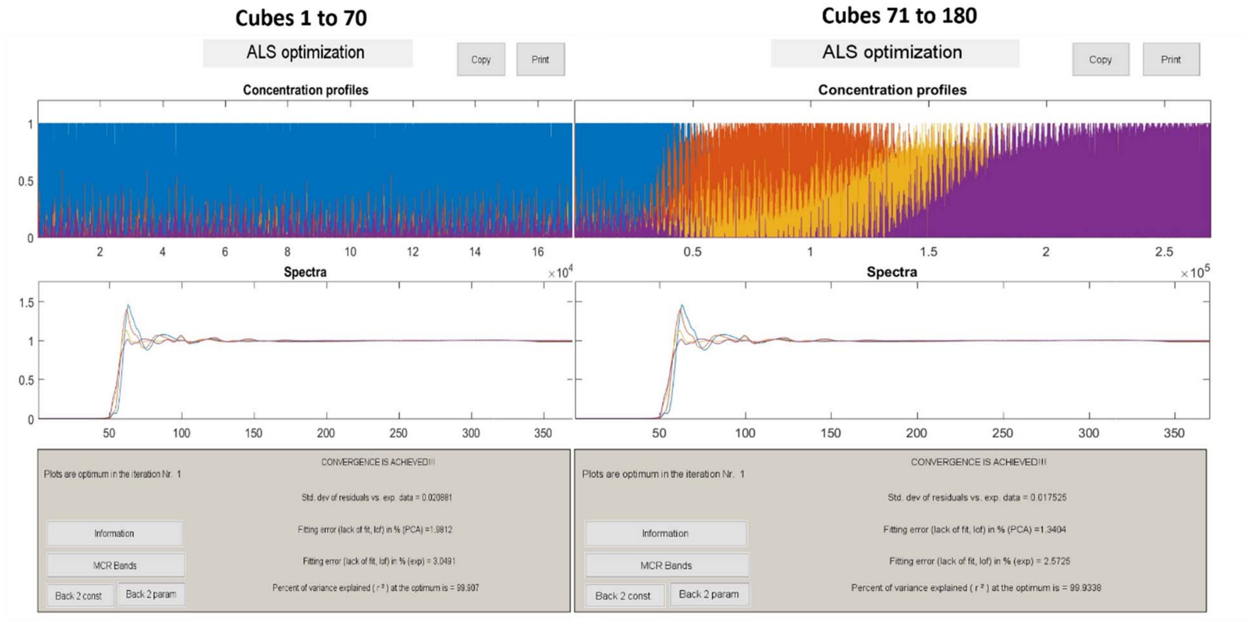

Figure S27 Outcomes of the MCR-ALS minimization at the Fe K edge of the flattened spectra extracted from the 180 hyperspectral cubes measured during heating between RT and 300°C of the bimetallic FeCu/SiO_2 catalyst under H_2. The minimization was carried out in two steps: i) MCR-ALS analysis of spectra isolated from cubes 71 to 180 (containing most of the variance) has been performed by considering as guessed S matrix the matrix of spectra obtained from the data set shown in Figure 14(b) and displayed in Figure S23 and as constraints: non-negativity of C and closure relation for C and ii) the MCR-ALS analysis of spectra isolated from cubes 1 to 70 has been performed by guessing for S the matrix of spectra obtained by minimizing the data set corresponding to cubes 71 to 180.

Constraints: non negativity of C and S, closure relation for C and lower or equal constraint with the guessed S matrix.

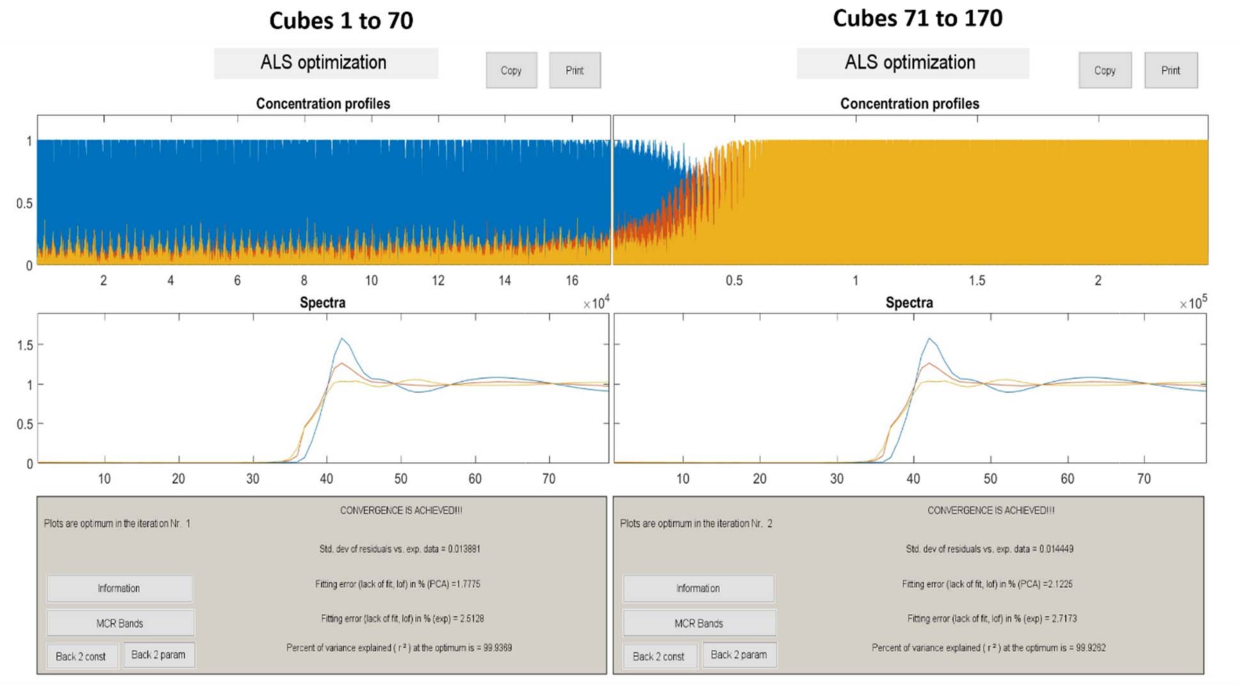

Figure S28 Outcomes of the MCR-ALS minimization at the Cu K edge of the flattened spectra extracted from the 170 hyperspectral cubes measured during heating between RT and 300°C of the bimetallic FeCu/SiO_2 catalyst under H_2. The minimization was carried out in two steps: i) MCR-ALS analysis of spectra isolated from cubes 71 to 170 (containing most of the variance) has been performed by considering as guessed S matrix the matrix of spectra obtained from the data set shown in Figure 14(d) and displayed in Figure S25 and as constraints: non-negativity of C and closure relation for C and ii) the MCR-ALS analysis of spectra isolated from cubes 1 to 70 has been performed by guessing for S the matrix of spectra obtained by minimizing the data set corresponding to cubes 71 to 170. Constraints: non negativity of C and S, closure relation for C and lower or equal constraint with the guessed S matrix.

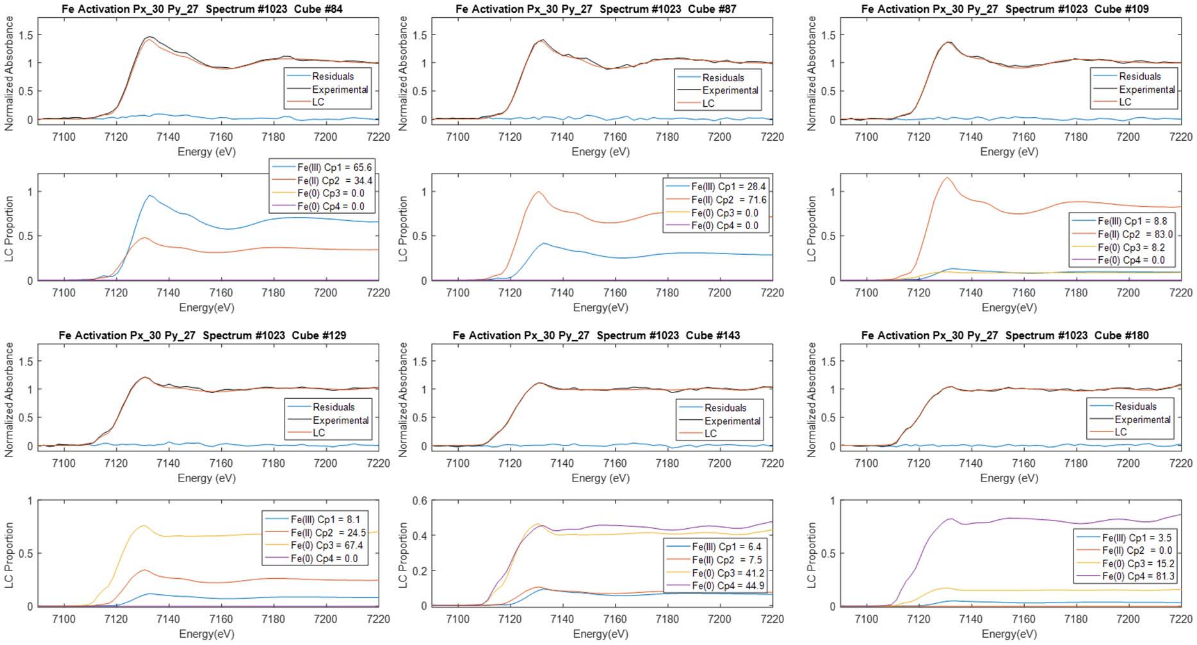

Figure S29 Comparison for some cubes recorded during the monitoring of the activation of the FeCu catalyst of the Fe K edge experimental spectrum (black line) recorded for the same pixel with the MCR-ALS rebuilt spectrum (red line) and corresponding residuals (blue line) corresponding to the difference between the experimental spectrum and rebuilt one. The weighted spectral contribution of each component used to rebuild each spectrum is also reported at the bottom plot.

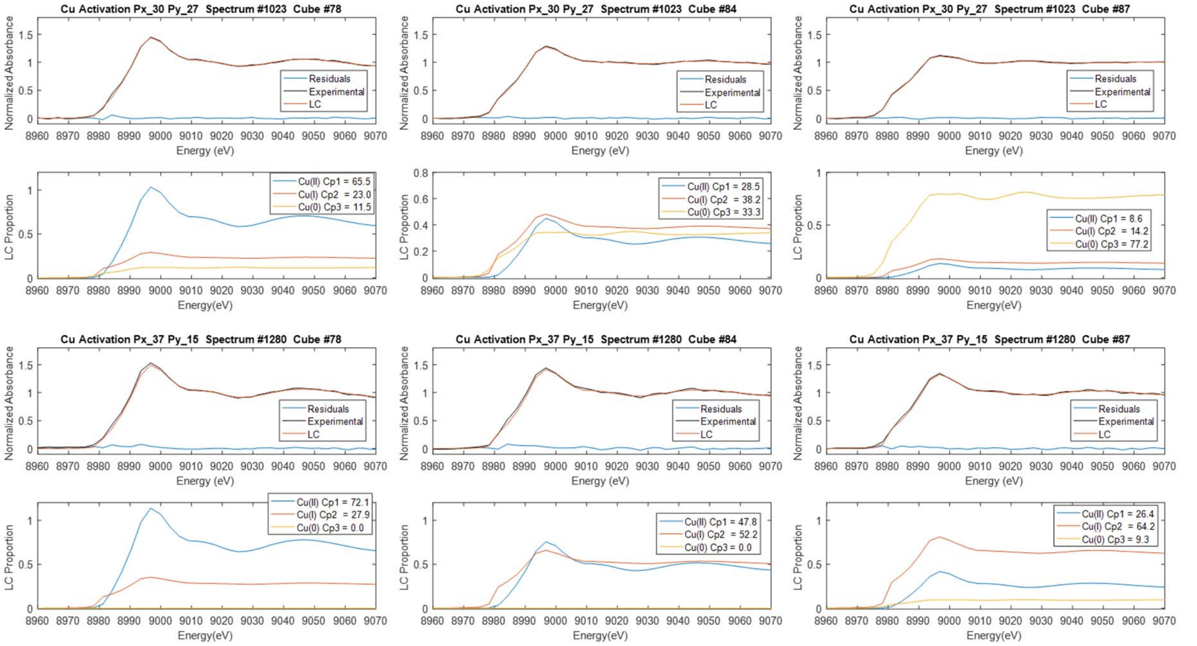

Figure S30 Comparison for some cubes recorded during the monitoring of the activation of the FeCu catalyst of the Cu K edge experimental spectrum (black line) recorded for two different pixels with the MCR-ALS rebuilt spectrum (red line) and corresponding residuals (blue line) corresponding to the

difference between the experimental spectrum and rebuilt one. The weighted spectral contribution of each component used to rebuild each spectrum is also reported at the bottom plot.

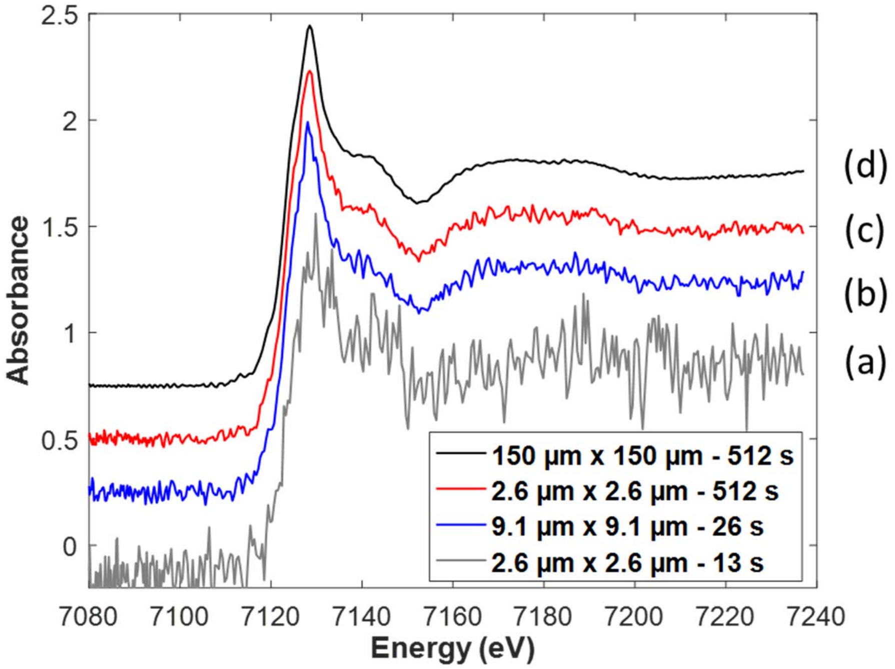

Figure S31 Comparison for the spin transition complex at the Fe K edge (case study 1) of spectra obtained with different cube merging and different pixel binning. (a) no cube merged and 4 x 4 pixel binning, (b) 2 cubes merged and 14 x 14 pixel binning, (c) 40 cubes merged and 4 x 4 pixel binning and (d) 40 cubes merged and all pixels binned over the image.

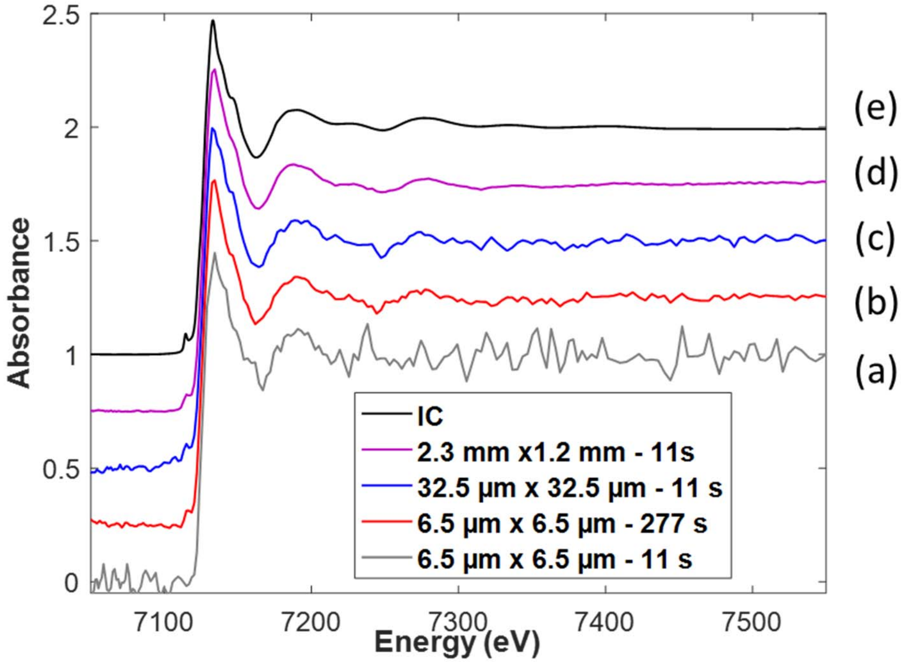

Figure S32 Comparison for the FeCu catalyst at the Fe K edge (case study 3) of spectra obtained with different cube merging and different pixel binning by FF Quick-EXAFS imaging: (a) no cube merged and 4 x 4 pixel binning, (b) 25 cubes merged and 4 x 4 pixel binning, (c) no cube merged and 20 x 20 pixel binning, (d) all pixels binned over the image and no cube merged. For comparison purpose in (e) is reported the spectrum recorded with the ICs by standard Quick-EXAFS.
